# Supplementary material for: The fossil insect assemblage associated with the Toarcian (Lower Jurassic) oceanic anoxic event from Alderton Hill, Gloucestershire, UK
Source: PLoS One. 2024 Apr 17;19(4):e0299551. doi: 10.1371/journal.pone.0299551 (PMC11023202; doi:10.1371/journal.pone.0299551)
Supplement: Attachment — Submitted filename: comments to PONE-D-23-33017_reviewer.pdf [file pone.0299551.s001.pdf]

PLOS ONE

The fossil insect assemblage associated with the Toarcian (Lower Jurassic) Oceanic Anoxic Event from Alderton Hill, Gloucestershire, UK

--Manuscript Draft--

**Manuscript Number:**	PONE-D-23-33017
**Article Type:**	Research Article
**Full Title:**	The fossil insect assemblage associated with the Toarcian (Lower Jurassic) Oceanic Anoxic Event from Alderton Hill, Gloucestershire, UK
**Short Title:**	Fossil insects associated with the Toarcian Oceanic Anoxic Event from Alderton Hill, UK
**Corresponding Author:**	Emily J. Swaby<br>The Open University<br>Milton Keynes, Buckinghamshire UNITED KINGDOM
**Keywords:**	Fossil insects; Alderton Hill; Toarcian Oceanic Anoxic Event; palaeoentomology; Strawberry Bank; taxonomy; taphonomy
**Abstract:**	Extreme global warming and environmental changes associated with the Toarcian (Lower Jurassic) Oceanic Anoxic Event (T-OAE, ~183 Mya) profoundly impacted marine organisms and terrestrial plants. Despite the exceptionally elevated abundances of fossil insects from strata of this age, only assemblages from Germany have been studied in detail. Here, we focus on the insect assemblage found in strata recording the T-OAE at Alderton Hill, Gloucestershire, UK, where <15% of specimens have previously been described. We located all known fossil insects (n = 370) from Alderton Hill, and used these to create the first comprehensive taxonomic and taphonomic analysis of the entire assemblage. We show that a diverse palaeoentomofaunal assemblage is preserved, comprising 12 orders, 20 families, 24 genera and 21 species. Fossil disarticulation is consistent with present-day insect decay studies. Ordinal diversity is comparable with present-day insect assemblages from similar latitudes (30°–40°N), including the Azores, and suggests that the palaeoentomofauna reflects a life assemblage. Hemiptera, Coleoptera and Orthoptera are the commonest (56.1%) orders. The high abundance of Hemiptera (22.1%) and Orthoptera (13.4%) indicates well-vegetated islands, while floral changes related to the T-OAE may be responsible for hemipteran diversification. Predatory insects are relatively abundant (~10% of the total assemblage) and we hypothesise that the co-occurrence of fish and insects within the T-OAE represents a jubilee event. The marginally higher proportion of highly sclerotised taxa compared to present-day possibly indicates adaptation to environmental conditions or taphonomic bias. The coeval palaeoentomofauna from Strawberry Bank, Somerset is less diverse (9 orders, 12 families, 7 genera, 3 species) and strongly taphonomically biased. The Alderton Hill palaeoentomofauna is interpreted to be the best-preserved and most representative insect assemblage from Toarcian strata in the UK. This study provides an essential first step towards understanding the likely influence of the T-OAE on insects
**Order of Authors:**	Emily J. Swaby
	Angela L. Coe
	Jörg Ansorge
	Bryony A. Caswell
	Scott A. L. Hayward
	Luke Mander
	Liadan G. Stevens
	Aimee McArdle
**Additional Information:**	
**Question**	**Response**

Powered by Editorial Manager® and ProduXion Manager® from Aries Systems Corporation

**Financial Disclosure**<br><br>Enter a financial disclosure statement that describes the sources of funding for the work included in this submission. Review the submission guidelines for detailed requirements. View published research articles from *PLOS ONE* for specific examples.<br><br>This statement is required for submission and **will appear in the published article** if the submission is accepted. Please make sure it is accurate.<br><br>**Funded studies**<br>Enter a statement with the following details:<br>• Initials of the authors who received each award<br>• Grant numbers awarded to each author<br>• The full name of each funder<br>• URL of each funder website<br>• Did the sponsors or funders play any role in the study design, data collection and analysis, decision to publish, or preparation of the manuscript?<br><br>Did you receive funding for this work?	Yes
Please add funding details.<br>as follow-up to "**Financial Disclosure**<br><br>Enter a financial disclosure statement that describes the sources of funding for the work included in this submission. Review the submission guidelines for detailed requirements. View published research articles from *PLOS ONE* for specific examples.<br><br>This statement is required for submission and **will appear in the published article** if the submission is accepted. Please make sure it is accurate.	ES<br>Grant number NE/S007350/1]<br>Natural Environmental Research Council (NERC) through the Central England NERC Training Alliance (CENTA) Doctoral Training Partnership<br>https://www.ukri.org/councils/nerc/<br>The sponsors did not play any role in the study design, data collection and analysis, decision to publish, or preparation of the manuscript.

Powered by Editorial Manager® and ProduXion Manager® from Aries Systems Corporation

**Funded studies**<br>Enter a statement with the following details:<br>• Initials of the authors who received each award<br>• Grant numbers awarded to each author<br>• The full name of each funder<br>• URL of each funder website<br>• Did the sponsors or funders play any role in the study design, data collection and analysis, decision to publish, or preparation of the manuscript?<br><br>Did you receive funding for this work?"	
Please select the country of your main research funder (please select carefully as in some cases this is used in fee calculation).<br>as follow-up to "**Financial Disclosure**<br><br>Enter a financial disclosure statement that describes the sources of funding for the work included in this submission. Review the submission guidelines for detailed requirements. View published research articles from *PLOS ONE* for specific examples.<br><br>This statement is required for submission and **will appear in the published article** if the submission is accepted. Please make sure it is accurate.<br><br>**Funded studies**<br>Enter a statement with the following details:<br>• Initials of the authors who received each award<br>• Grant numbers awarded to each author<br>• The full name of each funder<br>• URL of each funder website<br>• Did the sponsors or funders play any role in the study design, data collection and analysis, decision to publish, or preparation of the manuscript?<br><br>Did you receive funding for this work?"	UNITED KINGDOM - GB
**Competing Interests**	The authors have declared that no competing interests exist.

Use the instructions below to enter a competing interest statement for this submission. On behalf of all authors, disclose any competing interests that could be perceived to bias this work—acknowledging all financial support and any other relevant financial or non-financial competing interests.<br><br>This statement is required for submission and **will appear in the published article** if the submission is accepted. Please make sure it is accurate and that any funding sources listed in your Funding Information later in the submission form are also declared in your Financial Disclosure statement.<br><br>View published research articles from *PLOS ONE* for specific examples.<br><br>**NO authors have competing interests**<br><br>Enter: *The authors have declared that no competing interests exist.*<br><br>**Authors with competing interests**<br><br>Enter competing interest details beginning with this statement:<br><br>*I have read the journal's policy and the authors of this manuscript have the following competing interests: [insert competing interests here]*<br><br>* typeset	
**Ethics Statement**<br><br>Enter an ethics statement for this submission. This statement is required if the study involved:<br><br>• Human participants<br>• Human specimens or tissue<br>• Vertebrate animals or cephalopods<br>• Vertebrate embryos or tissues	N/A

• Field research<br><br>Write "N/A" if the submission does not require an ethics statement.<br><br>General guidance is provided below. Consult the submission guidelines for detailed instructions. **Make sure that all information entered here is included in the Methods section of the manuscript.**	

Powered by Editorial Manager® and ProduXion Manager® from Aries Systems Corporation

**Format for specific study types**<br><br>**Human Subject Research (involving human participants and/or tissue)**<br>• Give the name of the institutional review board or ethics committee that approved the study<br>• Include the approval number and/or a statement indicating approval of this research<br>• Indicate the form of consent obtained (written/oral) or the reason that consent was not obtained (e.g. the data were analyzed anonymously)<br><br>**Animal Research (involving vertebrate animals, embryos or tissues)**<br>• Provide the name of the Institutional Animal Care and Use Committee (IACUC) or other relevant ethics board that reviewed the study protocol, and indicate whether they approved this research or granted a formal waiver of ethical approval<br>• Include an approval number if one was obtained<br>• If the study involved *non-human primates*, add *additional details* about animal welfare and steps taken to ameliorate suffering<br>• If anesthesia, euthanasia, or any kind of animal sacrifice is part of the study, include briefly which substances and/or methods were applied<br><br>**Field Research**<br><br>Include the following details if this study involves the collection of plant, animal, or other materials from a natural setting:<br>• Field permit number<br>• Name of the institution or relevant body that granted permission	
**Data Availability**<br><br>Authors are required to make all data underlying the findings described fully available, without restriction, and from the time of publication. PLOS allows rare exceptions to address legal and ethical concerns. See the PLOS Data Policy and FAQ for detailed information.	Yes - all data are fully available without restriction

A Data Availability Statement describing where the data can be found is required at submission. Your answers to this question constitute the Data Availability Statement and **will be published in the article**, if accepted.<br><br>**Important:** Stating 'data available on request from the author' is not sufficient. If your data are only available upon request, select 'No' for the first question and explain your exceptional situation in the text box.<br><br>Do the authors confirm that all data underlying the findings described in their manuscript are fully available without restriction?	
**Describe where the data may be found in full sentences. If you are copying our sample text, replace any instances of XXX with the appropriate details.**<br><br>• If the data are **held or will be held in a public repository**, include URLs, accession numbers or DOIs. If this information will only be available after acceptance, indicate this by ticking the box below. For example: *All XXX files are available from the XXX database (accession number(s) XXX, XXX.*).<br>• If the data are all contained **within the manuscript and/or Supporting Information files**, enter the following: *All relevant data are within the manuscript and its Supporting Information files.*<br>• If neither of these applies but you are able to provide **details of access elsewhere**, with or without limitations, please do so. For example:<br><br>*Data cannot be shared publicly because of [XXX]. Data are available from the XXX Institutional Data Access / Ethics Committee (contact via XXX) for researchers who meet the criteria for access to confidential data.*<br><br>*The data underlying the results presented in the study are available from (include the name of the third party*	All data files and photographs are available from the Dryas database at: https://datadryad.org/stash/share/0g_D75vgw0G5e1dMfmQEEEMZYVPTKErCpD9b67Jr8rs

and contact information or URL).<br>• This text is appropriate if the data are owned by a third party and authors do not have permission to share the data.<br><br>* typeset	
Additional data availability information:	Tick here if the URLs/accession numbers/DOIs will be available only after acceptance of the manuscript for publication so that we can ensure their inclusion before publication.

Powered by Editorial Manager® and ProduXion Manager® from Aries Systems Corporation

Manuscript

Click here to access/download;Manuscript;Swaby et al. Alderton Hill manuscript - for submission.docx

The fossil insect assemblage associated with the Toarcian (Lower Jurassic) Oceanic Anoxic Event from Alderton Hill, Gloucestershire, UK

Short title: Fossil insects associated with the Toarcian Oceanic Anoxic Event from Alderton Hill, UK

Emily J. Swaby[1]*, Angela L. Coe[1], Jörg Ansorge[2], Bryony A. Caswell[3], Scott A. L. Hayward[4,5], Luke Mander[1], Liadan G. Stevens[6] and Aimee McArdle[6]

[1] School of Environment, Earth and Ecosystem Sciences, Faculty of Science, Technology, Engineering and Mathematics, The Open University, Milton Keynes, Buckinghamshire, UK.

[2] Institut für Geologische Wissenschaften, Ernst-Moritz-Arndt-Universität Greifswald, Greifswald, Germany.

[3] School of Environmental Science, Faculty of Science and Engineering, University of Hull, UK.

[4] School of Biosciences, University of Birmingham, Birmingham, UK.

[5] Birmingham Institute of Forest Research, University of Birmingham, Birmingham, UK.

[6] Natural History Museum, London, UK.

ORCID ID: EJS, 0000-0001-7436-5641; ALC, 0000-0003-1416-5052; JA, 0000-0002-1284-6893; BAC, 0000-0001-8488-0890; SALH, 0000-0002-1899-6630; LM, 0000-0003-4347-2705; LGS, 0000-0001-6557-4302.

*Corresponding author

E-mail: emily.swaby@open.ac.uk (EJS)

Abstract

Extreme global warming and environmental changes associated with the Toarcian (Lower Jurassic) Oceanic Anoxic Event (T-OAE, ~183 Mya) profoundly impacted marine organisms and terrestrial plants. Despite the exceptionally elevated abundances of fossil insects from strata of this age, only assemblages from Germany have been studied in detail. Here, we focus on the insect assemblage found in strata recording the T-OAE at Alderton Hill, Gloucestershire, UK, where <15% of specimens have previously been described. We located all known fossil insects (n = 370) from Alderton Hill, and used these to create the first comprehensive taxonomic and taphonomic analysis of the entire assemblage. We show that a diverse palaeoentomofaunal assemblage is preserved, comprising 12 orders, 20 families, 24 genera and 21 species. Fossil disarticulation is consistent with present-day insect decay studies. Ordinal diversity is comparable with present-day insect assemblages from similar latitudes (30°–40°N), including the Azores, and suggests that the palaeoentomofauna reflects a life assemblage. Hemiptera, Coleoptera and Orthoptera are the commonest (56.1%) orders. The high abundance of Hemiptera (22.1%) and Orthoptera (13.4%) indicates well-vegetated islands, while floral changes related to the T-OAE may be responsible for hemipteran diversification. Predatory insects are relatively abundant (~10% of the total assemblage) and we hypothesise that the co-occurrence of fish and insects within the T-OAE represents a jubilee event. The marginally higher proportion of highly sclerotised taxa compared to present-day possibly indicates adaptation to environmental conditions or taphonomic bias. The coeval palaeoentomofauna from Strawberry Bank, Somerset is less diverse (9 orders, 12 families, 7 genera, 3 species) and strongly taphonomically biased. The Alderton Hill palaeoentomofauna is interpreted to be the best-preserved and most representative insect assemblage from Toarcian strata in the UK. This study provides an essential first step towards understanding the likely influence of the T-OAE on insects.

Introduction

Evidence for present-day anthropogenically-driven climate change is overwhelming [1], and contemporary global warming is significantly altering biodiversity and Earth's ecosystems at an unprecedented rate and scale [2]. Insects are the most affected groups of animals, with many insect taxa currently experiencing rapid declines [3]. Changes in climate greatly affect the physiology, diversity, reproduction and population size of insects [3, 4, 5, 6, 7]. However, despite the importance of insects within terrestrial ecosystems, research into understanding how they are, and will, respond to a rapidly changing climate is still in its infancy [8, 9]. Accumulations of fossil insects provide opportunities to investigate the dynamics of insect diversity and ecology through time including how they have responded to previous environmental change. Here, we focus on a previously largely undescribed assemblage of fossil insects from the UK that is associated with the extreme climate change during the Toarcian Oceanic Anoxic Event (T-OAE), Early Jurassic (~183 Ma ago), to provide base-line information for further studies on the impact of this climatic event on insects.

It is now well established that the T-OAE was a sudden and severe global event of extreme environmental change, associated with widespread burial of marine carbon and a pronounced perturbation to the global carbon cycle [10, 11, 12, 13, 14]. Studies of records of the T-OAE have highlighted synchronous evidence for increased global palaeotemperatures [15, 16, 17, 18, 19, 20, 21, 22, 23, 24], enhanced global chemical weathering rates [25, 26], widespread marine anoxia [27] and increased atmospheric CO_2 levels [28].

The T-OAE had a profound influence on marine biota, including elevated mass extinction rates [29, 30], changes in biogeographical range shifts of key marine taxa [31, 32], as well as changes in seafloor community composition and the body size of seafloor organisms [32, 33, 34, 35, 36, 37, 38]. More recently it has been noted that on land, global warming, possible wildfires and acid rain associated with the T-OAE could have resulted in a decrease in land plant diversity [39], which then had significant effects on the rest of the trophic web including

extinction of all basal sauropodomorph dinosaurs [40]. Insects form a critical part of ecosystems, however their response to past periods of environmental change, including the T-OAE, has not previously been investigated in detail.

The insect material from the Toarcian (Lower Jurassic) strata of Western Europe reported to date is all preserved in marine deposits and is stratigraphically restricted to the lower Toarcian (*Cleviceras exaratum* ammonite Subzone, lower *Harpoceras falciferum* ammonite Zone), coinciding with the negative carbon isotope excursion that characterises the T-OAE [11]. This relationship, in addition to the lack of fossil insect accumulations in the strata above and below and their rarity in stratigraphical record in general, strongly suggests that their occurrence may be linked to palaeoenvironmental and palaeoecological conditions of the T-OAE [41]. Abundant insect material associated with the T-OAE has been found in the UK (Alderton Hill, Gloucestershire [42], and Strawberry Bank, Somerset [43, 44, 45]), Northern Germany (e.g. Grimmen, Dobbertin and Braunschweig [46, 47, 48, 49]), Southern Germany (e.g. Kerkhofen, Holzmaden and Mistelgau [50, 51, 52]) and Luxembourg (e.g. Bascharage and Sanem [41, 53, 54, 55, 56, 57, 58]). Smaller assemblages of Toarcian insect material have also been documented from Switzerland [59] and Belgium [60].

This study focuses on the early Toarcian succession of Alderton Hill, Gloucestershire, UK (SP006345) that yielded a rich and diverse insect fauna when it was exposed in the mid-19th century [61, 62, 63, 64]. The succession is regarded as an important locality for Early Jurassic insect faunas [42] and has provided the type specimens of 30 Early Jurassic insect species. However, despite discovery more than 170 years ago, the specimens, now in museum collections, have only received limited scientific study that has focused on the taxonomy of selected orders. Furthermore, a comprehensive synthesis and review of all insects from the locality has yet to be published [42, 51, 65]. Selected specimens from the Alderton Hill palaeoentomofauna were initially described during the late 19th century [66, 67, 68, 69] and early 20th century [70, 71, 72, 73]. In recent literature, only specimens from certain insect orders have been featured in publications, including: Odonata [74, 75], Blattodea [76], Diptera

[77] and Necrotrichoptera [45, 78]. Most recently, Simms [65] based almost entirely on historical records [79, 80], provided a brief summary of the Alderton Hill succession, while Jarzembowski and Palmer [42], provided a list of the insect orders present based on the literature.

The only other known UK locality that has yielded a substantial amount of fossilized insects from the T-OAE is Strawberry Bank in Ilminster, Somerset (ST361148). This location has yielded approximately 800 individual insect fossils [43, 64, 81], in addition to a diverse assemblage of marine nekton, including ichthyosaurs [82, 83, 84, 85], crocodiles [81, 86], fishes [87], cephalopods [88], crustaceans [89] and molluscs [90]. The diverse and well-preserved fauna was exclusively collected by Charles Moore during the late 1840s [43, 64] and the original locality is now inaccessible [83]. In 2019, a new temporary excavation of Strawberry Bank was undertaken by the Bath Royal Literary and Scientific Institution and University of Bristol and research on this new exposure is on-going [90]. The original insect material from Strawberry Bank collected by Moore was briefly mentioned by Brodie [64] but remained undescribed until Williams *et al*. [43] provided an overview.

Here, for the first time, we provide a detailed synthesis, description and analysis of the taxonomy and taphonomy of all known fossil insect material from Alderton Hill, that we have located within UK museum and institution collections. We compare the Alderton Hill insect assemblage with the contemporaneous UK fossil insects from Strawberry Bank and present-day insect assemblages in order to demonstrate the taxonomic diversity, preservation and significance of the Toarcian Alderton Hill palaeoentomofauna. This study will provide an essential baseline for future studies to assess the impact of the T-OAE on insects and how global warming influences their critical ecological role within the biosphere.

Location and geological setting

The Early Jurassic deposits that comprise Alderton Hill, Gloucestershire, UK were deposited within the Severn Basin, a north-south elongated basin extending approximately 50 km east-west and ≥ 90 km north-south [65], centred on what is now the town of Tewksbury. During the Early Toarcian, Gloucestershire was located at palaeolatitude of 35°– 40°N (Fig 1A), and the Severn Basin formed a small part of an extensive epicontinental shelf with several small islands (e.g. London-Brabant Massif, Cornubian Massif and the Welsh Massif; Fig 1B). The major landmasses of Laurentia and Eurasia were > 1000 km away (Fig 1B). Today, Alderton Hill is located approximately 2 km southwest of the village of Dumbleton, Gloucestershire. Alderton Hill originally had three small quarries in a small area: Alderton Hill Quarry, Dumbleton Pit and Naunton Farm Quarry. However, the whole area is now extensively overgrown with almost no visible exposure. The sites of all three quarries are situated on an area of private land protected both as a geological Site of Special Scientific Interest (SSSI) and a Geological Conservation Review site (GCR) [91].

Figure 1. (A) Global palaeogeographical map of the Early Toarcian (modified from [22]), showing the location of the Karoo-Ferrar large igneous province (LIP) in South Gondwana, and, as dashed lines, the position of palaeoclimatic belts [92]; the red box shows B. **(B)** Palaeogeography of NW Tethys modified from [93] showing the likely distribution of the Toarcian palaeo-landmasses superimposed on the present-day coastline, and the location of: **(1)** Alderton Hill, UK and **(2)** Strawberry Bank, UK. The palaeo-landmasses ordered by palaeolatitude are: F = Fennoscandia; GH = Grampian High; FH = Fyn High; PH = Pennine High; HBM = Hercynian-Bohemian Massif; RM = Rhenic Massif; LBM = London-Brabant Massif; IM = Irish Massif; WM = Welsh Massif; CM = Cornubia Massif; AS = Alemannic Swell; AM = Armorican Massif; MC = Massif Central; IM = Iberian Massif. Figure modified from [94, 95, 96].

Stratigraphically, Alderton Hill comprises the Marlstone Rock Formation (Upper Pliensbachian) overlain by the Dumbleton Member which forms the lowermost unit of the Whitby Mudstone Formation (Lower Toarcian) [65]. As the section has not been exposed for some time, we rely on the summary compiled by Simms [65] based on nineteenth century records (referenced therein) and Richardson [80]. Using this summary and our observations of the matrix around the fossil specimens, the Dumbleton Member is comprised of organic-rich, laminated blue-grey mudstones, with several bands of early diagenetic calcareous nodules, including the nodular Fish Bed (Bed 10) that occurs *c.* 4 m above the base of the Dumbleton Member.

The presence of the ammonites *Hildaites murleyi* (BGS GS.32040), *H. forte* (BGS GS.32052) and *Cleviceras elegans* (Fig 2G) within the Fish Bed indicate the horizon is situated within the *Cleviceras exaratum* Subzone*,* lower *Harpoceras falciferum* Zone [97, 98]. This is stratigraphically comparable with the insect-bearing Saurian and Fish Zone of the Strawberry Bank Lagerstätte [43] (Fig 1B). In addition to the abundant insect material, the Fish Bed at Alderton Hill has yielded a high abundance of the small early teleost fish *Leptolepis coryphaenoides*, *L. normandica* and *L. concentricus* (Figs A – 2C). During this study, a specimen assigned to *Pachycormus macropterus* (Fig 2D) was located in the Sedgwick Museum, Cambridge. There is also a marine invertebrate fauna dominated by nektonic and planktonic species, including teuthoids (Fig 2E, Figs 2I – J) [99, 100, 101]; rare arthropods including the holotype of *Eryon richardsoni* (Fig 2K) [102, 103]; molluscs, including the bivalve *Pseudomytiloides dubius* (Fig 2F) and an abundance of the small gastropod *Coelodiscus minutus* (Fig 2H) [42, 65]; together with rare reptile remains [82].

Figure 2. Fauna associated with the insect horizon at Alderton Hill, Gloucestershire, UK. Scale = 1 cm unless otherwise stated. **(A)** *Leptolepis concentricus* Egerton 1849 [64], BGS 114078-79 (Actinopterygii: Leptolepiformes); **(B)** *L. normandica* Nybelin 1962 [104], NHMUK PV P.7621, in association with indeterminate insect; **(C)** *L. coryphaenoides* Bronn 1830 [105], NHMUK PV P.28855; **(D)** *Pachycormus macropterus* de Blainville 1818 [106], CAMSM

J.61246 (Pachycormiformes: Pachycormidae), scale = 2 cm; **(E)** Hooklets of *Geoteuthis* Münster 1843 [107], BGS GSM 117962-63 (Cephalopoda), scale = 5 mm); **(F)** *Pseudomytiloides dubius* Sowerby 1823 [108], BGS Geol. Soc. 4147 (Bivalvia: Myalinida); **(G)** *Hildoceras bifrons* Bruguiere 1789 [109], NHMUK. PI C.6222 (Cephalopoda: Ammonitida); **(H)** *Coelodiscus minutus* Schubler 1833 [110], BGS GSM 114054 (Gastropoda: Coelodiscidae), scale = 2.5 mm; **(I)** *Chondroteuthis wunnenbergi* Bode 1933 [111], NHMUK PI C.5257a,b (Cephalopoda: Belemnitida); **(J)** *Chondroteuthis wunnenbergi* Bode 1933 [111], NHMUK PI C.59301 (Cephalopoda: Belemnitida); **(K)** *Eryon richardsoni* Woodward 1911 [102], CHAGM F.685, holotype (Decapoda: Eryonidae), scale = 2 cm.

The other UK location that has yielded extensive insect fossils, Strawberry Bank, was located in the town Ilminster, Somerset, approximately 130 km southwest of Alderton Hill. During the Jurassic, this location was within the Wessex Basin, that lay to the south and south east of the Severn Basin and encompassing most of southern England. Palaeogeographically, Strawberry Bank was similar to Alderton Hill, lying in the epicontinental seaway (Fig 1B). The sedimentary facies indicate that it is likely that Strawberry Bank was close to land [43]. The insect material originates from a single layer of pavement-like concretions of variable thickness, referred to as the Saurian and Fish Zone within the Barrington Member of the Beacon Limestone Formation [82, 90]. Similar to Alderton Hill, this horizon occurs within the *exaratum* Subzone of the *falciferum* Zone and therefore records the T-OAE [43]. The concretions from the Strawberry Bank succession are thought to be part of a localised deposit, as they are not observed within the Toarcian succession near to the village of Seavington St. Michael, approximately 4 km to the east of Ilminster [90, 112]. While much of the fauna from the Strawberry Bank is exceptionally well-preserved, including phosphatized soft tissue and three-dimensional preservation of marine nekton [90], giving the location its lagerstätte status, the insects are often poorly preserved, and this is attributed to the coarse-grained nature of the deposits [43].

Materials and methods

Three hundred and sixty-three numbered rock specimens, which possessed fossilised insects from Alderton Hill Quarry, Dumbleton Pit and Naunton Farm Quarry, were located in eight museum and institutional collections (Table 1). There are less specimens (n = 363) than individual insects (n = 370), as some specimens contained multiple individuals. The majority of the insect material from Alderton Hill was collected by Brodie and Slatter and subsequently acquired by the Natural History Museum, London (NHMUK) in the late 19th century. Additional specimens from Alderton Hill located and used in this study were: those collected by Holland of Dumbleton Hall, Gloucestershire [65, 113], that are now in the collections of the Wilson Museum, Cheltenham (CHAGM); those at the British Geological Survey, Keyworth (BGS), National Museum of Wales, Cardiff (NMW), Warwickshire Museum, Warwick (WARMS), Sedgwick Museum, Cambridge (CAMSM) and Museum of Comparative Zoology, Harvard University (PALE); and most recently, nine insect specimens collected in the 1970s and 1980s by M.J. Simms, which are now in the Bristol City Museum (BRSMG). A database was created of all the Alderton Hill insect specimens and the associated data, including the catalogue numbers, authors of the collection, taxonomy, type status, specimen descriptions and preservation for each fossil. For all material from NHMUK, specimen numbers are made up of a unique specimen number and a series of prefixes as follows: NHMUK – institution; PV – division (vertebrates); PI – division (invertebrates); I – section (insects); P – section (fish); C – section (cephalopods).

Three hundred and eighty-six numbered rock specimens, which possessed fossilised insects from the Strawberry Bank lagerstätte, were located within four museum and institutional collections (Table 2). Three hundred and fifty-nine insect specimens were examined within the collections of TTNCM; this material was originally collated by Williams *et al.* [43]. Additional specimens (n = 27) from Strawberry Bank were also discovered during this study within the collections of NHMUK (n = 18), CAMSM (n = 8) and BRSMG (n = 1). Again, there are less

specimens (n = 386) than individual insects (n = 799). All of the insect material from Strawberry Bank, including the associated taxonomic and taphonomic data, was also compiled into a database for comparison with the Alderton Hill palaeoentomofauna. The four Toarcian insect specimens from Alderton Hill, documented as being held at Warwickshire Museum [114] could not be re-examined, as the collections were inaccessible at the time of this study. An additional six specimens from the collections at NHMUK and one specimen at Somerset Heritage Centre, Taunton, UK (TTNCM) were not re-examined due to either being lost (one specimen: NHMUK PI I.3988) or on loan. Taxonomic data for these specimens is therefore based on either Kelly [114] or the original museum databases.

Table 1. Quantity of numbered specimens and locations of all individual Toarcian insect collections from the three Alderton Hill exposures, Gloucestershire, UK, including the name and abbreviations for each museum and institution collection.

**Museum / Institution**	**Number of specimens**			**Total**
	**Alderton Hill Quarry**	**Dumbleton Pit**	**Naunton Farm Quarry**	
Natural History Museum, London, UK (NHMUK)	87	202	0	**289**
Wilson Museum, Cheltenham, UK (CHAGM)	1	45	2	**48**
Bristol Museum & Art Gallery, Bristol, UK (BRSMG)	4	4	1	**9**
British Geological Survey, Keyworth, UK (BGS)	0	6	0	**6**
Sedgwick Museum,	5	0	0	**5**

Cambridge, UK (CAMSM)				
Warwickshire Museum, Warwick, UK (WARMS)	3	1	0	**4**
Museum of Comparative Zoology, Harvard University, USA (PALE)[a]	1	0	0	**1**
National Museum Wales, Cardiff, UK (NMW)	1	0	0	**1**
**Total**	**102**	**258**	**3**	**363**

[a] In addition to this specimen (PALE-8749), this collection also includes the counterpart (PALE-8751) to the holotype of *Paractinophlebia curtisii* (NHMUK PI I.3585).

Table 2. Quantity of numbered specimens and locations of all individual Toarcian insect collections from Strawberry Bank, Ilminster, Somerset, UK, including the abbreviations for each museum and institution collection.

**Museum / Institution**	**Number of specimens from Strawberry Bank**
Somerset Heritage Centre, Taunton, UK (TTNCM)	359
Natural History Museum, London, UK (NHMUK)	18
Sedgwick Museum, Cambridge, UK (CAMSM)	8
Bristol Museum & Art Gallery, Bristol, UK (BRSMG)	1
**Total**	**386**

Taxonomic identifications method

Prior to this study, most of the Alderton Hill insects within the museum collections had been given a preliminary identification on a label stored with the specimen (largely by E. A. Jarzembowski in the collections of NHMUK and CHAGM), but less than 15% of the specimens have previously been taxonomically described in the literature [see 67, 68, 69, 70, 71, 72, 73, 115]. Within this study, through direct observation and high-resolution photographs, the taxonomy of each specimen was reviewed. This review included an update of the taxonomic nomenclature, confirmation of preliminary identifications, and where possible, refinement to a finer taxonomic resolution. The Strawberry Bank insect material, held within the collections of TTNCM and originally identified by Williams *et al.* [43] was also re-examined as part of this study, and in some cases, the taxonomy of these insects was refined.

For both the Alderton Hill and Strawberry Bank collections, every fragment that could be identified as insect-derived was recorded in order to reduce collection bias. No additional specimen preparation was undertaken. Incomplete or poorly preserved material which could not confidently be assigned to a particular insect taxon was labelled ‘indeterminate’. Following open nomenclature convention [116], where the taxonomic identification of a specimen is uncertain, a ? is placed in front of the taxa name (e.g. ?Dermaptera). Where possible, insects were examined with a binocular stereomicroscope at the different institutes to assess both the taxonomy and individual taphonomic grade. Following standard entomological nomenclature [117 and references therein], taxonomic identifications were predominantly based on insect wings and their venation.

Accurate morphological data and measurements of the preserved length and width of specimens were recorded with digital callipers to the nearest 0.1 mm. For articulated specimens, length was measured from the anterior to the posterior preserved margin of the insect, whilst width was measured across the widest section. For isolated wings, length was measured from wing tip to base, whilst width was measured across the widest dimension.

However, minor vertical compression of the material during fossilization may have resulted in limited distortion, meaning that they don't accurately reflect those in life. The orientation (dorsoventral or lateral position) of each insect was also recorded. The feeding habits of the Alderton Hill insects were determined by direct comparison with present-day taxa [e.g. 117] where possible, whereas the feeding habits of the Strawberry Bank insects could not be inferred as most specimens could not be identified to sufficient taxonomic level.

As the collections at NHMUK contained the majority of the insect material, including many well-preserved specimens, all of the Alderton Hill and Strawberry Bank insects were photographed using a Canon EOS 5DS R with a single head light source with an adjacent reflector in order to best capture the preserved wing venation. Within the smaller collections e.g. CHAGM, BRSMG and BGS, the insect material was often poorly preserved and fragmentary, and therefore only well-preserved specimens were photographed. The Strawberry Bank insects at TTNCM were not photographed due to the poor and fragmentary preservation of the majority of the material; the best preserved examples are figured in Williams *et al.* [43]. Detailed photographs were also taken of associated fauna from Alderton Hill within the collections of NHMUK and CAMSM (camera model: Nikon D3200 with Nikon Micro-Nikkor 40mm F/2.8 macro lens). A specimen from each insect order, family, genus and species from Alderton Hill has been incorporated into palaeontological plates using CorelDRAW Graphics Suite X8 (Figs 3 - 5). In each case, we have chosen the best preserved specimen within each taxa. No plates are provided for the new Strawberry Bank specimens, due to the poor preservation of the material. Photographs of all the Toarcian insect material taken during this study are provided in supplementary materials.

Figure 3. Fossil insect orders Odonata, ?Dermaptera, Reculida, Blattodea and Orthoptera from the Toarcian of Alderton Hill, Gloucestershire. **(A)** *Heterophlebia buckmani* Brodie 1845 [63] (Odonata: Heterophlebiidae), NHMUK PI I.11310; **(B)** *Heterothemis brodiei* Buckman 1843 [61] (Odonata: Liassogomphidae), NHMUK PI I.3552 (holotype); **(C)** *H. brodiei* Buckman 1843 [61], NHMUK PI I.11288; **(D)** *Protomyrmeleon* sp. Geinitz 1888 [118] (Odonata:

Protomyrmeleontidae), NHMUK PI In.59065; **(E)** ?Dermaptera De Geer 1773 [119], NHMUK PI I.11403; **(F)** *Geinitzia carpentieri* Zeuner 1937 [72] (Reculida: Geinitziidae), NHMUK PI In.36200 (holotype); **(G)** *Liadoblattina blakei* Scudder 1886 [68] (Blattodea: Raphidiomimidae), NHMUK PI I.3574 (holotype); **(H)** Blattodea Latreille 1810 [120], *incertae sedis,* BRSMG Cg2374; **(I)** *Locustopsis* sp. Handlirsch 1906 [121] (Orthoptera: Locustopsidae), NHMUK PI I.11370; **(J)** "*Elcana*" cf. *geinitzi* Heer 1880 [122] (Orthoptera: Elcanidae), NHMUK PI I.11295; **(K)** "*Elcana*" cf. *geinitzi* Heer 1880 [122], NHMUK PI I.3560; **(L)** *Protogryllus magnus* Zeuner 1937 [72] (Orthoptera: Protogryllidae), NHMUK PI I.11324 (holotype); **(M)** Orthoptera Olivier 1789 [123], *incertae sedis*, NHMUK PI I.11405. Scale bars = 2.5 mm.

Figure 4. Fossil insect orders Hemiptera, Coleoptera, Necrotrichoptera, Mecoptera from the Toarcian of Alderton Hill, Gloucestershire. **(A)** *Fulgoridium* sp. Handlirsch 1906 [121] (Hemiptera: Fulgoridiidae), NHMUK PI I.11328; **(B)** *Margaroptilon brodiei* Handlirsch 1906 [121] (Hemiptera: Fulgoridiidae), NHMUK PI I.3561 (holotype); **(C)** *M. brodiei* Handlirsch 1906 [121], NHMUK PI I.11266 (previously holotype of *M. bulleni*); **(D)** *Archijassus heeri* Geinitz 1880 [124], (Hemiptera: Archijassidae), NHMUK PI I.11458; **(E)** Progonocimicidae Handlirsch 1906 [121] (Hemiptera), NHMUK PI I.11465; **(F)** *Engynabis tenuis* Bode 1953 [48] (Hemiptera), NHMUK PI I.3313; **(G)** Hemiptera Linnaeus 1758 [125], *incertae sedis,* NHMUK PI I.11446; **(H)** Hemiptera Linnaeus 1758 [125], *incertae sedis,* CHAGM F.643.1; **(I)** Hemiptera Linnaeus 1758 [125], *incertae sedis,* NHMUK PI I.11317; specimen photographed and reproduced with permission; **(J)** Coleoptera Linnaeus 1758 [125], *incertae sedis,* NHMUK PI I.69; **(K)** Coleoptera Linnaeus 1758 [125], *incertae sedis,* NHMUK PI I.11345; **(L)** Coleoptera Linnaeus 1758 [125], *incertae sedis,* NHMUK PI I.3077; **(M)** Coleoptera Linnaeus 1758 [125], *incertae sedis,* NHMUK PI I.11355; **(N)** *Necrotaulius parvulus* Geinitz 1884 [67] (Necrotrichoptera: Necrotauliidae), NHMUK PI I.15014; **(O)** *N. parvulus* Geinitz 1884 [67], NHMUK PI I.11389; **(P)** *Protobittacus handlirschi* Tillyard 1933 [71] (Mecoptera: Bittacidae), NHMUK PI I.11406 (holotype); **(Q)** *Orthophlebia brodiei* Tillyard 1933 [71] (Mecoptera:

Orthophlebiidae), NHMUK PI I.15019 (holotype); **(R)** *Orthophlebia* sp. Westwood 1845 [115] NHMUK PI I.11225. Scale bars = 2.5 mm.

Figure 5. Fossil insect orders Neuroptera and Diptera from the Toarcian of Alderton Hill, Gloucestershire. **(A, B)** *Actinophlebia intermixta* Scudder 1885 [126] (Neuroptera: Osmylopsychopidae), NHMUK PI I.3577 & NHMUK PI I.11346 (holotype, part and counterpart); **(C)** *Paractinophlebia curtisii* Scudder 1886 [68] (Neuroptera: "Familia nova A"), NHMUK PI I.3585 (holotype); **(D)** *"Prohemerobius" aldertonensis* Whalley 1988 [69] (Neuroptera: "Prohemerobiidae"), NHMUK PI I.11304 (holotype); **(E)** *"Archeosmylus" alysius* Whalley 1988 [69] (Neuroptera: "Prohemerobiidae"), NHMUK PI I.3318 (holotype); **(F)** *"A." complexus* Whalley 1988 [69] (Neuroptera: "Prohemerobiidae"), NHMUK PI I.11412 (holotype); **(G)** *Architipula anglicana* Tillyard 1933 [71] (Diptera: Limoniidae), NHMUK PI I.11298 (holotype); **(H)** *Grimmenia tillyardi* Kopeć et al. 2017b [77] (Diptera: Limoniidae), NHMUK PI I.3328 (holotype). Scale bars = 2.5 mm.

Taphonomic classification method

The completeness and articulation of each insect specimen from Alderton Hill and Strawberry Bank was individually assessed using a standard semiquantitative method of grading the preservational quality (modified from [126]; Table 3). An alphanumeric code reflecting preservational-quality was assigned to each individual based on the preserved morphological features, including the presence/absence of wings and appendages and their completeness, as explained below.

The specimens were first divided into three groups based on the degree of disarticulation and indeterminability (Table 3). The first group (1), including all specimens which possessed a body with forewings and/or hindwings, was then further subdivided into three secondary taphonomic grades based on the degree of completeness; grades ranged from 1.1 through 1.3, meaning that specimens assigned to 1.1 were of the highest quality of preservation and

1.3 the poorest. The second group (2) included four subgroups: isolated bodies (2a), forewing/s (2b), hindwings (2c) and unassigned wings (2d). Each subgroup was then further divided into two taphonomic grades, designated .1 and .2, based on the degree of completeness of each insect element (Table 3). Unidentifiable insect fragments could not be used for further analysis and therefore were assigned to the third group (labelled '??'). Insect orders which only possessed one pair of wings e.g. Diptera were included within 2b. As many specimens possessed a part and counterpart which often displayed varying taphonomic grades, the taphonomic assessment was based on the better-preserved one. To prevent researcher bias, by which an insect could be considered better preserved because it had been previously assigned to a taxon, any associated taxonomic information was not examined until after taphonomic grades had been assigned to the specimens.

Statistical analysis

As taphonomic grades can be influenced by the anatomy of certain insects creating a preservational bias, a Chi-squared statistical test was performed in PAST 4.03 [127] to quantify and compare the association between each of the taphonomic grades and order diversity. In order to determine the degree to which each primary taphonomic grade contributed to the total Chi-squared score at order level, Pearson residuals were calculated for each entry in the Chi-squared contingency table at the order level using the statistics programme R (version 4.2.0).

Table 3. Description of the basis for designation of insect material into the primary and secondary taphonomic grades. Modified from [126].

	**Primary taphonomic grades**	**Secondary taphonomic grades**

Level of articulation	Grade	Description	Grade	Description
**Articulated material**	**1**	Body with forewings and/or hindwings	**1.1**	Complete body; with appendages
			**1.2**	Complete body; without appendages
			**1.3**	Body deformed; head, thorax, or abdomen lost / indistinguishable
**Disarticulated material**	**2a**	Isolated body	**2a.1**	Almost complete body (≥75% complete)
			**2a.2**	Skeletal body element(s) e.g. isolated body fragments, leg etc.
	**2b**	Isolated forewing/s	**2b.1**	Almost complete forewing/s (≥75% complete)
			**2b.2**	Forewing fragment/s
	**2c**	Isolated hindwing/s	**2c.1**	Almost complete hindwing/s (≥75% complete)
			**2c.2**	Hindwing fragment/s
	**2d**	Isolated unassigned wing/s	**2d.1**	Almost complete wing/s (≥75% complete)
			**2d.2**	Wing fragment/s

**Indeterminable**	**??**	Unidentifiable insect fragments	**??**	Unidentifiable insect fragments

Systematic taxonomy

The below systematics for both entomofaunas are split into the most primitive insects (Ephemeroptera, Odonata, Dermaptera, Reculida, Blattodea, Orthoptera, and Hemiptera) and the holometabolous insects (Coleoptera, Necrotrichoptera, Mecoptera, Neuroptera, and Diptera), and follow standard entomological convention of the insect orders within these [e.g. 46]). Where there is no reference list the authority reference has been used.

Institutional abbreviations: **BGS,** British Geological Survey, Keyworth, UK; **BRSMG,** Bristol Museum & Art Gallery, Bristol, UK; **CAMSM,** Sedgwick Museum, Cambridge, UK; **CHAGM,** Wilson Museum, Cheltenham, UK; **NHMUK,** Natural History Museum, London, UK; **NMW,** National Museum Wales, Cardiff, UK; **PALE,** Museum of Comparative Zoology, Harvard University, USA; **TTNCM,** Somerset Heritage Centre, Taunton, UK; **WARMS,** Warwickshire Museum, Warwick, UK.

Alderton Hill entomofauna

Class Insecta Linnaeus 1758 [125]

Subclass Pterygota Brauer 1885 [128]

Order Ephemeroptera Hyatt & Arms, 1890 [129]

***Assigned material.* –** WARMS G 8084.

***Remarks.* –** Specimen was not available for re-examination (see materials and methods). WARMS G 8084 was originally reported as “*Ephemera*” by Kelly [114], however this

determination is uncertain, as only two undescribed ephemeropteran wings are known from the Toarcian insect-bearing localities of Grimmen and Dobbertin, Germany.

Order Odonata Fabricius, 1793 [130]

Suborder Anisozygoptera Handlirsch, 1906 [121]

Family Heterophlebiidae Needham, 1903 [131]

Genus *Heterophlebia* Westwood, 1849 [132]

Heterophlebia buckmani (Brodie, 1845) [63]

(Figure 3A)

***Holotype.* –** NHMUK PI I.11343, isolated partial forewing (33.8 mm long, 8.4 mm wide). ***Paratype.* –** NHMUK PI I.3988; previously holotype of *Heterophlebia dislocat*[illegible]issing from collections, unable to be re-examined).

***Assigned material.* –** NHMUK PI I.11310 & NHMUK PI I.11344, previously holotype of *Heterophlebia tillyar*[illegible]solated forewing, part and counterpart (33.8 mm long, 7.3 mm wide); BRSMG Cg2370 & BRSMG Cg2371, isolated partial forewing, part and counterpart (26 mm long, 8.4 mm wide); NHMUK PI I.59404 & NHMUK PI I.10496, isolated left hindwing, part and counterpart (35.8 mm long, 8.6 mm wide); NHMUK PI I.11312, previously holotype of *Heterophlebia angulat*[illegible]solated left partial male hindwing (20.7 mm long, 7.7 mm wide); NHMUK PI I.3317, two wing fragments (unable to be measured).

***Remarks.* –** All additional material possess venation identical to those of other specimens attributed to *Heterophlebia buckmani* [see 74].

***References.* –** [70, 74, 121, 133, 134].

Heterophlebia sp.

Assigned material. – CHAGM F.651, partial wing fragment (12.1 mm long, 8.1 mm wide); CHAGM F.654, wing tip fragment (12.3 mm long, 8.8 mm wide).

Family Liassogomphidae Tillyard 1935 [135]

Genus *Heterothemis* Handlirsch 1906 [121]

Heterothemis brodiei (Buckman 1843) [61]

(Figures 3B - 3C)

Holotype. – NHMUK PI I.3552, isolated left female hindwing, lectotype selected as holotype (45 mm long, 13.9 mm wide).

Assigned material. – BGS Geol.Soc.Coll. 4126, isolated forewing apex (19.6 mm long, 3.4 mm wide); NHMUK PI I.11288 & NHMUK PI I.11269, isolated partial right hindwing, part and counterpart (31.5 mm long, 11 mm wide); CHAGM F.653, isolated partial basal hindwing (21.3 mm long, 12.6 mm wide); CHAGM F.657.1 & CHAGM F.657.2, partial wing fragment, part and counterpart (27.8 mm long, 8.7 mm wide); WARMS G 8079 (unable to be measured).

Remarks. – WARMS G 8079 was not available for examination but was reported as *Heterothemis brodiei* by Kelly [114]. All additional material possess venation identical to those of other specimens attributed to *Heterothemis brodiei* [see 74].

References. – [59, 74, 121, 133, 136].

Heterothemis sp.

Assigned material. – CHAGM F.650, partial wing fragment (13.3 mm long, 11.7 mm wide); CHAGM F.655, basal wing fragment (20.9 mm long, 5 mm wide); CHAGM F.662, partial wing tip (13.6 mm long, 4.6 mm wide); PALE-8749, partial hindwing fragment (unable to be measured).

Remarks. – PALE-8749 was originally reported as ?*Liassogomphus* in the database of the Zoological Collections, Museum of Comparative Zoology, Harvard University.

Suborder Archizygoptera Handlirsch 1906 [121]

Family Protomyrmeleontidae Handlirsch 1906 [121]

Genus *Protomyrmeleon* Geinitz 1888 [118]

Protomyrmeleon sp.

(Figure 3D)

***Assigned material.* –** NHMUK PI In.59065, isolated partial supposed hindwing (6.8 mm long, 2.5 mm width).

***Remarks.* –** NHMUK PI In.59065 possess venation identical to those of other specimens attributed to *Protomyrmeleon* [see 74].

***References.* –** [137, 138].

Family ?Protomyrmeleontidae Handlirsch 1906 [121]

gen. et sp. indet

***Assigned material.* –** NHMUK PI I.11350, isolated partial wing (5.7 mm length, 2 mm width).

Family *Incertae sedis*

gen. et sp. indet

***Assigned material.* –** Isolated partial forewing: BRSMG Cg2359. Isolated partial wings: NHMUK PI I.3330; NHMUK PI In.59078 & NHMUK PI In.59080, part and counterpart; CHAGM F.630; CHAGM F.652; CHAGM F.681; NHMUK PI In.59075; NHMUK PI I.11347 & NHMUK PI I.11375 (part and counterpart); CAMSM TN 3193.

***Remarks.* –** Attributed to Odonata based on the dense cellular wing venation, but further taxonomic determination to family or genera level cannot be made due to fragmentary preservation.

Order ?Dermaptera De Geer 1773 [119]

Family *Incertae sedis*

(Figure 3E)

Assigned material. – NHMUK PI I.11403, isolated elytron (5.6 mm long, 3.5 mm wide).

Remarks. – Only tentatively assigned to this order due to poor preservation of the specimen.

Order Reculida Handlirsch 1906 [121]

Family Geinitziidae Handlirsch 1906 [121]

Genus *Geinitzia* Handlirsch 1906 [121]

Geinitzia carpentieri Zeuner 1937 [72]

(Figure 3F)

Holotype. – NHMUK PI In.36200, isolated partial forewing, part and counterpart (18.4 mm long, 7.2 mm wide).

Assigned material. – BRSMG Cg2364 & BRSMG Cg2365, isolated partial forewing, part and counterpart (9.8 mm length and 7.8 mm width).

Remarks. – Additional material attributed to *Geinitzia carpentieri* based on species characteristics described by Zeuner [72], including: MA_1 with terminal fork; CuA_2 not undulated.

References. – [47, 48, 72, 121, 133].

Superorder Dictyoptera Latreille 1829 [139]

Order Blattodea Latreille 1810 [120]

Family ?Raphidiomimidae Vishnyakova 1973 [140]

Genus *Liadoblattina* Handlirsch, 1906 [121]

Liadoblattina blakei (Scudder, 1886) [68]

(Figure 3G)

***Holotype.* –** NHMUK PI I.3574, isolated tegmin (14.6 mm long, 5 mm wide).

***Assigned material.* –** NHMUK PI I.11361, tegmin and pronotum (2.5 mm long, 1.5 mm wide); NHMUK PI I.3562, previously holotype of *Mesoblattina bensoni,* isolated tegmin (18 mm long, 5 mm wide); NHMUK PI I.11341, isolated partial tegmin, part and counterpart (16.1 mm long, 5 mm wide).

***Remarks.* –** Attributed to *Liadoblattina blakei* based on species characteristics described by Vršanský and Ansorge [76], including an elongate tegmen, that is up to three times as long (*c.* 20 mm) as it is wide, and that the clavus is more than twice as long as it is wide.

***References.* –** [68, 76, 121, 133].

Family Caloblattinidae Vršanský and Ansorge, 2000 [141]

gen. *et* sp. nov.

(Figure 3H)

***Assigned material.* –** BRSMG Cg2374 & BRSMG Cg2375, isolated tegmin, part and counterpart (12.8 mm long, 3.8 mm wide).

***Remarks.* –** The specimen can be tentatively assigned to the family Caloblattinidae, based on the presence of branched anal veins in the clavus [141]; however this family is in much need of taxonomic revision. This specimen is noticeably dissimilar to other known Toarcian cockroaches and represents a new genus and species of cockroach.

Family *Incertae sedis*

gen. et sp. indet

***Assigned material.* –** CHAGM F.660, isolated partial wing (6.8 mm long, 2 mm wide); NHMUK PI I.70, wing fragment (3.1 mm long, 2 mm wide).

***Remarks.* –** Attributed to Blattodea based on the tegmin venation, which involves numerous closely spaced parallel veins, however further taxonomic determination to family or genera level cannot be made due to poor venation preservation.

Order Orthoptera Olivier 1789 [123]

Family Locustopsidae Handlirsch 1906 [121]

Genus *Locustopsis* Handlirsch 1906 [121]

Locustopsis sp.

(Figure 3I)

***Assigned material.* –** Isolated forewings: NHMUK PI I.11303; NHMUK PI I.11370. Isolated partial forewings: NHMUK PI I.11334; NHMUK PI I.11330; NHMUK PI I.11363; NHMUK PI I.11351. Isolated hindwing: NHMUK PI I.11340.

***Remarks.* –** Specimens possess venation and colour patterns identical to those of other specimens attributed to the genus *Locustopsis* [see 74].

***References.* –** [121, 133, 142].

Family Locustopsidae Handlirsch 1906 [121]

gen. et sp. indet

***Assigned material.* –** BGS GSM 117360, isolated partial wing (8.7 mm long, 2.5 mm wide).

Family Elcanidae Handlirsch 1906 [121]

Genus "*Elcana*" Giebel 1856 [143]

***Remarks.* –** Since Gorochov *et al.* [144] synonymized *Elcana* Giebel, 1856 [143] with *Panorpidium* Westwood, 1854 [66], *Elcana* is no longer available as a generic name. Both generic names were based on the same specimen, the type species of *P. tesselatum* Westwood, 1854. Gorochov *et al.* [144] divided Elcanidae in two subfamilies: Elcaninae Handlirsch, 1906 [121] [= Baisselcaninae Gorochov, 1986] and Archelcaninae Gorochov, Jarzembowski & Coram, 2006 [144].

The numerous species of Archelcaninae included in *Elcana* by earlier authors [see 121, 145, 146] are in need of taxonomic revision [144]. Although a possible generic name is *Clathrotermes* Heer, 1865 [147] which is used for Early Jurassic elcanids, we prefer to use the name "*Elcana*" until a revision is undertaken. At species level, we follow Zessin [146] in his treatment of Early Toarcian elcanid species, who reduced more than 100 Lower Toarcian species described by Handlirsch [121, 133] and Bode [47, 48] from Dobbertin and the vicinity of Braunschweig to four: "*Elcana*" *minima* Handlirsch, 1906 [121], "*Elcana*"*geinitzi* (Heer, 1880) [122], "*Elcana*" *media* Handlirsch, 1906 [121] and "*Elcana*" *magna* Handlirsch, 1906 [121]. Recently, Heads *et al.* [148] described two new elcanid species from the Lower Toarcian of Luxembourg as *Archelcana tina* and *A. numbergerae*. However, these species are synonyms of "*Elcana*" *geinitzi* (*tina*) and "*Elcana" media* (*numbergerae).*

"*Elcana" cf. geinitzi* (Heer, 1880) [122]

(Figures 3J - 3K)

***Holotype.* –** MBI 15.1 a,b, isolated forewing. Grimmen Formation, 'Green Series'; Early Jurassic: Lower Toarcian of Dobbertin, Mecklenburg-Vorpommern, Germany.

***Assigned material.* –** NHMUK PI I.11427, isolated partial forewing (8.1 mm long, 2.1 mm wide); NHMUK PI I.11295, isolated partial forewing (10 mm long, 2.8 mm wide); NHMUK PI I.3349 (specimen was not available to be measured); NHMUK PI I.3560, previously holotype of *Elcana brodiei* (Handlirsch 1906) [121], isolated partial forewing (7.2 mm long, 2.6 mm wide).

Remarks. – NHMUK PI I.3349 was reported as *Archelcana geinitzi* by Kelly [114].

References. – [48, 121, 124, 133, 146, 149].

"Elcana" sp.

Assigned material. – NHMUK PI I.11394, isolated partial forewing tip (5.1 mm long, 2 mm wide); NHMUK PI I.38, part and counterpart (9.2 mm long, 4.5 mm wide)

Family Protogryllidae Zeuner 1937 [72]

Genus *Protogryllus* Handlirsch 1906 [121]

Protogryllus cf. dobbertinensis Geinitz (1880) [124]

Holotype. – FGWG 117/7, isolated forewing. Grimmen Formation, 'Green Series'; Early Jurassic: Lower Toarcian of Dobbertin, Mecklenburg-Vorpommern, Germany.

Assigned material. – NHMUK PI I.11421, isolated partial male forewing (16.4 mm long, 5.3 mm wide).

References. – [46, 48, 72, 121, 133].

Protogryllus magnus Zeuner 1937 [72]

(Figure 3L)

Holotype. – NHMUK PI I.11324, isolated partial male forewing (27 mm long, 5.7 mm wide).

References. – [72].

Protogryllus sp.

Assigned material. – NHMUK PI I.11377, partial body with wings (17.7 mm long, 2.7 mm wide); NHMUK PI I.11461, isolated partial wing (12.3 mm long and 3.7 wide).

Family *Incertae sedis*

gen. et sp. indet

(Figure 3M)

***Assigned material.* –** Almost complete individuals: NHMUK PI I.11270. Isolated exoskeletal elements: NHMUK PI I.3314; NHMUK PI I.11321; NHMUK PI I.11405; NHMUK PI I.11333; NHMUK PI I.11392. Isolated partial wings: BGS GSM 117359; BGS GSM 117361; NHMUK PI I.3305; NHMUK PI I.11301; NHMUK PI I.11290; NHMUK PI I.11396; NHMUK PI I.3304; NHMUK PI I.11264. Indeterminable: NHMUK PI I.11342; NHMUK PI I.11332; NHMUK PI I.59084.

***Remarks.* –** Attributed to Orthoptera based on the tegminised forewings and distinctive colouration patterns. Further taxonomic determination to family or genera level cannot be made due to fragmentary wing preservation.

Order Hemiptera Linnaeus 1758 [125]

Suborder Auchenorrhyncha Duméril 1805 [150]

Family Fulgoridiidae Handlirsch 1939 [133]

Genus *Fulgoridium* Handlirsch 1906 [121]

Fulgoridium sp.

(Figure 4A)

***Assigned material.* –** Isolated skeletal elements: NHMUK PI I.11424. Isolated forewings: NHMUK PI I.3331 & NHMUK PI I.3334, part and counterpart; NHMUK PI I.11328. Isolated partial forewing: NHMUK PI I.3079. Isolated partial wings: NHMUK PI I.11275; NHMUK PI I.11293; NHMUK PI I.11315. Indeterminable: NHMUK PI I.11278; NHMUK PI I.11453.

***Remarks.* –** Specimens NHMUK PI I.11278 and NHMUK PI I.11453 were not available for re-examination but were reported as *Fulgoridium* by Kelly [114]. Until a revision of all species of *Fulgoridium* and the related genera and subgenera is undertaken, it is impossible to determine the Alderton Hill specimens more precisely. Only the genera *Fulgoridulum* Handlirsch 1939 [133], *Margaroptilon* Handlirsch 1906 [121], and *Elasmoscelidium* Martynov 1926 [151] are currently well defined.

***References.* –** [46, 48, 121, 133, 151].

Genus *Margaroptilon* Handlirsch 1906 [121]

Margaroptilon brodiei Handlirsch 1906 [121]

(Figures 4B - 4C)

1906 *Margaroptilon brodiei* Handlirsch, p. 499

1906 *Margaroptilon woodwardi* Handlirsch, p. 499 syn. nov.

1906 *Margaroptilon bulleni* Handlirsch, p. 499 syn. nov.

***Holotype.* –** NHMUK PI I.3561, spotted isolated partial forewing (6 mm long, 2.1 mm wide); NHMUK PI I.11266 (previo[illegible]y holotype of *M. bulleni*) & NHMUK PI I.11286 (previously holotype of *M. woodwardi*), isolated forewing, part and counterpart (6.3 mm long, 2.5 mm wide).

***Assigned material.* –** Isolated forewings: CHAGM F.642; CHAGM F.664; NHMUK PI I.11296. Isolated partial forewings: CHAGM F.659.1 & CHAGM F.659.2 (part and counterpart); CHAGM F.661; CHAGM F.635; NHMUK PI I.11374; NHMUK PI I.11399; NHMUK PI I.11414; NHMUK PI I.11426; NHMUK PI I.11429; NHMUK PI I.15015; NHMUK PI I.3341; NHMUK PI I.11268; NHMUK PI I.11415. Indeterminable: NHMUK PI I.3309.

***Remarks.* –** Kelly [114[illegible]synonymized *Margaroptilon brodiei* Handlirsch, 1908 [121]; *Margaroptilon bulleni* Handlirsch (1908) [121]; and *Margaroptilon woodwardi* Handlirsch, 1908 [121] based on morphological similarity, with the latter being the senior synonym. He

recognized that *H. woodwardi* and *M. bulleni* were based on part and counterpart of the same wing. We regard the small differences in spot arrangement between *M. woodwardi* and *M. brodiei* as intraspecific variation, as Kelly [114] did. However, the name *M. brodiei* has priority instead of *M. woodwardi.* NHMUK PI I.3309 was not available for re-examination but was reported as *Margaroptilon woodwardi* by Kelly [114]. *Margaroptilon* is a planthopper with faint venation typical for fulgoromorphans, although it is difficult to study as the well sclerotized wing spots resemble those of *Fulgoridulum*.

***References.* –** [48, 114, 121].

Family Fulgoridiidae Handlirsch 1939 [133]

gen. et sp. indet.

***Assigned material.* –** CHAGM F.636, isolated partial wing tip (7 mm long, 3.1 mm wide); CAMSM TN 3195, partial spotted wing (5.6 mm long, 2.7 mm wide).

Family Archijassidae Becker-Migdisova 1962 [152]

Genus *Archijassus* Handlirsch 1906 [121]

Archijassus heeri (Geinitz 1880) [124]

(Figure 4D)

***Assigned material.* –** NHMUK PI I.11458, isolated forewing (specimen not available for re-examination).

***References.* –** [46, 124].

Suborder Coleorrhyncha Myers and China 1929 [153]

Family Progonocimicidae Handlirsch 1906 [121]

gen. et sp. indet.

(Figure 4E)

Assigned material. – NHMUK PI I.11274, isolated partial body (3.3 mm long, 1.1 mm wide); NHMUK PI I.11465, isolated forewing (4 mm long).

Suborder Heteroptera Latreille 1810 [120]

Infraorder Gerromorpha Popov 1971 [154]

Genus *Engynabis* Bode 1953 [48]

Engynabis tenuis Bode 1953 [48]

(Figure 4F)

Assigned material. – NHMUK PI I.3313, isolated elongated body with paired wings (5.3 mm long, 1.2 mm wide).

References. – [48, 155].

Family *Incertae sedis*

gen. et sp. indet.

(Figures 4G - 4I)

Assigned material. – Partial individuals: NHMUK PI I.11362; NHMUK PI I.11446; CHAGM F.643.1 & CHAGM F.643.2. Isolated bodies: NHMUK PI I.11280; NHMUK PI I.11294; NHMUK PI I.11297; NHMUK PI I.11317; NHMUK PI I.82 & NHMUK PI I.74 (part and counterpart); NHMUK PI I.11379. Isolated skeletal elements: NHMUK PI I.11454; NHMUK PI I.3052; NHMUK PI I.11285; NHMUK PI I.11456; NHMUK PI I.11452; NHMUK PI I.3324; NHMUK PI I.68; NHMUK PI I.11445; BGS GSM 117358; CHAGM F.634.1 & CHAGM F.634.2 (part and counterpart). Isolated forewing: NHMUK PI I.78. Isolated partial hindwings: NHMUK PI I.11439. Isolated partial wings: NHMUK PI I.3342; CHAGM F.671 & CHAGM F.672 (part and counterpart); CHAGM F.670.1 & CHAGM F.670.2 (part and counterpart). Indeterminate: NHMUK PI In. 59079.

Order Coleoptera Linnaeus 1758 [125]

gen. et sp. indet.

(Figures 4J - 4M)

Assigned material. – Almost complete individuals: CHAGM F.645; CHAGM F.646; NHMUK PI I.11339; NHMUK PI I.11360. Partial individuals: CHAGM F.638; CHAGM F.647.1 & CHAGM F.647.2; CHAGM F.649.1 & CHAGM F.649.2; NHMUK PI I.3307; NHMUK PI I.11345; NHMUK PI I.11383; CAMSM TN 3196 a+b. Isolated bodies: NHMUK PI I.11267; NHMUK PI I.11287; NHMUK PI I.11292. Isolated skeletal elements: NHMUK PI I.69; NHMUK PI I.3308; NHMUK PI I.11302; NHMUK PI I.11404; NHMUK PI I.81. Isolated forewings: CHAGM F.640; CHAGM F.644; CHAGM F.663; CHAGM F.667; NHMUK PI I.3052; NHMUK PI I.3077; NHMUK PI I.3078; NHMUK PI I.11318; NHMUK PI I.11331; NHMUK PI I.11353 & NHMUK PI I.11354; NHMUK PI I.11355 & NHMUK PI I.11376; NHMUK PI I.11359; NHMUK PI I.11369; NHMUK PI I.11411; NHMUK PI I.11420; NHMUK PI I.11431; NHMUK PI I.11432; NHMUK PI I.11438; NHMUK PI I.11441; NHMUK PI I.59071. Isolated partial forewings: CHAGM F.639; CHAGM F.641; NHMUK PI I.3335; NHMUK PI I.11368. Indeterminable: NHMUK PI I.2163; NHMUK PI I.3339; NHMUK PI I.3348 & NHMUK PI I.11308 (part and counterpart); NHMUK PI In. 59072; NHMUK PI In.59286; NHMUK PI In.59285; NHMUK PI In.59287; NHMUK PI In.59289; NHMUK PI In.59294; NHMUK PI I.11381.

Remarks. – Attributed to Coleoptera based on the forewings which are modified into highly sclerotised elytra. However, the majority of the Coleoptera is represented by disarticulated and fragmented elytra which are compressed, poorly preserved, and therefore difficult to identify beyond order level. Therefore, all beetles specimens from Alderton Hill remain unassigned, and no types are based on the recorded specimens. However, at least three elytra morphotypes can be identified, including smooth (NHMUK PI I.11345; Fig 4K), striated (NHMUK PI I.3077; Fig 4L) and pitted (NHMUK PI I.11355 and NHMUK PI I.11376; Fig 4M),

indicating that the beetle fauna is likely to consist of several genera or species. The thoracic fragment NHMUK PI I.3052 was originally assigned to the recent genus *Coccinella* Linnaeus 1758 [125] based on the associated specimen label, however this identification cannot be supported.

Order Necrotrichoptera Engel 2022 [156]

Family Necrotauliidae Handlirsch 1906 [121]

Genus *Necrotaulius* Handlirsch 1906 [121]

Necrotaulius parvulus (Geinitz 1884) [67]

(Figures 4N - 4O)

Holotype. – FGWG 119/7, isolated wing. Grimmen Formation, 'Green Series'; Early Jurassic: Lower Toarcian of Dobbertin, Mecklenburg-Vorpommern, Germany.

Assigned material. – NHMUK PI I.15016 & NHMUK PI I.15014 (part and counterpart) isolated forewing, previously holotype of *Necrotaulius pygmaeus* Tillyard 1933 [71] (3.2 mm long, 0.9 mm wide); NHMUK PI I.11425, isolated forewing (2.8 mm long, 0.7 mm wide); NHMUK PI I.3336, isolated body (3.8 mm long, 1.8 mm wide); NHMUK PI I.11389 (specimen not available for re-examination).

Remarks. – *Necrotaulius parvulus* is distinct due to the dense coverage of the wing with hairs. Wing venation of this species is similar to Trichoptera, however it has no distally bent CuP, a synapomorphic character of Trichoptera [78, 157]. Because of this character, we here exclude *Austaulius* Thomson, Ross and Coram 2018 [45] from Necrotauliidae and transfer it to Trichoptera *incertae familiae.*

References. – [67, 78, 114, 121, 133, 157, 158, 159].

?*Necrotaulius* sp.

***Assigned material.* –** CHAGM F.637, isolated partial wing (specimen unable to be measured).

Order Mecoptera Packard 1886 [160]

Family Bittacidae Handlirsch 1906 [121]

Genus *Protobittacus* Tillyard 1933 [71]

Protobittacus handlirschi Tillyard 1933 [71]

(Figure 4P)

***Holotype.* –** NHMUK PI I.11406 & NHMUK PI I.11335, isolated hindwing, part and counterpart (14.4 mm long, 3.7 mm wide).

Remarks. During our investigation of the Alderton Hill insect material, we located the previously unknown counterpart of the holotype (NHMUK PI I.11335), which displays most of the proximal part of the wing including the unique type of A1/A2 fork (Fig 4P).

***References.* –** [71].

Bittacidae gen. et sp. indet.

***Assigned material.* –** CHAGM F.632, isolated wing (8.5 mm long, 2 mm long).

Family Orthophlebiidae Handlirsch 1906 [121]

Genus *Orthophlebia* Westwood 1845 [115]

Orthophlebia brodiei Tillyard 1933 [71]

(Figure 4Q)

***Holotype.* –** NHMUK PI I.15019, isolated forewing (10.7 mm long, 3 mm wide).

***Remarks.* –** Note that Tillyard 1933 [71] previously referred to the holotype specimen as NHMUK PI I.15017; see Tillyard [71] for the original holotype description.

***References.* –** [46, 71, 161, 162].

Orthophlebia sp.

(Figure 4R)

***Assigned material.* –** Isolated forewings: NHMUK PI I.11413; NHMUK PI I.11418 & NHMUK PI I.11395 (part and counterpart); NHMUK PI I.11225; NHMUK PI I.11387. Isolated partial forewing: NHMUK PI I.11271. Isolated hindwing: NHMUK PI I.15013. Isolated partial hindwing: NHMUK PI I.11366. Isolated wing: NHMUK PI I.11448. Isolated partial wing: CAMSM TN 3192.

***References.* –** [46, 115, 161, 162].

Family Pseudopolycentropodidae Handlirsch 1920 [163]

Genus *Pseudopolycentropus* Handlirsch 1906 [121]

Pseudopolycentropus sp.

***Assigned material.* –** CHAGM F.656, isolated forewing (5.4 mm long, 2 mm wide).

***References.* –** [46, 48, 121, 133, 164].

Order Neuroptera Linnaeus 1758 [125]

Family Osmylopsychopidae Martynova 1949 [161]

Genus *Actinophlebia* Handlirsch 1906 [121]

Actinophlebia intermixta (Scudder 1885) [165]

(Figures 5A - 5B)

***Holotype.* –** NHMUK PI I.3577 & NHMUK PI I.11346, isolated forewing, part and counterpart (9.2 mm length, 5.5 mm width). *Actinophlebia anglicana* Tillyard 1933 [71] (NHMUK PI I.11346) is as counterpart of *Actinophlebia intermixta* (Scudder 1885) [165], an objective synonym of the latter.

***Assigned material.* –** NHMUK PI I.11349, isolated forewing (6.3 mm length and 4.1 mm width); NHMUK PI I.11319, isolated partial forewing (7.3 mm length and 4.5 mm width).

***Remarks.* –** Neuropterans from the Toarcian with a triangular wing shape are often attributed to the genus *Actinophlebia* [46, 69, 71, 121]. However, Ponomarenko [166] regarded similar wings with a distally fused Sc with RA as belonging to *Parhemerobius* Bode 1953 [48]. In *Actinophlebia*, it was thought that the Sc is running free into the costal margin. This, however, is most likely based on a misinterpretation of the course of Sc, as this featured can be observed in the *Actinophlebia* sp. specimen LGA 1080 from the Lower Toarcian of Grimmen, Germany [see 46]. Note that Tillyard [71] originally measured NHMUK PI I.11346 at 11.2 mmm long, while Scudder [165] measured a length of 10 mm.

***References.* –** [46, 48, 69, 71, 121, 133, 165, 166, 167, 168, 169].

Actinophlebia sp.

***Assigned material.* –** NHMUK PI I.11314, isolated partial wing (6.8 mm length, 4.4 mm width).

"Familia nova A" Makarkin et al., 2012 [170]

Genus *Paractinophlebia* Handlirsch 1906 [121]

Paractinophlebia curtisii (Scudder 1886) [68]

(Figure 5C)

***Holotype.* –** NHMUK PI I.3585, isolated partial hindwing (27.1 mm long, 7.9 mm wide); the counterpart (PALE-8751) is housed in the Entomology collection at the Museum of Comparative Zoology, Harvard University, and previously assigned as Neuroptera sp.

Assigned material. – NHMUK PI I.11320, isolated partial wing fragment (17.9 mm long, 9 mm wide).

Remarks. – Scudder [68] figured the counterpart (plate 48, figure 16) at PALE. The holotype is most likely a hindwing fragment, which is strongly dissimilar to the wings of Kalligrammatidae [see 69], in particular by the presence of rare cross-veins arranged in series and by the very narrow subcostal space [96].

References. – [68, 96, 170].

Family "Prohemerobiidae" Handlirsch 1906 [121]

Genus *"Prohemerobius"* Handlirsch 1906 [121]

"Prohemerobius" aldertonensis Whalley 1988 [69]

(Figure 5D)

Holotype. – NHMUK PI I.11304, isolated partial forewing (4 mm long, 1.5 mm wide).

Assigned material. – NHMUK PI I.11279, isolated partial wing (5.7 mm long, 2.7 mm wide); NHMUK PI I.11390, isolated partial wing (6.6 mm long, 1.8 mm wide).

Remarks. – The genus *Prohemerobius* Handlirsch 1906 [121] is regarded as a waste basket taxon for small Lower Jurassic neuropterans, and therefore requires a complete revision. Ponomarenko 1995 [166] regards the English Lower Toarcian species *Archeosmylus alysius* Whalley 1988 [69] and *A. complexus* Whalley 1988 [69] as belonging to *Prohemerobius*. The only difference is whether the Sc and RA run parallel or strongly converge. We refer here the assignment of specimen to the certain taxa as provided by Whalley [69].

References. – [69, 166, 171].

"Prohemerobius" sp.

***Assigned material.* –** NHMUK PI I.11284, isolated wing (7.4 mm length, 3.3 mm width); NHMUK PI Ig 60, isolated wing (9.1 mm length, 2.4 mm width); NHMUK PI I.11417, partial individual with wings (specimen was not available for measurement).

“Archeosmylus” alysius Whalley 1988 [69]

(Figure 5E)

***Holotype.* –** NHMUK PI I.3318, body (2.3 mm long, 0.5 mm wide) with attached wings (4.3 mm long, 1.7 mm wide), part and counterpart.

***Paratypes.* –** NHMUK PI I.11416, isolated wing (5 mm long, 2.5 mm wide); NHMUK PI I.11433, partial individual with paired wings (3.5 mm long, 1.9 mm wide).

***Assigned material.* –** NHMUK PI I.11281, isolated wing (3.7 mm long, 1.8 mm wide); NHMUK PI I.11402, isolated partial wing (6.5 mm long, 1.7 mm wide); NHMUK PI I.11347 & NHMUK PI I.11375, partial individual, part and counterpart (specimen could not be measured).

***References.* –** [69, 166].

“Archeosmylus” complexus Whalley 1988 [69]

(Figure 5F)

***Holotype.* –** NHMUK PI I.11412, isolated wing (6.7 mm long, 3.7 mm wide).

***Paratype.* –** NHMUK PI I.3315 & NHMUK PI I.3380, isolated wing, part and counterpart (9.1 mm, 2.1 mm wide).

***References.* –** [69, 166].

Family “Prohemerobiidae“ Handlirsch 1906 [121]

gen. et sp. indet.

***Assigned material.* –** BRSMG Cd 1397, isolated partial wing (9 mm long, 4.7 mm wide).

Family *Incertae sedis*

gen. et sp. indet.

Assigned material. – Partial individual: NHMUK PI I.11409. Isolated wings: NHMUK PI I.66 & NHMUK PI I.66-83, part and counterpart; NHMUK PI I.11385. Isolated partial wings: NHMUK PI I.3337; NHMUK PI I.11326; NHMUK PI I.11401; NHMUK PI I.11463; NHMUK PI I.11464. Indeterminable: WARMS G 8087; NHMUK PI I.11434.

Remarks. – WARMS G 8087 was not available for re-examination but was reported as Neuroptera by Kelly [114]. See supplementary material for individual measurements and descriptions.

Order Diptera Linnaeus, 1758 [125]

Suborder Nematocera Latreille, 1825 [172]

Family Limoniidae Speiser, 1909 [173]

Genus *Architipula* Handlirsch, 1906 [121]

Architipula anglicana (Tillyard, 1933) [71]

(Figure 5G)

Holotype. – NHMUK PI I.11298, isolated wing (8.2 mm long, 2.2 mm wide).

Assigned material. – NHMUK PI I.11272, isolated wing (5.9 mm long, 2 mm wide); NHMUK PI I.11365, isolated wing (7.3 mm long, 2.5 mm wide); NHMUK PI I.11313, isolated partial wing (6.5 mm long, 2.6 mm wide); NHMUK PI I.11398, isolated partial wing (8.7 mm long, 1.3 mm wide).

Remarks. – Note that NHMUK PI I.11298 was formerly described within *Liassotipula* Tillyard, 1933 [71], which was then synonymized with *Architipula* by Kopeć et al. [77]. Venation of additional material is identical to other specimens attributed to *Architipula anglicana* as described by Kopeć et al. [77].

***References.* –** [71, 77, 121].

Architipula sp.

***Assigned material.* –** CHAGM F.633, isolated wing (6.2 mm long, 1.7 mm wide).

Genus *Grimmenia* Krzemiński and Zessin 1990 [174]

Grimmenia tillyardi Kopeć et al. 2017b [77]

(Figure 5H)

***Holotype.* –** NHMUK PI I.3328, isolated wing (3.8 mm long, 0.9 mm wide).

***References.* –** [77].

Genus *Mesotipula* Handlirsch 1920 [163]

Mesotipula slatteri Kopeć et al. 2018 [175]

***Holotype.* –** NHMUK PI I.11316 & NHMUK PI I.11311, isolated female imago, part and counterpart (5.7 mm long, 1.9 mm wide).

***Assigned material.* –** NHMUK PI I.3320 & NHMUK PI I.3323, isolated partial wing, part and counterpart (6.4 mm long, 1.7 mm wide).

***Remarks.* –** Venation of additional material is identical to other specimens attributed to *Mesotipula slatteri* as described by Kopeć et al. [175].

***References.* –** [175].

Family Limoniidae Speiser 1909 [173]

gen. et sp. indet

***Assigned material.* –** Partial individual: NHMUK PI I.11329. Isolated skeletal elements: NHMUK PI I.71; NHMUK PI I.11328; NHMUK PI I.11348; NHMUK PI I.11380; NHMUK PI I.11391; NHMUK PI I.3319; NHMUK PI I.3345; NHMUK PI I.3350. Isolated partial wings: NHMUK PI In.59069; NHMUK PI In.59074; NHMUK PI In.59076.

Family *Incertae sedis*

gen. et sp. indet

***Assigned material.* –** NHMUK PI I.11276, isolated segmented body (5.3 mm long, 0.7 mm wide). Isolated partial wings: NHMUK PI I.73 (3.7 mm long, 1 mm wide); NHMUK PI I.11315 (7.9 mm long, 2.2 mm wide); NHMUK PI I.11358 (7.8 mm long, 0.5 mm wide).

Strawberry Bank entomofauna

Class Insecta Linnaeus 1758 [125]

Subclass Pterygota Brauer 1885 [128]

Order Odonata Fabricius 1793 [130]

Suborder Anisozygoptera

Family Liassogomphidae Tillyard 1935 [135]

gen. et sp. indet.

***Assigned material.* –** TTNCM 39/2011/0364, isolated wing fragment (10.7 mm long, 8 mm wide); TTNCM 39/2011/0509, isolated wing fragment (26.9 mm long, 7.9 mm wide).

***Remarks.* –** TTNCM 39/2011/0509 is figured in Williams *et al.* [43] (Fig 3C).

***References.* –** [43, 74].

Family *Incertae sedis*

gen. et sp. indet.

***Assigned material.* –** TTNCM 39/2011/0362a-b; TTNCM 39/2011/0403a-b; TTNCM 39/2011/0408; TTNCM 39/2011/0420a-b; TTNCM 39/2011/0503 & TTNCM 39/2011/0504 (part and counterpart); TTNCM 39/2011/0505; TTNCM 39/2011/0506; TTNCM 39/2011/0507a-b; TTNCM 39/2011/0511; TTNCM 39/2011/0512; TTNCM 39/2011/0514; TTNCM 39/2011/0515; TTNCM 39/2011/0516; TTNCM 39/2011/0517; TTNCM 39/2011/0528; TTNCM 39/2011/0545; TTNCM 39/2011/1013; TTNCM 39/2011/1014.

Superorder Dictyoptera Latreille 1829 [139]

Order Blattodea Latreille 1810 [120]

Family Caloblattinidae Vršanský and Ansorge 2000 [141]

Genus *Rhipidoblattina* Handlirsch 1906 [121]

Rhipidoblattina sp.

***Assigned material.* –** TTNCM 39/2011/0537 & TTNCM 39/2011/0538, isolated forewing, part and counterpart (8.4 mm long, 2.8 mm wide).

***References.* –** [141].

Family *Incertae sedis*

gen. et sp. indet

***Assigned material.* –** Isolated partial forewing: TTNCM 39/2011/1008. Isolated partial wings: TTNCM 39/2011/0391; TTNCM 39/2011/0392 & TTNCM 39/2011/0543 (part and counterpart); TTNCM 39/2011/0393 & TTNCM 39/2011/0395 (part and counterpart); TTNCM 39/2011/0396; TTNCM 39/2011/0398; TTNCM 39/2011/0405; TTNCM 39/2011/0410; plus an additional specimen with no accession number.

Order Dermaptera De Geer 1773 [119]

Family Dermapteridae Vishnyakova 1980 [176]

Genus *Trivenapteron* Kelly *et al.* 2016 [44]

Trivenapteron moorei Kelly *et al.* 2016 [44]

***Holotype.* –** TTNCM 39/2011/0489, isolated sub-quadrate, truncate tegmen, pointed apically and without ornamentation.

***References.* –** [43, 44].

Order Orthoptera Olivier 1789 [123]

Family Locustopsidae Handlirsch 1906 [121]

Genus *Locustopsis* Handlirsch 1906 [121]

Locustopsis sp.

Assigned material.* –** NHMUK PI I.11485,*** isolated hindwing fragment (14 mm long, 2.4 mm wide).

***References.* –** [121, 133, 142].

Family Locustopsidae Handlirsch 1906 [121]

gen. et sp. indet.

***Assigned material.* –** TTNCM 39/2011/0520 & TTNCM 39/2011/0522, isolated wing fragment, part and counterpart fragment (16 mm long, 3.8 mm wide); TTNCM 39/2011/0527, isolated forewing fragment (16.5 mm long, 4.2 mm wide); TTNCM 39/2011/0731, isolated wing (13 mm long, 2.6 mm wide); CAMSM TN 3200, partial wing (7.2 mm long, 2.8 mm wide).

Family Elcanidae Handlirsch 1906 [121]

gen. et sp. indet.

***Assigned material.* –** Isolated forewing: TTNCM 39/2011/0523**.** Isolated partial forewing: TTNCM 39/2011/0424a-b; TTNCM 39/2011/0525**.** Isolated wing: TTNCM 39/2011/0544**.** Isolated partial wing: TTNCM 39/2011/0732; TTNCM 39/2011/0411; TTNCM 39/2011/0397; TTNCM 39/2011/0419; TTNCM 39/2011/0434; TTNCM 39/2011/0415. Indeterminable: TTNCM 39/2011/0530; TTNCM 39/2011/0533; TTNCM 39/2011/0486a-b.

***Remarks.* –** See supplementary material for individual measurements and descriptions. TTNCM 39/2011/0523 is figured in Williams *et al.* [43] (Fig 3D).

***References.* –** [43, 121, 133].

Family Protogryllidae Zeuner, 1937 [72]

gen. et sp. indet.

***Assigned material.* –** TTNCM 39/2011/1006

***Remarks.* –** Specimen was not available to be re-examined. TTNCM 39/2011/1006 was originally reported as belonging to Protogryllidae by Williams *et al.* [43].

Family *Incertae sedis*

gen. et sp. indet.

***Assigned material.* –** NHMUK PI I.11490; TTNCM 39/2011/0389; TTNCM 39/2011/0401; TTNCM 39/2011/0409; TTNCM 39/2011/0421; TTNCM 39/2011/0521; TTNCM 39/2011/0524; TTNCM 39/2011/0526; TTNCM 39/2011/0529; TTNCM 39/2011/0531; TTNCM 39/2011/0532a-b; TTNCM 39/2011/0534; TTNCM 39/2011/0552; TTNCM 39/2011/1007.

***Remarks.* –** See supplementary material for individual measurements and descriptions.

Order Hemiptera Linnaeus 1758 [125]

Suborder Auchenorrhyncha Duméril 1805 [150]

Family Fulgoridiidae Handlirsch 1939 [133]

Genus *Margaroptilon* Handlirsch 1906 [121]

Margaroptilon brodiei Handlirsch 1906 [121]

***Holotype.* –** NHMUK PI I.3561, isolated forewing. Whitby Mudstone Formation: Dumbleton Member; Early Jurassic: Lower Toarcian of Alderton Hill, Gloucestershire, UK.

***Assigned material.* –** TTNCM 39/2011/0593, isolated forewing (5 mm long, 2.4 mm wide); NHMUK PI I.11492, isolated forewing (unable to be measured).

***Remarks.* –** See Alderton Hill remarks under *Margaroptilon* for discussion on *M. brodiei*.

***References.* –** [48, 114, 121].

Margaroptilon sp.

***Assigned material.* –** Isolated spotted wings: TTNCM 39/2011/0535 (8 mm long, 2.2 mm wide); TTNCM 39/2011/0592 (6.6 mm long, 2 mm wide); TTNCM 39/2011/0594 (5 mm long, 2 mm wide); TTNCM 39/2011/0595 (7.8 mm long, 2.2 mm wide); Isolated spotted wing fragment: TTNCM 39/2011/0995.

***Remarks.* –** Williams *et al.* [43] identified and figured TTNCM 39/2011/0594 as *Fulgoridulum* sp. (Fig 3A), however here we attribute these specimens to *Margaroptilon* sp.

Suborder Heteroptera Latreille 1810 [120]

Family Archegocimicidae Handlirsch 1906 [121]

***Assigned material.* –** TTNCM 39/2011/0707a-b, isolated partial forewing (3.2 mm long, 2.2 mm wide); TTNCM 39/2011/0394, isolated wing (3.1 mm long, 1 mm wide); TTNCM 39/2011/0549, isolated wing (3.6 mm long, 1.4 mm wide).

***Remarks.* –** TTNCM 39/2011/0707a-b is figured in Williams *et al.* [43] (Fig 3E).

Family *Incertae sedis*

gen. et sp. indet.

***Assigned material.* –** Almost complete individuals: TTNCM 39/2011/0692; TTNCM 39/2011/0694; TTNCM 39/2011/0689a-b. Partial individual: TTNCM 39/2011/0665; TTNCM 39/2011/0693; TTNCM 39/2011/0696; NHMUK PI I.11493. Isolated bodies: TTNCM 39/2011/0646; TTNCM 39/2011/0648. Isolated skeletal elements: TTNCM 39/2011/0440; TTNCM 39/2011/0563a-b; TTNCM 39/2011/0570; TTNCM 39/2011/0647. Isolated partial forewings: TTNCM 39/2011/0700a-b; NHMUK PI I.11489. Isolated wing: TTNCM 39/2011/0416; TTNCM 39/2011/0429; TTNCM 39/2011/0480a-b; TTNCM 39/2011/0547; TTNCM 39/2011/0572; TTNCM 39/2011/0540a-b; TTNCM 39/2011/0562a-b. Isolated partial wing: TTNCM 39/2011/0399; TTNCM 39/2011/0431; TTNCM 39/2011/0541; TTNCM 39/2011/0542; NHMUK PI I.11488. Indeterminable: TTNCM 39/2011/0452; TTNCM 39/2011/0585; TTNCM 39/2011/0635; TTNCM 39/2011/1004.

Order Coleoptera Linnaeus 1758 [125]

Family Cupedidae Laporte 1836 [177]

gen. et sp. indet

***Assigned material.* –** TTNCM 39/2011/0624, isolated forewing (6.3 mm long, 1.7 mm wide); TTNCM 39/2011/0712, isolated partial forewing (11.5 mm long, 2.1 mm wide).

Family *Incertae sedis*

gen. et sp. indet

***Assigned material.* –** Almost complete individuals: TTNCM 39/2011/0365; TTNCM 39/2011/0377; TTNCM 39/2011/0640; TTNCM 39/2011/0643; TTNCM 39/2011/0644; TTNCM 39/2011/0645; TTNCM 39/2011/0666; TTNCM 39/2011/0667; TTNCM 39/2011/0697 & TTNCM 39/2011/0698 (part and counterpart); TTNCM 39/2011/0709; CAMSM TN 3199. Partial individuals: TTNCM 39/2011/0366; TTNCM 39/2011/0374; TTNCM 39/2011/0375; TTNCM 39/2011/0376; TTNCM 39/2011/0378; TTNCM 39/2011/0379; TTNCM 39/2011/0380; TTNCM 39/2011/0381; TTNCM 39/2011/0382; TTNCM 39/2011/0597; TTNCM 39/2011/0622; TTNCM 39/2011/0663 & TTNCM 39/2011/0664 (part and counterpart); TTNCM 39/2011/0669; TTNCM 39/2011/0670; TTNCM 39/2011/0671; TTNCM 39/2011/0672; TTNCM 39/2011/0673; TTNCM 39/2011/0708; TTNCM 39/2011/0710; TTNCM 39/2011/0711; NHMUK PI I.11486; NHMUK PI I.11487; NHMUK PI I.11497; NHMUK PI I.11500; CAMSM TN 3202. Isolated bodies: TTNCM 39/2011/0726; TTNCM 39/2011/0725. Isolated skeletal elements: TTNCM 39/2011/0441; TTNCM 39/2011/0443; TTNCM 39/2011/0471; TTNCM 39/2011/0496; TTNCM 39/2011/0662; TTNCM 39/2011/0695. Isolated forewings: TTNCM 39/2011/0363; TTNCM 39/2011/0367; TTNCM 39/2011/0386; TTNCM 39/2011/0387; TTNCM 39/2011/0388; TTNCM 39/2011/0406; TTNCM 39/2011/0471; TTNCM 39/2011/0596; TTNCM 39/2011/0598; TTNCM 39/2011/0599; TTNCM 39/2011/0600; TTNCM 39/2011/0601; TTNCM 39/2011/0602; TTNCM 39/2011/0603; TTNCM 39/2011/0604; TTNCM 39/2011/0605; TTNCM 39/2011/0606; TTNCM 39/2011/0607; TTNCM 39/2011/0608; TTNCM 39/2011/0609; TTNCM 39/2011/0610; TTNCM 39/2011/0613; TTNCM 39/2011/0614; TTNCM 39/2011/0615; TTNCM 39/2011/0616; TTNCM 39/2011/0617; TTNCM 39/2011/0620; TTNCM 39/2011/0621; TTNCM 39/2011/0627; TTNCM 39/2011/0630; TTNCM 39/2011/0631; TTNCM 39/2011/0668; TTNCM 39/2011/0713; TTNCM 39/2011/0714; TTNCM 39/2011/0715; TTNCM 39/2011/0717; TTNCM 39/2011/0718; TTNCM 39/2011/0719; TTNCM 39/2011/0720; TTNCM 39/2011/0721; TTNCM 39/2011/0722; TTNCM 39/2011/0723; TTNCM 39/2011/0724; TTNCM 39/2011/0727; TTNCM 39/2011/0728; TTNCM 39/2011/0760a-b; TTNCM 39/2011/0998; TTNCM 39/2011/1002; NHMUK PI I.11484;

NHMUK PI I.11491; NHMUK PI; I.11495; NHMUK PI I.11496 & NHMUK PI I.11494 (part and counterpart); NHMUK PI I.11498; NHMUK PI I.11502; CAMSM TN 3203; CAMSM 3204. Isolated partial forewings: TTNCM 39/2011/0373; TTNCM 39/2011/0407; TTNCM 39/2011/0493; TTNCM 39/2011/0498; TTNCM 39/2011/0611; TTNCM 39/2011/0612; TTNCM 39/2011/0618; TTNCM 39/2011/0619; TTNCM 39/2011/0623; TTNCM 39/2011/0625; TTNCM 39/2011/0628 & TTNCM 39/2011/0629 (part and counterpart); TTNCM 39/2011/0716; TTNCM 39/2011/0949. Indeterminable: TTNCM 39/2011/0447; TTNCM 39/2011/0448; TTNCM 39/2011/0449; TTNCM 39/2011/0450; TTNCM 39/2011/0451; TTNCM 39/2011/0452; TTNCM 39/2011/0453; TTNCM 39/2011/0636; TTNCM 39/2011/0641a-b; TTNCM 39/2011/0657; TTNCM 39/2011/0658; TTNCM 39/2011/0659; TTNCM 39/2011/0660; TTNCM 39/2011/0661; TTNCM 39/2011/0674; TTNCM 39/2011/0675; TTNCM 39/2011/0676; TTNCM 39/2011/0677; TTNCM 39/2011/0678; TTNCM 39/2011/0679; TTNCM 39/2011/0680 & TTNCM 39/2011/0681 (part and counterpart); TTNCM 39/2011/0682; TTNCM 39/2011/0683; TTNCM 39/2011/0684a-b; TTNCM 39/2011/0685; TTNCM 39/2011/0686; TTNCM 39/2011/0687; NHMUK PI I.11501.

***Remarks.* –** TTNCM 39/2011/0640 is figured in Williams *et al.* [43] (Fig 3B). At least two elytra morphotypes can be identified, including smooth (TTNCM 39/2011/0727) and striated elytra (TTNCM 39/2011/0614), indicating that the beetle fauna of Strawberry Bank was likely to consist of several species. Other beetle elements consist of well-preserved partial bodies (TTNCM 39/2011/0644).

Superorder Amphiesmenoptera Kiriakoff 1948 [178]

Order *Incertae sedis*

***Assigned material.* –** TTNCM 39/2011/0996a-b, isolated partial wing (9 mm long, 2.2 mm wide); TTNCM 39/2011/1009, isolated partial wing (6.5 mm long, 3 mm wide); TTNCM 39/2011/1010, isolated partial wing (5.2 mm long, 3 mm wide).

Order Necrotrichoptera Engel 2022 [156]

Family Necrotauliidae Handlirsch 1906 [121]

Genus *Necrotaulius* Handlirsch 1906 [121]

Necrotaulius parvulus Geinitz 1884 [67]

***Holotype.* –** FGWG 119/7, isolated wing. Grimmen Formation, 'Green Series'; Early Jurassic: Lower Toarcian of Dobbertin, Mecklenburg-Vorpommern, Germany.

***Assigned material.* –** TTNCM 39/2011/0733, isolated wing (7.3 mm long, 3 mm wide).

***Remarks.* –** See above for the remarks and discussion of *N. parvulus* for the Alderton Hill specimens.

***References.* –** [45, 67, 78].

Order Mecoptera Packard 1886 [160]

Family Orthophlebiidae Handlirsch 1906 [121]

Assigned material.* –** TTNCM 39/2011/0400,*** isolated wing (9.9 mm long, 2.2 mm wide).

Family *Incertae sedis*

gen. et sp. indet.

Assigned material.* –** TTNCM 39/2011/0546,*** isolated wing (6.1 mm long, 3.4 mm wide).

Order Diptera Linnaeus 1758 [125]

Family Limoniidae Speiser 1909 [173]

Genus *Architipula* Handlirsch 1906 [121]

Architipula sp.

***Assigned material.* –** TTNCM 39/2011/0402, isolated wing (7.1 mm long, 1.9 mm wide).

References. – [77].

Order Neuroptera Linnaeus 1758 [125]

Family *Incertae sedis*

gen. et sp. indet.

***Assigned material.* –** TTNCM 39/2011/0423; TTNCM 39/2011/0432; TTNCM 39/2011/0433; TTNCM 39/2011/0553; TTNCM 39/2011/1011; TTNCM 39/2011/1012.

Superorder Panorpida

Family *Incertae sedis*

gen. et sp. indet.

***Assigned material.* –** Isolated wing: TTNCM 39/2011/0539. Isolated partial wings: TTNCM 39/2011/0368; TTNCM 39/2011/0372; TTNCM 39/2011/0390; TTNCM 39/2011/0404; TTNCM 39/2011/0412; TTNCM 39/2011/0418; TTNCM 39/2011/0425; TTNCM 39/2011/0428; TTNCM 39/2011/0430; TTNCM 39/2011/0436; TTNCM 39/2011/0437; TTNCM 39/2011/0548.

***Remarks.* –** See supplementary material for individual measurements and descriptions.

Palaeoentomofaunal overview

Alderton Hill

From the 370 individual insects documented and examined from Alderton Hill, 29.2% (n = 108; Table 4) of the total assemblage was considered indeterminate due to poor preservation and

therefore could not be confidently assigned to any taxonomic group; these specimens were not included within further taxonomic analysis. The remaining 70.8% (n = 262) could be taxonomically identified to at least the level of order (Table 4). Of these individuals, 37.3% (n = 138) could be further assigned to family level, whilst 31.9% (n = 118) and 20.3% (n = 75) could be further assigned to genera and species respectively (Table 4). The 12 insect orders identified from the 262 identifiable individuals collected at Alderton Hill are Hemiptera (true bugs), Coleoptera (beetles), Orthoptera (grasshoppers), Neuroptera (lacewings), Odonata (dragonflies), Diptera (flies), Mecoptera (scorpionflies), Blattodea (cockroaches), Necrotrichoptera (caddisflies), Reculida ('winged insects'), ?Dermaptera (earwigs) and Ephemeroptera (mayflies); and their relative abundance is shown in Figure 6A.

Table 4. Number and percentage of Toarcian insect specimens attributed to each taxonomic rank at both localities at Alderton Hill and Strawberry Bank. Percentages are given as a proportion of the total number of individuals.

Assemblage breakdown	Alderton Hill insect assemblage No. (%)	Strawberry Bank insect assemblage No. (%)
**Number of specimens** (i.e. assigned an accession number)	363	386
**Number of individuals** (i.e. multiple individuals on one specimen)	370	799
**Number of indeterminate individuals**	108 (29.2)	240 (30)
Number of individuals assigned to **order**[a]	262 (70.8)	559 (70)
Number of insect **orders**[a] identified	12	11
Number of individuals assigned to **family**	138 (37.3)	38 (4.8)
Number of insect **families** identified	20	12

Number of individuals assigned to **genera**	118 (31.9)	12 (1.5)
Number of insect **genera** identified	24	7
Number of individuals assigned to **species**	75 (20.3)	4 (0.5)
Number of insect **species** identified	21	3

[a]The total number of insect orders for Strawberry Bank also includes the superorders Amphiesmenoptera and Panorpida.

Figure 6. Pie-charts showing the number and percentages of taxonomically identifiable insect specimens within each order, grouped by abundance from **(A)** Alderton Hill, Gloucestershire and **(B)** Strawberry Bank, Somerset: Note that the 'winged insects' Amphiesmenoptera and Panorpida are superorders, but are still included within this comparison.

The Hemiptera of Alderton Hill are the most diverse and abundant order within the fossil insect assemblage, comprising 22.1% (n = 58; Fig 6A) of all the identifiable insect specimens collected from this locality. This order is represented by four families (Table 5). The majority of the hemipteran material, in particular those attributed to Fulgoridiidae, are comprised of isolated wings. The genus *Margaroptilon* is the most common taxa within this order, and is represented by well-preserved forewings with spotted pigmentation (i.e. NHMUK PI I.11266; Fig 4C). Other hemipteran elements also include partial bodies (Figs 4G – 3I), which c[illegible]ot be identified further. Coleoptera is the second most common insect order of the Alderton Hill fossil assemblage (20.6%; n = 54). This order is mostly represented by isolated elytra, but also paired elytra and partial individuals, and the poor preservation prevents further identification (Table 5). However, at least three elytra morphotypes can be identified (Figs 4K – Fig 4M), indicating that the beetle fauna is likely to consist of several genera or species. Orthoptera (13.4%, n = 35; Fig 6A) are attributed to three families (Table 5) and are primarily represented by fragmented, yet well-preserved tegmina. Some tegmina possess the original striped and spotted pigmentation (Figs 3I – 3L), which aids their taxonomic assignment. Isolated skeletal elements including saltatorial hind legs are also present (Fig 3M). Whilst

Neuroptera are relatively abundant from Alderton Hill (12.2%, n = 32; Fig 6A), they are mostly represented by poorly preserved, yet identifiable isolated wings. However, several well-preserved examples of Neuroptera are present (Figs 5A – 5F), representing three families (Table 5).

Table 5. Number and percentage of specimens attributed to each family, genera and species within each order at Alderton Hill. Taxa are listed in the order they appear within the systematic taxonomy. Percentages are given as a proportion of the total number of individuals attributed to each order.

**Order**	**Family**	**Genera and species**	**No. of assigned specimens**	**Percentage of taxa within order (%)**
**Ephemeroptera**	-	-	1	100
**Odonata**	Heterophlebiidae	*Heterophlebia buckmani*	7	23.3
	Liassogomphidae	*Heterothemis brodiei*	6	20
		Heterothemis sp.	4	13.3
	Protomyrmeleontidae	*Protomyrmeleon* sp.	1	3.3
		gen. et sp. indet.	1	3.3
	Incertae sedis	gen. et sp. indet.	11	36.8
**?Dermaptera**	-	-	1	100

**Reculida**	Geinitziidae	*Geinitzia carpentieri*	2	100
**Blattodea**	?Raphidiomimidae	*Liadoblattina blakei*	4	66.6
	Caloblattinidae	gen. et sp. nov.	1	16.7
	Incertae sedis	gen. et sp. indet	1	16.7
**Orthoptera**	Locustopsidae	*Locustopsis* sp.	7	20
		gen. et sp. indet	1	2.9
	Elcanidae	*"Elcana" cf. geinitzi*	4	11.4
		"Elcana" sp.	2	5.7
	Protogryllidae	*Protogryllus cf. dobbertinensis*	1	2.9
		Protogryllus magnus	1	2.9
		Protogryllus sp.	2	5.7
	Incertae sedis	gen. et sp. indet	17	48.5
**Hemiptera**	Fulgorididae	*Fulgoridium* sp.	9	15.5
		Margaroptilon brodiei	18	31
		gen. et sp. indet.	2	3.4
	Archijassidae	*Archijassus heeri*	1	1.7
	Progonocimicidae	-	2	3.4

	Gerromorpha	*Engynabis tenuis*	1	1.7
	Incertae sedis	gen. et sp. indet.	25	43.3
**Coleoptera**	*Incertae sedis*	gen. et sp. indet.	54	100
**Necrotrichoptera**	Necrotauliidae	*Necrotaulius parvulus*	4	80
		?*Necrotaulius* sp.	1	20
**Mecoptera**	Bittacidae	*Protobittacus handlirschi*	1	7.7
		gen. et sp. indet.	1	7.7
	Orthophlebiidae	*Orthophlebia brodiei*	1	7.7
		Orthophlebia sp.	9	69.2
	Pseudopolycentropodidae	*Pseudopolycentropus* sp.	1	7.7
**Neuroptera**	Osmylopsychopidae	*Actinophlebia intermixta*	3	9.4
		Actinophlebia sp.	1	3.1
	"Familia nova A"	*Paractinophlebia curtisii*	2	6.3
	"Prohemerobiidae"	*"Prohemerobius" aldertonensis*	3	9.4

		"Prohemerobius" sp.	3	9.4
		"Archeosmylus" alysius	6	18.8
		"Archeosmylus" complexus	2	6.3
		gen. et sp. indet.	1	3.1
	Incertae sedis	gen. et sp. indet.	11	34.2
**Diptera**	Limoniidae	*Architipula anglicana*	5	20
		Architipula sp.	1	4
		Grimmenia tillyardi	1	4
		Mesotipula slatteri	2	8
		gen. et sp. indet.	16	64

Odonata is represented by three families (Table 5) and accounts for 11.5% (n = 30) of the total Alderton Hill palaeoentomofauna (Fig 6A). The order is mainly represented by partial wing fragments which possess clear venation (Fig 3C and 3D) aiding identification, however complete, well-preserved forewings (Fig 3A) and hindwings (Fig 3B) are also present. Diptera comprises 9.5% (n = 25) of the fossil assemblage (Fig 6A), in which all specimens identified to family level are exclusively assigned to the family Limoniidae (craneflies) and primarily composed of isolated bodies and wings. Well-preserved isolated wings belonging to the order Mecoptera comprises 5% (n = 13) of the total insect assemblage (Fig 4Q and 3R; Fig 6A), and there are three families present. Blattodea comprise 2.3% (n = 6) (Fig 6A) and are represented

by rather well-preserved, isolated tegmina with the clavus attached (Figs 3G - 3H). One specimen, BRSMG Cg2374 & BRSMG Cg2375, is noticeably dissimilar to all other Toarcian cockroaches and therefore represents a new genus and species. Necrotrichoptera accounts for only 1.9% (n = 5) of the Alderton Hill palaeoentomofauna (Fig 6A), in which all specimens belong to the family Necrotauliidae (Fig 4N and 4O). Similarly, Reculida is also uncommon (0.8%; n = 2) (Fig 6A) and is only represented by partial, yet well preserved forewing fragments of *Geinitzia carpentieri* (Fig 3F). ?Dermaptera (NHMUK PI I.11403; Fig 3E) and Ephemeroptera (WARMS G 8084) are rare orders within the deposit, each representing 0.4% (n = 1) of the total insect assemblage (Fig 6A); these orders are all represented by poorly preserved fragmentary remains.

Strawberry Bank

Of the 799 individual insects from Strawberry Bank, 30% (n = 240; Table 4) of the insect assemblage are considered indeterminate due to poor preservation and therefore cannot be confidently assigned to any taxonomic group. Of the total insect assemblage, 70% (n = 559) can be taxonomically identified to at least order level (Table 4). Of these individuals, only 4.8% (n = 38) of the Strawberry Bank insect assemblage can be assigned to family level, whilst 1.5% (n = 12) and 0.5% (n = 4) can be assigned to genera and species (Table 4). Nine insect orders and two superorders are identified among 559 identifiable individuals from Strawberry Bank: Hemiptera, Coleoptera, Orthoptera, Neuroptera, Odonata, Diptera, Mecoptera, Blattodea, Dermaptera, Amphiesmenoptera and Panorpida; and their relative abundance is shown in Figure 6B.

Within the Strawberry Bank fossil insect assemblage, Coleoptera is the most abundant order comprising 74.2% (n = 415; Fig 6B) of all the identifiable insect specimens collected from this locality. The majority of the coleopteran material is represented by disarticulated and fragmented elytra which are compressed and rather badly preserved, and not possible to

identify beyond order level (Table 6). Similar to Alderton Hill, at least two elytra morphotypes can be identified, indicating different species. Hemiptera is the second most common insect order of the Strawberry Bank palaeoentomofauna (8.9%, n = 50) (Fig 6B), and can be assigned to two families (Table 6). This order is primarily represented by partial individuals (NHMUK PI I.11493) and isolated wings; wings attributed to *Margaroptilon* (Table 6) in particular possess spotted pigmentation (TTNCM 39/2011/0594). Orthoptera, the third most abundant insect order (6.6%, n = 37) (Fig 6B), is represented by three families (Table 6) and comprised of isolated, fragmented tegmina, some of which show original striped and spotted pigmentation (TTNCM 39/2011/0523). Odonata accounts for 3.6% (n = 20) of the Strawberry Bank palaeoentomofauna (Fig 6B) and can be attributed to only one family (Table 6). This order is mainly represented by partial wing fragments which possess some venation (e.g. TTNCM 39/2011/0516). Although disarticulated, segmented abdominal elements (TTNCM 39/2011/0362a-b) are also documented. Panorpida (2.3%, n = 13; Fig 6B) is represented exclusively by isolated and often poorly preserved wing fragments (e.g. TTNCM 39/2011/0436), preventing taxonomic refinement below superorder level (Table 6).

Table 6. Number and percentage of specimens attributed to each family, genera and species within each order at Strawberry Bank. Taxa are listed in the order they appear within the systematic taxonomy. Percentages are given as a proportion of the total number of individuals attributed to each order.

**Order**	**Family**	**Genera and species**	**No. of assigned specimens**	**Percentage of taxa within order (%)**
**Odonata**	Liassogomphidae	-	2	10
	Incertae sedis	gen. et sp. indet.	18	90

**Blattodea**	Caloblattinidae	*Rhipidoblattina* sp.	1	10
	Incertae sedis	gen. et sp. indet.	9	90
**Dermaptera**	Dermapteridae	*Trivenapteron moorei*	1	100
**Orthoptera**	Locustopsidae	*Locustopsis* sp.	1	2.7
	Elcanidae	-	13	35.1
	Protogryllidae	-	1	2.7
	Incertae sedis	gen. et sp. indet.	22	59.5
**Hemiptera**	Fulgoridiidae	*Margaroptilon brodiei*	2	4
		Margaroptilon sp.	5	10
	Archegocimicidae	-	3	6
	Incertae sedis	gen. et sp. indet.	40	80
**Coleoptera**	Cupedidae	-	2	0.5
	Incertae sedis	gen. et sp. indet.	413	99.5
**Amphiesmenoptera**	Necrotauliidae	*Necrotaulius parvulus*	1	25
	Incertae sedis	-	3	75
**Mecoptera**	Orthophlebiidae	-	1	50

	Incertae sedis	gen. et sp. indet.	1	50
**Diptera**	Limoniidae	*Architipula* sp.	1	100
**Neuroptera**	*Incertae sedis*	gen. et sp. indet.	6	100
**Panorpida**	-	-	13	100

Blattodea accounts for 1.8% (n = 10) of the palaeoentomofauna (Fig 6B), and is represented by only one family (Table 6). Similar to the rest of the Strawberry Bank insect assemblage, the material is preserved as disarticulated tegmina, with no hindwings present. Neuroptera comprises 1.1% (n = 6) of the Strawberry Bank fossil insect assemblage (Fig 6B) and is mostly represented by poorly preserved, isolated wings that are difficult to identify beyond order level (Table 6); however, some better preserved examples are present (TTNCM 39/2011/0423). Amphiesmenoptera comprises 0.7% (n = 4) of the palaeoentomofauna (Fig 6B), with one family identified (Table 6). Whilst the rest of these individuals cannot be taxonomically refined any further than superorder level, they most likely belong to either Lepidoptera or Necrotrichoptera. This material is represented exclusively by isolated, preserved wing and wing fragments (TTNCM 39/2011/1009). Whilst Dermaptera is uncommon within the Strawberry Bank palaeoentomofauna (0.2%, n = 1) (Fig 6B), a single tegmen was described by Kelly *et al.* [44] as a new species - *Trivenapteron moorei* (TTNCM 39/2011/0489), which was the first record of an earwig from the Pliensbachian-Toarcian of the UK [43]. Finally, Mecoptera and Diptera are rare within the Strawberry Bank deposit, and represent 0.4% (n = 2) and 0.2% (n = 1) of the total insect assemblage respectively (Fig 6B).

In summary, approximately 70% of the insect assemblages at both Alderton Hill and Strawberry Bank can be identified to order level, and whilst the Coleoptera, Hemiptera and Orthoptera are the three most common orders at both localities, their relative abundances differ (Fig 6). Alderton Hill is significantly more diverse at all taxonomic ranks recognised in the fossil assemblage (Table 3), as more specimens can be assigned to family (AH = 37.3%

cf. SB = 4.8%), genera (AH = 31. 9% cf. SB = 1.5%) and species (AH = 20.3% cf. SB = 0.5% (Table 3).

Feeding traits

The diet of 74.3% (n = 278) of the Alderton Hill fossil insect assemblage is classified as indeterminable. Specific diets for the majority of taxa cannot be inferred because of the low taxonomic resolution (e.g. Coleoptera). At present-day, carnivorous, herbivorous and parasitic examples are present in almost all insect orders; demonstrating that generalisations cannot be made. For taxa where their specific diets can be determined (n = 92), 58.7% (n = 54) are herbivorous (e.g. Hemiptera: Auchenorrhyncha and Coleorrhyncha; Orthoptera: Elcanidae, Locustopsidae and Protogryllidae) and 41.3% (n = 38) are carnivorous (e.g. Blattodea: Raphidiomimidae; Mecoptera: Bittacidae; and Odonata). However, the low numbers in each feeding trait mean that analysis is not very representative. As the majority of the Strawberry Bank palaeoentomofauna is comprised of Coleoptera, which are largely unidentifiable beyond order level, further insights in the trophic feeding of this insect community and specific diets cannot be inferred.

Taphonomic analysis

Alderton Hill

Based on the matrix of the insect specimens examined, all of the insect material collected from Alderton Hill to date appears to have come from the limestone nodules within the 'Fish Bed' (Bed 10 of Richardson [80]). The insect material of Alderton Hill is mostly preserved as highly disarticulated, fragmented compression fossils. Some Coleoptera are preserved in three-dimensions, but show minor compression (NHMUK PI I.11318; NHMUK PI I.11369). As the insects are preserved in early diagenetic nodules formed within fine-grained sedimentary

deposits, the intricate detail of the insects is well preserved, including wing venation and evidence of colour pigmentation and patterns.

From the 370 individual insects documented and examined, 20.5% (n = 76) could not be taphonomically assigned because of poor preservation or, in a few cases, they were not available for examination. These specimens were assigned the taphonomic grade '??'. Of the taxonomically identifiable specimens, 56.1% (n = 147) of the total insect assemblage were comprised of heavily sclerotised taxa i.e. Hemiptera, Coleoptera and Orthoptera. No aquatic larval stages were observed within the collections, and thus all of the specimens examined were represented by terrestrial adult life stages, including flying terrestrial and semiaquatic taxa.

The taphonomic grades from 1 – 2d (Table 2) were assigned to 294 individuals (Fig 7A). Whilst a wide range of preservation was observed among the Alderton Hill insects (Table 3), the material was mostly represented by isolated wings (n = 198, 67.3%; Fig 7A). The most common primary taphonomic grade within the deposit was isolated forewing/s (grade 2b) with 101 (34.4%) individuals; 50 (17%) and 51 (17.3%) of these forewings could be assigned to the secondary taphonomic grades 2b.1 and 2b.2 respectively (Fig 7A). Eighty-six individuals (29.3%) were isolated unassigned wing/s (grade 2d), with 12 (4.1%) being almost complete (grade 2d.1) and 74 (25.2%) wing fragments (grade 2d.2) (Fig 7A); the latter was the most common secondary taphonomic grade recorded from the insect assemblage. Seventy-one individuals (24.1%) were isolated bodies (grade 2a); 12 (4.1%) and 59 (20.1%) were almost complete (grade 2a.1) and skeletal body elements (grade 2a.2) (Fig 7A). Twenty-five individuals (8.5%) were bodies with forewings and/or hindwings (grade 1); 7 (2.4%) insects were assigned to the secondary taphonomic grade 1.2, and 18 (6.1%) individuals to 1.3 (Fig 7A). No complete, fully articulated individuals (grade 1.1) have been collected from this locality to date (Fig 7A). The most complete insect collected from Alderton Hill was the paratype of *Heterophlebia buckmani* (NHMUK PI I.3988), comprised of a head, thorax and fore- and hindwings, with only the distal half of abdomen absent [64]; however, this type specimen was

not located within the NHMUK collection during this study and is presumed lost. Whilst this specimen could not be included within this research as it could not be re-examined, it demonstrates that more complete individuals were preserved at Alderton Hill.

The rarest primary taphonomic grade within the deposit was isolated hindwing/s (grade 2c) with 11 individuals (3.7%); 4 (1.4%) of these hindwings were almost complete (grade 2c.1) and 7 (2.4%) were fragments (grade 2c.2) (Fig 7A). The level of disarticulation is high in all insect orders; with only 8.5% (n = 25) of the total assemblage being considered either partially articulated or fully articulated (e.g. abdomen associated with elytra and thorax); therefore, much of the Alderton Hill collection consisted of fully disarticulated insect elements (n = 269).

Figure 7. The frequency of individual insect specimens in each taphonomic grade for: **(A)** Alderton Hill, Gloucestershire (n = 291); **(B)** Strawberry Bank, Somerset (n = 518). The number and percentage in each primary taphonomic grades are given in red boxes, and the secondary taphonomic grade counts and percentages are stated above each individual bar. The solid green and orange bars denote complete specimens or element, and the paler green and orange bars denote partial specimens or elements.

In total, 230 (78.2%) of the individuals assigned a taphonomic grade could be taxonomically assigned to at least order level (Table 3). A Chi-squared test indicated that there was a statistically significant association between the taxonomic rank and the taphonomic grade at order level ($x^2 = 234.58$, df = 99, p = < 0.001). The excellent preservation of bodies with wings (grade 1), isolated complete bodies (grade 2a.1) and wings >75% complete (i.e. grades 2b.1 and 2c.1) in the majority of the specimens allowed for a high level of taxonomic identification. Where the wings were less than 75% complete (i.e. grades 2b.2, 2c.2 and 2d.2) or there were only isolated skeletal elements (grade 2a.2), the taxonomic identification was poorer. In order to determine the degree to which each primary taphonomic grade contributed to the total Chi-squared score at order level, Pearson residuals were calculated for each entry in the Chi-squared contingency table and displayed graphically (Fig 8). Within the individual orders, the largest range of taphonomic grades were observed within the Hemiptera, with 30% (n = 15)

of this material being assigned to 2b.2. Coleoptera were primarily represented by isolated or paired elytra, as 45.5% (n = 20) of the coleopteran material was assigned to 2b.1 (Fig 8).

Figure 8. Calculated Pearson residuals for each entry in the Chi-squared contingency table at order level, used to determine the degree to which each primary taphonomic grade contributed to the total Chi-squared score for the Alderton Hill palaeoentomofauna. The colour of each circle represents its residual value: positive residuals are blue and negative residuals are red, indicating a positive and negative association respectively between taxonomy and taphonomy. The size of the circle is proportional to the degree of cell contribution to the relationship.

The Alderton Hill insect fossils had a minimum preserved length of 0.3 mm and a maximum of 45 mm, with a median preserved length of 6.4 mm and a mean preserved length of 7.98 mm (SD = 6.10 mm; n = 318) (Fig 9A). Preserved specimen widths ranged from 0.1 mm to 13.9 mm, with a median preserved width of 2.3 mm and a mean preserved width of 2.84 mm (SD = 2.12 mm; n = 318) (Fig 9C); only 5% of the insect material was greater than 20 mm long and 11.9% was greater than 5 mm wide.

Figure 9. Preserved length and width of all insect elements recorded from: **(A, C)** Alderton Hill, Gloucestershire; **(B, D)** Strawberry Bank, Somerset.

Strawberry Bank

Similar to Alderton Hill, the levels of disarticulation were high in all insect orders at Strawberry Bank; only 9.3% (n = 49) of the total insect assemblage was considered either partially articulated or fully articulated, therefore much of the deposit consisted of fully disarticulated insect elements (n = 477) (Fig 7B).

As a high proportion (74.2%) of the specimens from Strawberry Bank are taphonomically robust Coleoptera, most of the material is disarticulated but preserved in three-dimensions, with minor amounts of compression. The remainder of the material is preserved as highly

disarticulated, and fragmented compression fossils, similar to Alderton Hill. From the 799 individual insects documented and examined from the Strawberry Bank insect assemblage, 34.2% (n = 273) could not be taphonomically assigned and were given the taphonomic grade '??'. Of the taxonomically identifiable specimens, 89.8% (n = 502) of the total insect assemblage of Strawberry Bank was comprised of heavily sclerotised taxa i.e. Coleoptera, Hemiptera and Orthoptera. The taphonomic grades from 1–2d (Table 2) were assigned to 526 individuals (Fig 7B).

Similar to Alderton Hill, the insect material of Strawberry Bank was mostly represented by isolated wings (n = 436, 82.9%; Fig 7B). The commonest primary taphonomic grade within the deposit was isolated forewing/s (grade 2b) with 337 (64.1%) individuals. From this, 308 (58.6%) of these forewings were almost complete (grade 2b.1) and 29 (5.5%) were fragments (grade 2b.2) (Fig 7B). The former was the most common secondary taphonomic grade recorded from the Strawberry Bank insect assemblage.

Ninety-seven individuals (18.4%) were isolated unassigned wing/s (grade 2d), with 21 (4%) and 76 (14.4%) of these wings belonging to the secondary taphonomic grades 2d.1 and 2d.2 respectively (Fig 7B). Forty-nine individuals (9.3%) were bodies with forewings and/or hindwings (grade 1); 15 (2.9%) insects were assigned to the secondary taphonomic grade 1.2, and 34 (6.5%) individuals to 1.3 (Fig 7B). Forty-one individuals (7.8%) were isolated bodies (grade 2a); 5 (1%) and 36 (6.8%) were given the secondary taphonomic grades 2a.1 and 2a.2 respectively (Fig 7B). No insects could be assigned to taphonomic grade 1.1 (Fig 7B), meaning no complete, fully articulated individuals have been recovered from Strawberry Bank to date. The rarest primary taphonomic grade within this deposit was isolated hindwing/s (grade 2c) with only 2 individuals (0.4%) assigned to taphonomic grade 2c.1 (Fig 7B).

The Strawberry Bank insects had a minimum and maximum preserved length of 0.8 mm and 51.2 mm respectively, with a median preserved length of 5 mm and a mean preserved length of 6.46 mm (SD = 5.44 mm; n = 275; Fig 9B). Preserved width ranged from 0.3 mm to 18.4 mm, with a median preserved width of 2.2 mm and a mean preserved width of 2.54 mm (SD

= 1.82 mm; n = 275; Fig 9D). The distribution of the length and width shows a greater frequency of small sized elements at Strawberry Bank. Further statistical comparison of the size data from Strawberry Bank and Alderton Hill was not undertaken, as the taphonomic and taxonomic bias at Strawberry Bank render any results from this location meaningless.

Discussion

The diversity of the Alderton Hill palaeoentomofauna, comprising 12 insect orders (Fig 6A), is comparable to the number of extant insect orders in present-day assemblages from analogous latitudes (30°– 40°N). For example, in the wetlands of the Zaranik Protectorate in North Sinai, Egypt (30.2° N), 11 insect orders occur [179]. Perhaps the most analogous present-day environment to Alderton Hill during the Toarcian is the Azores archipelago in the Atlantic (37.7° N). In both cases, the environment is a collection of small islands with high rainfall, that are over 1000 km from large landmasses (the Azores are ~1400 km from mainland Europe, compared to Alderton Hill being >1000 km from Laurentia and Eurasia: Fig 1). On the Azores, 19 insect orders occur (n= 5979) [180] of which 15 had evolved before the Toarcian and 10 of these are recorded within the Alderton Hill palaeoentomofauna. One order present at Alderton Hill, Mecoptera, is not documented from the Azores, and Reculida (also present at Alderton Hill) went extinct in the Jurassic. The similarity in order diversity at Alderton Hill, compared to present-day assemblages from analogous latitudes, suggests that the palaeoentomofauna is broadly representative of the number of orders you would expect to see in the Toarcian at this palaeolatitude.

Fossil insect collections can be biased towards better-preserved material with more diagnostic characters that allow the specimen to be readily identified e.g. isolated wings [181]. Based on our observations of the Alderton Hill assemblage, we consider that it is unlikely that there is a large collection bias for the following reasons: (1) the order diversity is similar to insect assemblages at similar latitudes today; (2) the encompassing nodules are fine-grained and

pale in colour, making it easy to visually distinguish the generally darker well-preserved fossils; (3) more delicate insects orders including Neuroptera and Diptera are well represented; (4) insect specimens in general are small and require further examination, meaning it is likely that all potential insect material was collected in the field; and (5) much of the material was not identified at the time of collection, again suggesting that all potential insect material that was found was collected.

High abundance and diversity of Odonata in present-day insect communities is regarded as an indication of good ecological health [182]. We therefore interpret the abundance (11.5%; n = 30) and diversity (represented by 3 families) of Odonata observed at Alderton Hill as indicative of a well-established and healthy insect ecosystem during the Toarcian. Hemiptera are not only the most diverse (4 families and 4 genera) order at Alderton Hill but are also the most abundant (22.1%; n = 58). Their abundance exceeds that in the present-day Azores, where they comprise ~17% of the total insect assemblage [180]. We interpret the higher abundance and diversity of these herbivorous insects at Alderton Hill to be directly linked to the ready abundance of vegetation during the warm and mostly humid greenhouse climate of the T-OAE. In particular, the hemipteran family Progonocimicidae (Coleorrhyncha) identified within the Alderton Hill insect assemblage (NHMUK PI I.11465; Fig 3E) is characteristic of much warmer and humid conditions and would, most likely, have inhabited flora such as cheirolepid conifers [183], which were dominant during the T-OAE [39, 184]. Our data also supports the interpretation of Szwedo [41] who proposed that the Hemiptera, in particular the suborder Coleorrhyncha, diversified during the Jurassic in response to the changes in vegetation during the T-OAE. Similar to Hemiptera, the relatively high abundance of Orthoptera (13.4%; n = 35) within the Alderton Hill palaeoentomofauna, compared to 1% in the Azores today [180], is here attributed to their mostly herbivorous diet being well suited to the abundance of vegetation during the T-OAE. Coleoptera, whilst being the second most abundant order at Alderton Hill (20.6%; n = 54), are more common in the Azores today (32%) [180]. However, based on our current knowledge of this order, the high diversity within

Coleoptera does not appear to have been achieved until the Late Jurassic (155-160 Ma) [117]; likely explaining the differences in dominance between the Toarcian and present-day.

The three most abundant orders at Alderton Hill (Hemiptera, Coleoptera and Orthoptera) are highly sclerotised taxa and together they comprise 56.1% (n = 147) of the total insect assemblage. This proportion is slightly elevated in comparison to the proportion of highly sclerotised taxa at present-day on the Azores archipelago, where Hemiptera, Coleoptera and Orthoptera comprise 50.4% (n = 3011) [180] of the total insect community across all of the islands. This could reflect one or more of the following for the Alderton Hill entomofauna:

(1) Higher abundance in the life assemblage because highly sclerotised taxa were better adapted to fluctuations in water, temperature and gases during the climatic extremes of the T-OAE. For instance, in Coleoptera, the elytra provide a mechanical barrier against direct water loss from the body surface, thereby increasing the organism's tolerance to desiccation [185] and enabling Coleoptera to withstand rapid temperature changes [186].

(2) Preferential preservation of highly sclerotised taxa and elements including elytra, hemelytra and tegmina. Decay studies of present-day insects [187, 188] have shown that highly sclerotised forewings are the strongest and most flexible elements, and therefore are more resistant to transportation and decay compared to those with less sclerotisation. In contrast, less robust orders (e.g. soft-bodied Diptera) could have been preferentially removed during specimen decay and decomposition.

(3) Alternatively, the concentration of highly sclerotised elements within the deposit could be due to predators, such as fish, marine reptiles or other insectivorous reptiles e.g. pterosaurs [75] favouring consumption of the insect bodies over their wings; therefore leading to a concentration of highly sclerotised isolated wings [189, 190].

In contrast to Alderton Hill, sclerotised taxa, specifically Coleoptera dominate the insect assemblage from Strawberry Bank (74.2%, n = 415). As this proportion is well in excess of

present-day communities from similar palaeolatitudes, we interpret the Strawberry Bank insect assemblage to be a direct reflection of preferential preservation of sclerotised taxa. A preservational bias towards highly sclerotised orders at Strawberry Bank is further supported by: the greater frequency of small sized fragmentary elements (Fig 9); the poorer level of overall preservation, which for most of the specimens, prevents identification below order and family level; the greater proportions of isolated and poorly preserved wings (Fig 7B); and the fact that several less robust orders including Neuroptera and Diptera are well represented at Alderton Hill but are an order of magnitude lower in abundance at Strawberry Bank (Neuroptera: 1.1%, n = 6 at SB, compared to 12.2%, n = 32 at AH; Diptera: 0.2%, n = 1 at SB, compared to 9.5%, n = 25 at AH) (Fig 6). Williams *et al*. [43] suggested that the abundance of Coleoptera at Strawberry Bank reflected either genuinely higher abundance, collector bias or differential preservation. However, they went on to note that collection bias was unlikely as many of the insect specimens within the Moore collection were difficult to distinguish visually from the matrix, poorly preserved, and fragmentary, suggesting that Moore is more likely to have collected most of the insect material found in the field.

The poor preservation of the Strawberry Bank insects can be attributed to both the coarse-grained nature of the deposit [43] and their deposition nearshore where waves and current action is more prevalent. We note however, that the fossil insect preservation is in contrast to other exceptionally well-preserved fauna collected from Strawberry Bank, including phosphatized soft tissue and three-dimensional preservation of marine nekton [90]. Although all of the fauna is reportedly from a single layer of nodules [43, 90], the difference in preservation may be because the insects are from a slightly different stratigraphic level within the nodular horizon. In contrast to Strawberry Bank, the majority of insects found within the Alderton Hill insect assemblage are well-preserved, including wings showing intricate veining and evidence of colour pigmentation and patterns (Figs 2 - 5). These well-preserved wings allow for a high level of taxonomic identification, as demonstrated by the statistically significant relationship between taphonomic grade and taxonomic rank (Fig 8). Both lower energy

conditions during deposition (as indicated by the sedimentology), and the finer grain size (which allows for more delicate and detailed fossils to be preserved) are likely to be responsible for the better preservation of insects at Alderton Hill.

The level of disarticulation of the insect material from Alderton Hill (91.5%; Fig 7A) and Strawberry Bank (90.7%; Fig 7B) is similar and commensurate with actualistic decay studies of present-day insects. For example, Duncan *et al.* [189], using cockroaches, determined that the average time required for complete mechanical disarticulation of the individual was 18 to 70 hours. The tegmen are the last elements of a cockroach to break down, and this occurs through the loss of the clavus in the anal region by separation along the wing venation [189]. Of the six cockroaches present within the assemblage, all but one of the tegmina from Alderton Hill are complete with the clavus (Fig 2G and Fig 2H), strongly suggesting that burial was likely to be rapid (< 70 hours). This preservation provides further evidence that the fossil insects of Alderton Hill are representative of the life assemblage.

The sedimentology and the associated invertebrate fauna of the insect-bearing horizon of Alderton Hill and Strawberry Bank indicate that they were deposited within shallow-marine waters, with Strawberry Bank probably being closer to land. Whilst some insects may have been actively flying, or drifting on wood and plant matter over these shallow-marine areas, most of the insects from these localities must be allochthonous.

The allochthonous nature of the fossil assemblages may explain why no immature insect fossils have been found in the collections from Alderton Hill or Strawberry Bank, but we also note that fossils representing immature insects, even at well preserved sites, are rare [191]. For many holometabolous insects (those undergoing complete metamorphosis), larval stages often possess few features that are robust enough to be preserved, although fossilised mouthparts have provided intriguing insights into the ecology of some species [192]. For some hemimetabolous insects (with incomplete metamorphosis) e.g. Hemiptera, juveniles can superficially look almost identical to adults, just smaller and without wings meaning that they might easily be misidentified as adults and therefore immature forms may be unrepresented

within the current collections. Many hemimetabolous orders, however, do have very distinct larval stages, e.g. Odonata, and for this group the larval stages are not only robust enough to be preserved, but should also greatly outnumber adults for large parts of a given year (as they dominate the life cycle, e.g. some species have as many as 15 moults). Thus, if a fossil site were truly representative of an aquatic habitat, then one might expect to encounter at least some immature forms.

Whilst the exact reason(s) why only one stratigraphic level within this event contains an abundant and diverse assemblage awaits further study, we suggest a possible explanation for the high overall abundance of insects at Alderton Hill and Strawberry Bank, is the highly unusual environmental conditions of the T-OAE. Periodic low oxygen conditions at Alderton Hill and most other epicontinental locations during the T-OAE are well established through a wide range of sedimentary, geochemical and biological proxies (see introduction). Noting the high abundance of the small early teleost fish *Leptolepis* and the larger Pacyco[illegible]ds co-occurring with the insects at both localities [43, 65; new data set from this study, see Fig 2D], we postulate that this could be the direct result of shoals of fish being forced into shallower nearshore waters during de-oxygenation, and dying in large numbers because of the lack of resources. The decaying floating fish would then have become an attractive food source for the higher trophic and predatory insects, including Odonata (Heterophlebiidae and Liassogomphidae), and Mecoptera (Bittacidae)). Similar environmental events, often known as jubilees, are observed at present-day; in these cases, periods of intense de-oxygenation drive fish into surface-waters and shallow-water areas as they seek oxygenated water [193, 194, 195, 196]. The lack of immature insects at Alderton Hill, despite the excellent preservation of several ‘delicate’ insect orders, perhaps provides further support for a jubilee event composed of an assemblage of predominantly feeding adults in a coastal habitat.

Conclusions

Through the re-examination of all fossil insect collections from Alderton Hill, Gloucestershire, UK, we describe a rich and diverse palaeoentomofauna represented by 12 insect orders, 20 families and 24 genera and 21 species. The quality of preservation allows for a high level of taxonomic identification, reflected by the locality having previously yielded 30 type specimens. The diversity of the Alderton Hill palaeoentomofauna at order level is comparable to present-day insect assemblages from equivalent latitudes (30°– 40°N), including the Azores archipelago, that is geographically and climatically interpreted to be most similar to the conditions in the UK during the Toarcian. This similarity in order diversity, in addition to the preservation of delicate taxa and the lack of a strong taxonomic bias lead us to interpret that the Alderton Hill palaeoentomofauna is a good overall reflection of the insect community during the T-OAE at this palaeolatitude.

The particularly high abundance of the Hemiptera and Orthoptera at Alderton Hill is likely to have been driven by dense vegetation on the neighbouring islands and landmasses, that would have thrived in the warm humid conditions of the T-OAE. The high diversity of Hemiptera present at Alderton Hill supports the interpretation that changes in the vegetation in response to climate fluctuations may have driven the evolution of this order. A slightly higher proportion of sclerotised taxa in the Alderton Hill assemblage compared to present-day could reflect that their less permeable exoskeletons provided greater tolerance to fluctuations in fluids and temperature at this time. Alternatively, there could be a minor taphonomic bias towards more robust taxa and elements, or preferential predation of insect bodies.

In comparison to Alderton Hill, the coeval UK insect assemblage of Strawberry Bank, Somerset is less diverse with 2 superorders, 9 insect orders, 12 families, 7 genera and 3 species, and is heavily dominated by sclerotised Coleoptera elytra. This taxonomic and taphonomic bias is despite the fact that more insect specimens have been collected from Strawberry Bank (n = 799) than at Alderton Hill (n = 370).

The dominance of isolated wings and wing fragments, in addition to the high levels of disarticulation amongst the insect material from both Alderton Hill and Strawberry Bank is consistent with actualistic decay studies of present-day insects. The greater proportion of smaller insect elements at Strawberry Bank compared to Alderton Hill reflects greater fragmentation and supports the interpretation of taphonomic bias within the Strawberry Bank material. In contrast to the poorly preserved insects of Strawberry Bank, the majority of the wings within the Alderton Hill insect assemblage are well-preserved, with intricate veining and colour pigmentation patterns.

We propose that the co-occurrence of fish and insects within this interval of the T-OAE is related to environmental conditions during the T-OAE. A possible scenario is that shoals of fish were forced into shallower, near shore waters during intense de-oxygenation events (jubilee), where they died and became a food source for the higher trophic and predatory insects that are abundant in the Alderton Hill assemblage.

Our results indicate that the fossil insects from Alderton Hill represent a well-preserved and diverse assemblage that provides a useful insight into the palaeoentomofauna at temperate latitudes during the Toarcian. Comparison with the coeval assemblage of Strawberry Bank shows that the Alderton Hill insect assemblage can currently be regarded as the best-preserved and most representative record of Toarcian insects in the UK.

Acknowledgements

This research was supported by NERC CENTA (Central England NERC Training Alliance) PhD studentship to ES [grant number NE/S007350/1]. The authors acknowledge the following museums and institutions for allowing access and facilitating the examination of the Alderton Hill insect material and/or for providing relevant specimen information and photographs included within this publication: BGS (Simon Harris and Paul Shepherd); BRSMG (Deborah

Hutchinson); CHAGM (Benedict Sayers and Eleanor Edwardes); CAMSM (Elizabeth Harper and Matt Riley); NHMUK (Rich Howard); WARMS (Jon Radley). Further thanks is extended to Ulysses Thomson (né Richard Kelly) for his assistance and knowledge on collections of Lower Jurassic insect material at the beginning of this project. Matt Williams (Bath Royal Literary and Scientific Institution) and Andrew Ross (National Museum of Scotland) very kindly provided their database of the Strawberry Bank material at TTNCM, which was of great assistance in the compilation of our own extended database for Strawberry Bank. Matthew Butler is thanked for assisting ES in locating, cataloguing and photographing the associated fauna of Alderton Hill within the collections of NHMUK.

No permits were required for the described study, which complied with all relevant regulations.

References

1. IPCC. Climate Change 2022: Impacts, Adaptation, and Vulnerability. Contribution of Working Group II to the Sixth Assessment Report of the Intergovernmental Panel on Climate Change [Pörtner HO, Roberts DC, Tignor M, Poloczanska ES, Mintenbeck K, Alegría A, et al. (eds.)]. Cambridge UK: Cambridge University Press, 2022.

2. Larson EL, Tinghitella RM, Taylor SA. Insect Hybridization and Climate Change. Frontiers in Ecology and Evolution. 2019; 7: 1-11. doi:10.3389/fevo.2019.00348

3. Harvey JA, Tougeron K, Gols R, Heinen R, Abarca M, Abram PK, et al. Scientists' warning on climate change and insects. Ecological monographs. 2023; 93: 1-37. doi:10.1002/ecm.1553

4. Bale JS, Masters GJ, Hodkinson ID, Awmack C, Bezemer TM, Brown VK, et al. Herbivory in global climate change research: direct effects of rising temperature on insect herbivores. Global Change Biology. 2002; 8: 1-6. doi:10.1046/j.1365-2486.2002.00451.x

5. Wilson RJ, Maclean IM. Recent evidence for the climate change threat to Lepidoptera and other insects. Journal of Insect Conservation. 2011; 15: 259-68. doi:10.1007/s10841-010-9342-y

6. García-Robledo C, Kuprewicz EK, Staines CL, Erwin TL, Kress WJ. Limited tolerance by insects to high temperatures across tropical elevational gradients and the implications of global warming for extinction. Proceedings of the National Academy of Sciences. 2016; 113: 680-685. doi:10.1073/pnas.1507681113

7. Wagner DL. Insect declines in the Anthropocene. Annual review of entomology. 2020; 65: 457-480. doi:10.1146/annurev-ento-011019-025151

8. Andrew NR, Hill SJ, Binns M, Bahar MH, Ridley EV, Jung MP, et al. Assessing insect responses to climate change: What are we testing for? Where should we be heading?. PeerJ. 2013; 1:e11. doi:10.7717/peerj.11

9. Lehmann P, Ammunét T, Barton M, Battisti A, Eigenbrode SD, Jepsen JU, et al. Complex responses of global insect pests to climate warming. Frontiers in Ecology and the Environment. 2020; 18:141-50. doi:10.1002/fee.2160

10. Kemp DB, Coe AL, Cohen AS, Schwark L. Astronomical pacing of methane release in the Early Jurassic period. Nature. 2005; 437:396-399. doi:10.1038/nature04037

11. Kemp DB, Coe AL, Cohen AS, Weedon GP. Astronomical forcing and chronology of the early Toarcian (Early Jurassic) oceanic anoxic event in Yorkshire, UK. Paleoceanography. 2011; 26. doi:10.1029/2011PA002122

12. Hesselbo SP, Jenkyns HC, Duarte LV, Oliveira LC. Carbon-isotope record of the Early Jurassic (Toarcian) Oceanic Anoxic Event from fossil wood and marine carbonate (Lusitanian Basin, Portugal). Earth and Planetary Science Letters. 2007; 253: 455-470. doi:10.1016/j.epsl.2006.11.009

13. Hermoso M, Le Callonnec L, Minoletti F, Renard M, Hesselbo SP. Expression of the Early Toarcian negative carbon-isotope excursion in separated carbonate microfractions (Jurassic, Paris Basin). Earth and planetary science letters. 2009; 277:194-203. doi:10.1016/j.epsl.2008.10.013

14. Hermoso M, Minoletti F, Rickaby RE, Hesselbo SP, Baudin F, Jenkyns HC. Dynamics of a stepped carbon-isotope excursion: Ultra high-resolution study of Early Toarcian environmental change. Earth and Planetary Science Letters. 2012; 319: 45-54. doi:10.1016/j.epsl.2011.12.021

15. McArthur JM, Donovan DT, Thirlwall MF, Fouke BW, Mattey D. Strontium isotope profile of the early Toarcian (Jurassic) oceanic anoxic event, the duration of ammonite biozones, and belemnite palaeotemperatures. Earth and Planetary Science Letters. 2000; 179: 269-285. doi:10.1016/S0012-821X(00)00111-4

16. Bailey TR, Rosenthal Y, McArthur JM, Van de Schootbrugge B, Thirlwall MF. Paleoceanographic changes of the Late Pliensbachian–Early Toarcian interval: a possible link to the genesis of an Oceanic Anoxic Event. Earth and Planetary Science Letters. 2003; 212: 307-320. doi:10.1016/S0012-821X(03)00278-4

17. Rosales I, Quesada S, Robles S. Paleotemperature variations of Early Jurassic seawater recorded in geochemical trends of belemnites from the Basque–Cantabrian basin, northern Spain. Palaeogeography, Palaeoclimatology, Palaeoecology. 2004; 203: 253-275. doi:10.1016/S0031-0182(03)00686-2

18. Van de Schootbrugge B, McArthur JM, Bailey TR, Rosenthal Y, Wright JD, Miller KG. Toarcian oceanic anoxic event: An assessment of global causes using belemnite C isotope records. Paleoceanography. 2005; 20: 1-10. doi:10.1029/2004PA001102

19. Gómez JJ, Goy A, Canales ML. Seawater temperature and carbon isotope variations in belemnites linked to mass extinction during the Toarcian (Early Jurassic) in Central and

Northern Spain. Comparison with other European sections. Palaeogeography, Palaeoclimatology, Palaeoecology. 2008; 258: 28-58. doi:10.1016/j.palaeo.2007.11.005

20. Suan G, Mattioli E, Pittet B, Mailliot S, Lécuyer C. Evidence for major environmental perturbation prior to and during the Toarcian (Early Jurassic) oceanic anoxic event from the Lusitanian Basin, Portugal. Paleoceanography. 2008; 23: 1-14. doi:10.1029/2007PA001459

21. Suan G, Mattioli E, Pittet B, Lécuyer C, Suchéras-Marx B, Duarte LV, et al. Secular environmental precursors to Early Toarcian (Jurassic) extreme climate changes. Earth and Planetary Science Letters. 2010; 290: 448-58. doi:10.1016/j.epsl.2009.12.047

22. Dera G, Pucéat E, Pellenard P, Neige P, Delsate D, Joachimski MM, et al. Water mass exchange and variations in seawater temperature in the NW Tethys during the Early Jurassic: evidence from neodymium and oxygen isotopes of fish teeth and belemnites. Earth and Planetary Science Letters. 2009; 286: 198-207. doi:10.1016/j.epsl.2009.06.027

23. Gómez JJ, Arias C. Rapid warming and ostracods mass extinction at the Lower Toarcian (Jurassic) of central Spain. Marine Micropaleontology. 2010; 74: 119-135. doi:10.1016/j.marmicro.2010.02.001

24. Ullmann CV, Thibault N, Ruhl M, Hesselbo SP, Korte C. Effect of a Jurassic oceanic anoxic event on belemnite ecology and evolution. Proceedings of the National Academy of Sciences. 2014; 111: 10073-10076. doi:10.1073/pnas.1320156111

25. Cohen AS, Coe AL, Harding SM, Schwark L. Osmium isotope evidence for the regulation of atmospheric CO2 by continental weathering. Geology. 2004; 32: 157-160. doi:10.1130/G20158.1

26. Waltham D, Gröcke DR. Non-uniqueness and interpretation of the seawater 87Sr/86Sr curve. Geochimica et Cosmochimica Acta. 2006; 70: 384-394. doi:10.1016/j.gca.2005.09.014

27. Pearce CR, Cohen AS, Coe AL, Burton KW. Molybdenum isotope evidence for global ocean anoxia coupled with perturbations to the carbon cycle during the Early Jurassic. Geology. 2008; 36: 231-234. doi:10.1130/G24446A.1

28. McElwain JC, Wade-Murphy J, Hesselbo SP. Changes in carbon dioxide during an oceanic anoxic event linked to intrusion into Gondwana coals. Nature. 2005; 435: 479-82. doi:10.1038/nature03618

29. Caswell BA, Coe AL, Cohen AS. New range data for marine invertebrate species across the early Toarcian (Early Jurassic) mass extinction. Journal of the Geological Society. 2009; 166: 859-72. doi:10.1144/0016-76492008-0831

30. Little CT, Benton MJ. Early Jurassic mass extinction: a global long-term event. Geology. 1995; 23: 495-498. doi:10.1130/0091-7613(1995)023<0495:EJMEAG>2.3.CO;2

31. Nikitenko BL, Reolid M, Glinskikh L. Ecostratigraphy of benthic foraminifera for interpreting Arctic record of Early Toarcian biotic crisis (Northern Siberia, Russia). Palaeogeography, Palaeoclimatology, Palaeoecology. 2013; 376: 200-212. doi:10.1016/j.palaeo.2013.03.003

32. Caswell BA, Coe AL. The impact of anoxia on pelagic macrofauna during the Toarcian Oceanic Anoxic Event (Early Jurassic). Proceedings of the Geologists' Association. 2014; 125: 383-391. doi:10.1016/j.pgeola.2014.06.001

33. Caswell BA, Coe AL. Primary productivity controls on opportunistic bivalves during Early Jurassic oceanic deoxygenation. Geology. 2013; 41: 1163-1166. doi:10.1130/G34819.1

34. Caswell BA, Dawn SJ. Recovery of benthic communities following the Toarcian oceanic anoxic event in the Cleveland Basin, UK. Palaeogeography, Palaeoclimatology, Palaeoecology. 2019; 521: 114-126. doi:10.1016/j.palaeo.2019.02.014

35. Caswell BA, Frid CL. Marine ecosystem resilience during extreme deoxygenation: the Early Jurassic oceanic anoxic event. Oecologia. 2017; 183:275-90. doi:10.1007/s00442-016-3747-6

36. Piazza V, Duarte LV, Renaudie J, Aberhan M. Reductions in body size of benthic macroinvertebrates as a precursor of the early Toarcian (Early Jurassic) extinction event in the Lusitanian Basin, Portugal. Paleobiology. 2019; 45: 296-316. doi:10.1017/pab.2019.11

37. Ros-Franch S, Echevarría J, Damborenea SE, Manceñido MO, Jenkyns HC, Al-Suwaidi A, Hesselbo SP, et al. Population response during an oceanic anoxic event: the case of *Posidonotis* (Bivalvia) from the Lower Jurassic of the Neuquen Basin, Argentina. Palaeogeography, Palaeoclimatology, Palaeoecology. 2019; 525: 57-67. doi:10.1016/j.palaeo.2019.04.009

38. Caswell BA, Herringshaw L. Marine bioturbation collapse during Early Jurassic deoxygenation: implications for post-extinction marine ecosystem functioning. Geological Society, London, Special Publications. 2023; 529: 529-2022. doi:10.1144/SP529-2022-226

39. Slater SM, Twitchett RJ, Danise S, Vajda V. Substantial vegetation response to Early Jurassic global warming with impacts on oceanic anoxia. Nature Geoscience. 2019; 12: 462-467. doi:10.1038/s41561-019-0349-z

40. Reolid M, Ruebsam W, Benton MJ. Impact of the Jenkyns Event (early Toarcian) on dinosaurs: Comparison with the Triassic/Jurassic transition. Earth-Science Reviews. 2022: 104196: 1-24. doi:10.1016/j.earscirev.2022.104196

41. Szwedo J. The Coleorrhyncha (Insecta: Hemiptera) of the European Jurassic, with a description of a new genus from the Toarcian of Luxembourg. Volumina Jurassica. 2011; 9: 3-20.

42. Jarzembowski EA, Palmer D. Mesozoic fossil arthropods. In: Fossil arthropods of Great Britain. Geological Conservation Review Series: Joint Nature Conservation Committee, Peterborough; 2010. pp. 294.

43. Williams M, Benton MJ, Ross A. The Strawberry Bank Lagerstätte reveals insights into early Jurassic life. Journal of the Geological Society. 2015; 172: 683-692. doi:10.1144/jgs2014-144

44. Kelly RS, Ross AJ, Jarzembowski EA. Earwigs (Dermaptera) from the Mesozoic of England and Australia, described from isolated tegmina, including the first species to be named from the Triassic. Earth and Environmental Science Transactions of the Royal Society of Edinburgh. 2016; 107: 129-143. doi:10.1017/S1755691017000329

45. Thomson U [né Kelly, RS], Ross AJ, Coram RA. A review of necrotauliids from the Triassic/Jurassic of England (Trichoptera: Necrotauliidae). Psyche: A Journal of Entomology. 2018; 1-12. doi:10.1155/2018/6706120

46. Ansorge J. Insekten aus dem oberen Lias von Gimmen (Vorpommern, Norddeutschland). Paläontologische Abhandlungen. 1996; 2: 1-132.

47. Bode A. Orthoptera und Neuroptera aus dem oberen Lias von Braunschweig. Jahrbuch der preussischen geologischen Landesanstalt. 1905; 25: 218-245.

48. Bode A. Die Insektenfauna des ostniedersächsischen oberen Lias. Palaeontographica A. 1953; 103: 1-375.

49. Kopeć K, Soszyńska-Maj A, Gehler A, Ansorge J, Krzemiński W. Mecoptera and Diptera from the early Toarcian (Early Jurassic) deposits of Wolfsburg–Große Kley (Lower Saxony, Germany). Earth and Environmental Science Transactions of the Royal Society of Edinburgh. 2017a; 107: 163-171. doi:10.1017/S1755691017000226

50. Brachert T. Makrofossilführung der "Siemensi-Geoden" (Mittlerer Lias Epsilon, Unteres Toarcium) von KerkhofeniOberpfalz (Bayern): Neue Insekten- und Pflanzenfunde. Geologische Blätter NO-Bayern. 1987; 37: 217-240.

51. Ansorge J. Insects from the lower Toarcian of middle Europe and England. Acta Zoologica Cracoviensia. 2003; 46: 291-310.

52. Rasnitsyn AP, Ansorge J, Zessin W. New hymenopterous insects (Insecta: Hymenoptera) from the Lower Toarcian (Lower Jurassic) of Germany. Neues Jahrbuch für Geologie und Paläontologie-Abhandlungen. 2003; 10: 321-342. doi:10.1127/njgpa/227/2003/321

53. Henrotay M, Nel A, Jarzembowski EA. New protomyrmeleontid damselflies from the Triassic of Australia and the Liassic of Luxembourg, with the description of *Tillyardomyrmeleon petermilleri* gen. nov. & spec. nov.(Archizygoptera: Protomyrmeleontidae). Odonatologica. 1997; 26: 395-404.

54. Henrotay M, Marques D, Paicheler JC, Gall JC, Nel A. Le Toarcien inférieur des régions de Bascharage et de Bettembourg (grand-duché du Luxembourg): évidences paléontologiques et sédimentologiques d'environnements restreints proches de l'émersion. Geodiversitas. 1998; 20: 263-84.

55. Nel A, Pertulevicius JF, Henrotay M. New Early Jurassic sawflies from Luxembourg: the oldest record of Tenthredinoidea [Hymenoptera: Symphyta]. Acta Palaeontologica Polonica. 2004; 49: 283-288.

56. Szwedo J, Weis R, Nel A. A bizarre sternorrhynchan wing from the Lower Jurassic of Luxembourg (Hemiptera: Sternorrhyncha: Pincombeomorpha?). Historical Biology. 2019; 31: 806-12. doi:10.1080/08912963.2017.1395423

57. Nel A, Weis R. A new early Jurassic damselfly from the Grand Duchy of Luxembourg (Odonata: Campterophlebiidae). Alcheringa: An Australasian Journal of Palaeontology. 2017; 41: 378-82. doi:10.1080/03115518.2017.1289417

58. Bechly G. First record and a new species of the fossil dragonfly genus Proinogomphus (Odonata: Liassogomphidae) from the Early Jurassic of Bascharage in the Grand Duchy of Luxembourg. Zootaxa. 2018; 4450: 108-14. doi:10.11646/zootaxa.4450.1.7

59. Etter W, Kuhn O. An articulated dragonfly (Insecta, Odonata) from the Upper Liassic Posidonia Shale of northern Switzerland. Palaeontology. 2000; 43: 967-77. doi:10.1111/1475-4983.00157

60. Delsate D, Henrotay M, Godefroit P. Présence d'insectes dans le Toarcien inférieur de la Belgique. Bulletin de la Société belge de Géologie. 1992; 100: 147-53.

61. Buckman J. On the occurrence of the remains of insects in the Upper Lias of the county of Gloucester. Proceedings of the Geological Society, London. 1843; 4: 211-212.

62. Buckman J. Remarks on *Libellula Brodiei* (Buckman), a fossil insect from the upper Lias of Dumbleton, Gloucestershire. Annals and Magazine of Natural History. 1853; 12: 436-438.

63. Brodie PB. A history of the fossil insects in the Secondary rocks of England. John van Vaarst, London. 1845. pp. 130.

64. Brodie PB. Notice on the discovery of a dragon-fly and a new species of *Leptolepis* in the Upper Lias near Cheltenham, with a few remarks on that formation in Gloucestershire. Quarterly Journal of the Geological Society. 1849; 5: 31-7.

65. Simms MJ. The Severn Basin. In: Jenkyns H, Simms MJ, Chidlaw N, Morton N, Page KN. British Lower Jurassic Stratigraphy. Geological Conservation Review Series: Joint Nature Conservation Committee, Peterborough; 2004. pp. 458.

66. Westwood JO. Contributions to fossil entomology. Quarterly Journal of the Geological Society. 1854; 10: 378-96.

67. Geinitz FE. Über die Fauna des Dobbertiner Lias Zeitschrift der deutschen geologischen Gesellschaft. 1884; 36: 566-583.

68. Scudder S. A review of Mesozoic cockroaches. Memoirs of the Boston Society of Natural History. 1886; 3: 439-485.

69. Whalley PES. Mesozoic Neuroptera and Raphidioptera (Insecta) in Britain. Bulletin British Museum Natural History, Geol. Ser. 1988; 44: 45-63.

70. Tillyard RJ. Fossil Insects, No.1: The British Liassic dragonflies. British Museum (Nat. Hist.), London; 1925. pp 40.

71. Tillyard RJ. Fossil Insects, No. 3: The Panorpoid complex in the British Rhaetic and Lias. British Museum (Nat. Hist.), London; 1933. pp 79.

72. Zeuner FE. Descriptions of new genera and species of fossil Saltatoria (Orthoptera). In: Proceedings of the Royal Entomological Society of London. Series B, Taxonomy. Blackwell Publishing Ltd, Oxford, UK; 1937. pp. 154-159.

73. Zeuner FE. Fossil Orthoptera Ensifera. British Museum (Nat. Hist.), London; 1939.

74. Nel A, Martinez-Delclos X, Paicheler JC, Henrota YM. Les "Anisozygoptera" fossiles Phylogenie et classification (Odonata). Martinia, Numero Hors Serie. 1993; 3: 1-311.

75. Jouault C, Tischlinger H, Henrotay M, Nel A. Wing coloration patterns in the Early Jurassic dragonflies as potential indicator of increasing predation pressure from insectivorous reptiles. Palaeoentomology. 2022; 5: 305-318. doi:10.11646/palaeoentomology.5.4.3

76. Vršanský P, Ansorge J. Lower Jurassic cockroaches (Insecta: Blattaria) from Germany and England. African Invertebrates. 2007; 48: 103-26.

77. Kopeć K, Krzemiński W, Skowron K, Coram R. The genera Architipula Handlirsch, 1906 and Grimmenia Krzemiński and Zessin, 1990 (Diptera: Limoniidae) from the Lower Jurassic of England. Palaeontologia Electronica. 2017b; 20: 1-7. doi:10.26879/637

78. Ansorge J. Revision of the “Trichoptera” described by Geinitz and Handlirsch from the Lower Toarcian of Dobbertin (Germany) based on new material. Nova Suppl Entomol. 2002; 15: 55-74.

79. Tomes RF. On the occurrence of two species of Madreporia in the Upper Lias of Gloucestershire. Geological Magazine, Decade 3. 1886; 3: 107–111.

80. Richardson L. The Country around Moreton-in-Marsh, Memoir of the Geological Survey of Great Britain, Sheet 217 (England and Wales); 1929. pp. 162.

81. Moore C. On the palaeontology of the Middle and Upper Lias. Proceedings of the Somersetshire Archaeological and Natural History Society. 1853; 3: 61–76.

82. Moore C. On the Middle and Upper Lias of the South West of England. Proceedings of Somerset Archaeological and Natural History Society. 1866; 13: 119–244.

83. Caine H, Benton MJ. Ichthyosauria from the Upper Lias of Strawberry Bank, England. Palaeontology. 2011; 54: 1069–1093. doi:10.1111/j.1475-4983.2011.01093.x

84. Srdic A, Beardmore S, Lomax DR. A rediscovered Lower Jurassic ichthyosaur skeleton possibly from the Strawberry Bank Lagerstätte, Somerset, UK. Historical Biology. 2021; 33: 814-822. doi:/10.1080/08912963.2019.1663840

85. Jamison-Todd S, Moon BC, Rowe AJ, Williams M, Benton MJ. Dietary niche partitioning in Early Jurassic ichthyosaurs from Strawberry Bank. Journal of Anatomy. 2022; 241 :1409-1423. doi:10.1111/joa.13744

86. Pierce SE, Benton MJ. Pelagosaurus typus Bronn, 1841 (Mesoeucrocodylia: Thalattosuchia) from the Upper Lias (Toarcian, Lower Jurassic) of Somerset, England. Journal of Vertebrate Paleontology. 2006; 26: 621-635. doi:10.1671/0272-4634(2006)26[621:PTBMTF]2.0.CO;2

87. Cawley JJ, Kriwet J, Klug S, Benton MJ. The stem group teleost Pachycormus (Pachycormiformes: Pachycormidae) from the Upper Lias (Lower Jurassic) of Strawberry Bank, UK. PalZ. 2019; 93: 285-302. doi:10.1007/s12542-018-0431-7

88. Sinha S, Muscente AD, Schiffbauer JD, Williams M, Schweigert G, Martindale RC. Global controls on phosphatization of fossils during the Toarcian Oceanic Anoxic Event. Scientific Reports. 2021; 11: 1-13. doi:10.1038/s41598-021-03482-7

89. Audo D, Williams M, Charbonnier S, Schweigert G. Gabaleryon, a new genus of widespread early Toarcian polychelidan lobsters. Journal of Systematic Palaeontology. 2017; 15: 205-222. doi:10.1080/14772019.2016.1167786

90. Little C, Gale A, Williams M, Hammer Ø, Fernandez V. Bivalve-barnacle pseudoplanktonic colonisation of wood from the Toarcian, Lower Jurassic, Strawberry Bank Lagerstätte, Somerset UK. Acta Palaeontologica Polonica. 2023; 24; 133-142. doi:10.4202/app.01018.2022

91. Natural England. Designated Sites View: Alderton Hill Quarry SSSI. 1997. Available from: https://designatedsites.naturalengland.org.uk/SiteDetail.aspx?SiteCode=S2000150&SiteName=alderton&countyCode=&responsiblePerson=&SeaArea=&IFCAArea=

92. Scotese, C.R. Paleomap Project. www.scotese.com. 2013.

93. Fantasia A, Föllmi KB, Adatte T, Spangenberg JE, Mattioli E. Expression of the Toarcian Oceanic Anoxic Event: new insights from a Swiss transect. Sedimentology. 2019; 66:262-84. doi:10.1111/sed.12527

94. Caswell BA, Coe AL. A high-resolution shallow marine record of the Toarcian (Early Jurassic) Oceanic Anoxic Event from the East Midlands Shelf, UK. Palaeogeography, Palaeoclimatology, Palaeoecology. 2012; 365: 124-135. doi:10.1016/j.palaeo.2012.09.021

95. Ruebsam W, Al-Husseini M. Calibrating the Early Toarcian (Early Jurassic) with stratigraphic black holes (SBH). Gondwana Research. 2020; 82:317-36. doi:10.1016/j.gr.2020.01.011

96. Ansorge J, Makarkin VN. The oldest giant lacewings (Neuroptera: Kalligrammatidae) from the Lower Jurassic of Germany. Palaeoworld. 2021; 30: 296-310. doi:10.1016/j.palwor.2020.07.001

97. Moxon C. Illustrations of the Characteristic Fossils of British Strata, H. Bailliere, London. 1841; 46 pp.

98. Howarth MK. The ammonite family Hildoceratidae in the Lower Jurassic of Britain. Monographs of the Palaeontographical Society. 1992; 146: 107-192.

99. Crick GC. On the proostracum of a belemnite from the Upper Lias of Alderton, Gloucestershire. Proceedings of the Malacological Society of London. 1896; 2: 117–119.

100. Crick, GC. On some dibranchiate cephalopoda from the Upper Lias of Gloucestershire. Proceedings of the Cotteswold Naturalists' Field Club. 1922; 20: 249–256.

101. Doyle P. The British Toarcian (Lower Jurassic) belemnites. Monographs of the Palaeontographical Society. 1990-1992; 145: 50-75.

102. Woodward H. On a New Species of Eryon from the Upper Lias, Dumbleton Hill. Geological Magazine. 1911; 8: 307-311.

103. Woods, H. A Monograph of the fossil macrurous Crustacea of England. Monograph of the Palaeontographical Society. Palaeontographical Society, London; 1925–1931.

104. Delsate D. Actinoptérygiens du Toarcien inférieur du Grand Duché de Luxembourg: présence de Leptolepis normandica NYBELIN 1962 (Téléostéen) avec otolithes in situ. Travaux scientifiques du Musée national d'histoire naturelle de Luxembourg. 1997; 27: 105-129.

105. Bronn H. Uber zwei fossile Fischarten: Cyprinus coryphaenoides und Tetragonolepis semicinctus aus dem Gryphitenkalke bei Donaueschingen. Jahrbuch fur Mineralogie, Geognosie und Petrefaktenkunde. 1830; 13-40.

106. de Blainville HD. Poissons fossiles. Nouvelle Dictionnaire d'Histoire Naturelle. 1818; 27: 310-395.

107. Münster G. Die schalenlosen Cephalopoden im unteren Jura, den Lias-Schiefern von Franken und Schwaben. pp. 57-77 *in* Munster G, Meyer HV, Wagner R. (Eds.), Beiträge zur Petrefacten-Kunde mit xviii nach der Naturgezeichneten Tafeln. 1843. Buchner, Bayreuth.

108. Sowerby J de C. The Mineral Conchology of Great Britain. Volume V, London. 1823-1825.

109. Bruguière JG. Histoire Naturelle des vers. Enciclopeédie Méthodique. 1789; 6: 1-344.

110. Zieten CH. von. Die Versteinerungen Württembergs oder natur getreue Abbildungen der in den vollständigsten Sammlungen, namentlich der in dem Kabinett des Oberamts-Arzt D. Hartmann befindlichen Petrefacten, mit Angabe der Gebirgs-Formationen, in welchen dieselben vorkommen und der Fundorte. 1830–1833; 102 pp. Verlag & Lithographie der Expedition des Werkes unserer Zeit, Stuttgart.

111. Bode A. *Chondroteuthis wunnenbergi* ngn sp., eine neue Belemnoideenform, in gunstiger Erhaltung. Sonderabdruck aus dem 25. Jahresbericht des Niedersachsischen geologischen Vereins zu Hannover (Geologische Abteilung der Naturhistorischen Gesellschaft zu Hannover). 1933; 25: 33-66.

112. Boomer I, Copestake P, Page K, Huxtable J, Loy T, Bown P, et al. Biotic and stable-isotope characterization of the Toarcian Ocean Anoxic Event through a carbonate–clastic sequence from Somerset, UK. Special Publications. 2021; 514: 239-68. doi:10.1144/SP514-2020-263

113. Wright T. Report on Miss Holland’s collection of Lias fossils. Proceedings of the Cotteswold Naturalists’ Field Club. 1863; 3: 153–156.

114. Kelly RS. Effects of environmental perturbation during the Late Triassic on the taxic diversity of British insects. Doctoral Thesis, University of Bristol. 2018. Available from: https://ethos.bl.uk/OrderDetails.do?uin=uk.bl.ethos.787662

115. Westwood JO. A History of the Fossil Insects in the Secondary Rocks of England Accompanied by a Particular Account of the Strata in which they Occur, and of the Circumstances Connected with their Preservation. 1845; 1-130.

116. Bengtson P. Open nomenclature. Palaeontology. 1988; 31:223-7.

117. Grimaldi D, Engel MS. Evolution of the Insects. Cambridge UK: Cambridge University Press; 2005.

118. Geinitz FE. Beitrag zur Geologie Mecklenburgs. Archiv des Vereins der Freunde der Naturgeschichte in Mecklenburg. 1888; 41:143-216.

119. De Geer C. Mémoires pour Servir à l'Histoire des Insectes, Tome Troisième. 1773; 1-696.

120. Latreille PA. Considérations générales sur l'ordre naturel des animaux composant les classes des Crustacés, des Arachnides et des Insectes; avec un tableau méthodique de leurs genres, disposés en familles. – Considérations générales sur l'ordre naturel des animaux composant les classes des Crustacés, des Arachnides et des Insectes. 1810; 1–444

121. Handlirsch A. Die fossilen Insekten und die Phylogenie der rezenten Formen. Engelmann, Leipzig. 1906-1908.

122. Heer O. Dobbertin's Jurassic in Mecklenburg and its fossils. Journal of the German Geological Society. 1880; 32: 510–535.

123. Olivier GA. Encyclopédie Méthodique. Histoire naturelle, Tome Quatrieme, Insectes. 1789; 1-331.

124. Geinitz FE. Der Jura von Dobbertin in Mecklenburg und seine Versteinerungen. Zeitschrift der Deutschen Geologischen Gesellschaft. 1880; 32:510-535.

125. Linnaeus C. Systema Naturae per Regna Tria Naturae, Secundum Classes, Ordines, Genera, Species, cum Characteribus, Differentiis, Synonymis, Locis. Tomus I. Editio decima, reformata. Holmiae: Laurentii Salvii. 1758; 1-4: 1-824.

126. Wang B, Zhang H, Jarzembowski EA, Fang Y, Zheng D. Taphonomic variability of fossil insects: a biostratinomic study of Palaeontinidae and Tettigarctidae (Insecta: Hemiptera) from the Jurassic Daohugou Lagerstätte. Palaios. 2013; 28: 233-242. doi:10.2110/palo.2012.p12-045r

127. Hammer Ø, Harper DA, Ryan PD. PAST: Paleontological statistics software package for education and data analysis. Palaeontologia Electronica. 2001; 4: 1-9.

128. Brauer F. Systematisch-zoologische Studien. Sitzungsberichte der Kaiserlichen Akademie der Wissenschaften, Mathematisch-Naturwissenschaftliche Classe. 1885; 91: 237–413.

129. Hyatt A, Arms JM. Ephemeroptera. Guides for Science Teaching. 1890; 8: 69-72.

130. Fabricius JC. Entomologia systematica emendata et aucta: secundum classes, ordines, genera, species, adjectis synonymis, locis, observationibus, descriptionibus. 1793.

131. Needham JG, Anthony MH. The skewness of the thorax in the Odonata. Journal of the New York Entomological Society. 1903; 11: 117–125.

132. Westwood JO. In Notice on the discovery of a dragon-fly and a new species of Leptolepis in the upper Lias near Cheltenham, with a few remarks on that formation in Gloucestershire. Quarterly Journal of the Geological Society. 1849; 5:31-37.

133. Handlirsch A. Neue Untersuchungen über die fossilen Insekten mit Ergänzungen und Nachträgen sowie Ausblicken auf phylogenetische, palaeogeographische und allgemein

biologische Probleme. II Teil. Annalen des Naturhistorischen Museums in Wien. 1939; 49:1-240.

134. Ansorge J. *Heterophlebia buckmani* (Brodle 1845) (Odonata: "Anisozygoptera") - das erste Insekt aus dem untertoarcischen Posidonienschiefer von Holzmaden (Württemberg, SW Deutschland). Stuttgarter Beiträge zur Naturkunde, Serie B. 1999; 275: 1-9.

135. Tillyard RJ. Upper Permian insects of New South Wales. IV. The order Odonata. Proceedings of the Linnean Society of New South Wales. 1935; 60: 374–384.

136. Ansorge J. Insekten aus Liasgeoden der Ahrensburger Geschiebesippe–mit einem Ausblick auf lokale Anreicherungen von Liasgeoden in Mecklenburg-Vorpommern. Archiv für Geschiebekunde. 2004; 3:779-84.

137. Zessin W. Die Phylogenie der Protomyrmeleontidae unter Einbeziehung neuer oberliassischer Funde (Odonata: Archizygoptera sens, nov.). Odonatologica. 1991; 20: 97-126.

138. Nel A, Henrotay M. Les Protomyrmeleontidae (odonatoptera, odonata, Archizygoptera stat. rest.): état actuel des connaissances. Annales de paléontologie. 1992; 78:1-47.

139. Latreille PA. Crustacés, Arachnides et partie des Insects. In: Cuvier G (ed.) Le règne animal distribute d'après son organization, pour server de base à l'histoire naturelle des animaux, et d'introduction à l'anatomie compare. Deterville, Paris. 1829; pp. 1–39.

140. Vishnyakova VN. Novyye tarakany (Insecta: Blattodea) iz Verkhneyurskikh otlozheniy khrebta Karatau. *In* Voprosy Paleontologii Nasekomykh. Dokladyna 24m Ezhegodnom Chtenii Pamyati N.A.Kholodkovskogo; Narchuk, E.P., Ed.; Nauka, Leningrad: Saint Petersburg, Russia, 1973; pp. 64–77.

141. Vršanský P, Ansorge J. Systematic paleontology, in decreasing variability - from the Carboniferous to the present (Validated on independent lineages of Blattaria). Paleontological Journal. 2000; 34:374-379.

142. Zessin W. Locustopsis kruegeri n. sp.(Orthopteroida, Caelifera) aus dem oberen Lias von Schandelah bei Braunschweig (BRD). Zeitschrift für geologische Wissenschaften. 1983; 11: 905-10.

143. Giebel CG. Fauna der Vorwelt mit steter Breucksichtigung der lebenden Thiere. Brockhaus F. A., Leipzig. 1856; 551 pp.

144. Gorochov AV, Jarzembowski EA, Coram RA. Grasshoppers and crickets (Insecta: Orthoptera) from the Lower Cretaceous of southern England. Cretaceous Research. 2006; 27: 641-662. doi:10.1016/j.cretres.2006.03.007

145. Sharov AG. Filogeniya Orthopteroidnykh nasekomykn (Phylogeny of the orthoptera). Trudy Paleontologicheskogo Instituta, Akademii nauk SSSR. 1968; 118; 1e217 (in Russian).

146. Zessin W. Variabilität, Merkmalswandel und Phylogenie der Elcanidae im Jungpaläozoikum und Mesozoikum und die Phylogenie der Ensifera (Orthopteroida, Ensifera). Deutsche Entomologische Zeitschrift. 1987; 34:1-76.

147. Heer O. Die Urwelt der Schweiz. 1865; 1-622.

148. Heads S, Thuy B, Tamarri J. Two new species of Archelcana Sharov (Orthoptera: Elcanidae) from the Lower Jurassic of Luxembourg. Kentiana. 2022. doi:10.21900/j.kentiana.n1.1121

149. Cockerell TD. British fossil insects. Proceedings of the United States National Museum. 1915.

150. Duméril AMC. Zoologie Analytique, ou Méthode Naturelle de Classification des Animaux, Rendue plus Facile à l'Aide de Tableaux Synoptiques. 1805; 1-344.

151. Martynov AV. Jurassic fossil insects from Turkestan. 6. Homoptera and Psocoptera. Известия Российской академии наук. Серия математическая. 1926; 20:1349-66.

152. Becker-Migdisova EE. Nekotorye novye poluzhestkokrylye i senoedy. Paleontological Journal. 1962; 89-104.

153. Myers JG. The systematic position of the Peloridiidae as elucidated by a further study of the external anatomy of *Hemiodoecus leadi* China. Annals and Magazine of Natural History. 1929; 105: 282–294.

154. Popov YA. Historical development of true bugs of the infraorder Nepomorpha (Heteroptera). Trudy Paleontologicheskogo Instituta AN SSSR. 1971; 129: 1–230.

155. Popov YA, Wootton RJ. The upper liassic Heteroptera of Mecklenburg and Saxony. Systematic Entomology. 1977; 2: 333-351.

156. Engel MS. "Establishment of some clade names for Amphiesmenoptera (Insecta: Holometabola)". Novitates Paleoentomologicae. 2022; 24: 1–7. doi:10.17161/np.24.18498

157. Kristensen NP. Early evolution of the Trichoptera+Lepidoptera lineage: phylogeny and the ecological scenario. Mémoires Du Muséum D'histoire Naturelle. 1997; 173: 253–271.

158. Zhang W, Shih C, Ren D. Two new fossil caddisflies (Amphiesmenoptera: Trichoptera) from the Middle Jurassic of northeastern China. Alcheringa: An Australasian Journal of Palaeontology. 2016; 41: 22-29. doi:10.1080/03115518.2016.1170501

159. Pierwola AA, Grimaldi DA. First New World Necrotaulius reflects the Laurasian land masses (Insecta: Amphiesmenoptera: Necrotauliidae). Neues Jahrbuch für Geologie und Paläontologie-Abhandlungen. 2022; 16:37-50. doi:10.1127/njgpa/2022/1056

160. Packard AS. A new arrangement of the orders of insects. The American Naturalist. 1886; 20: 1-808.

161. Martynova OM. Mezozoyskie setchatokrylie (Neuroptera) i ikh znachenie dlya ponimaniya filogenii i sistematiki otryada. Akademiya Nauk SSSR, Trudy Paleontologicheskogo Instituta. 1949. 20:150-170.

162. Willmann R. Evolution und phylogenetisches System der Mecoptera (Insecta: Holometabola) Abh Senckb naturforsch Ges. 1989; 544:1–153.

163. Handlirsch A. Kapitel 7. Palaeontologie. In: C. Schröder. Handbuch der Entomologie; 1920-1921. pp. 304.

164. Willmann R. Mecopteren aus dem Lias von Niedersachsen (Insecta, Holometabola). Neues Jahrbuch für Geologie und Paläontologie-Monatshefte. 1984; 2:437-48.

165. Scudder SH. Notes on Mesozoic cockroaches. Proceedings of the Academy of Natural Sciences of Philadelphia. 1885; 105-115.

166. Ponomarenko AG. Upper Liassic neuropterans (Insecta) from Lower Saxony, Germany. Russian Entomological Journal. 1995; 4: 73–89.

167. Peng Y, Makarkin VN, Ren D. Diverse new Middle Jurassic Osmylopsychopidae (Neuroptera) from China shed light on the classification of psychopsoids. Journal of Systematic Palaeontology. 2016; 14: 261-95. doi:10.1080/14772019.2015.1042080

168. Khramov AV, Makarkin VN. New fossil Osmylopsychopidae (Neuroptera) from the Early/Middle Jurassic of Kyrgyzstan, Central Asia. Zootaxa. 2015; 4059:115-32. doi:10.11646/zootaxa.4059.1.6

169. Liu X, Ren D, Shih C, Wang Y. New Osmylopsychopid Taxa from the Middle Jurassic of Northeastern China (Neuroptera: Osmylopsychopidae). Insects. 2023; 14:484. doi:10.3390/insects14050484

170. Makarkin VN, Yang Q, Peng Y, Ren D. A comparative overview of the neuropteran assemblage of the Lower Cretaceous Yixian Formation (China), with description of a new genus of Psychopsidae (Insecta: Neuroptera). Cretaceous Research. 2012; 35: 57-68. doi:10.1016/j.cretres.2011.11.013

171. Khramov AV. Two new lacewings (Neuroptera) from the Upper Jurassic locality Shar-Teg (Mongolia). Paleontological Journal. 2011; 45: 174-8. doi:10.1134/S0031030111020080

172. Latreille PA Familles naturelles du regne animal, exposees succinctement et dans un ordre analytique, avecl'indication de leurs gentes. Pairs. 1825; 570 pp.

173. Speiser P. Diptera. 4. Orthorapha. Wissenschaftliche Ergebnisse der Schwedischen Zoologischen Expedition nach dem Kilimandjaro, dem Meru und den Umgebenden Massaisteppen Deutsch-Ostafrikas. 1909; 2:31-112.

174. Krzemiński W, Zessin W. The Lower Jurassic Limoniidae from Grimmen (GDR) (Dipt. Nematocera). Deutsche Entomologische Zeitschrift. 1990; 37:39-43. doi:10.1002/mmnd.19900370113

175. Kopeć K, Ansorge J, Soszyńska-Maj A, Krzemiński W. Revision of the genus Mesotipula Handlirsch, 1920 (Diptera, Limoniidae, Architipulinae) from the lower Jurassic of northeast Germany. Historical Biology. 2018; 32: 500-507. doi:10.1080/08912963.2018.1503257

176. Vishnyakova VN. Earwigs (Insecta, Forficulida) from the Upper Jurassic of the Karatau range. Paleontological Journal. 1980; 14:63-79.

177. Laporte FL. Histoire naturelle des animaux articulés, annélides, crustacés, arachnidés, myriapodes et insectes. Duménil, Paris. 1836; pp. 324.

178. Kiriakoff SG. A classification of the Lepidoptera and related groups with some remarks on taxonomy. Biologisch Jaarboek. 1948; 15:118–143.

179. El-Moursy A, El-Hawagry M, Abdeldayem M, Fadl H. Insect diversity in Zaranik protectorate, Northern Sinai, Egypt. Egyptian Journal of Natural History. 2001; 3: 62-80. doi:10.4314/ejnh.v3i1.30002

180. Rego C, Boieiro M, Vieira V, Borges PA. The biodiversity of terrestrial arthropods in Azores. Revista IDE@-SEA. 2015; 5: 1-24.

181. Karr JA, Clapham ME. Taphonomic biases in the insect fossil record: shifts in articulation over geologic time. Paleobiology. 2015; 41: 16-32. doi:10.1017/pab.2014.3

182. Acquah-Lamptey D, Kyerematen R, Owusu EO. Using odonates as markers of the environmental health of water and its land related ecotone. International Journal of Biodiversity and Conservation. 2013; 5: 761-769.

183. Popov YA, Shcherbakov DE. Origin and evolution of the Coleorrhyncha as shown by the fossil record. Studies on hemipteran phylogeny. 1996: 9-30. doi:10.4182/AMYC5234.1996.9

184. Mander L, McElwain JC. Toarcian land vegetation loss. Nature Geoscience. 2019; 12:405-6. doi:10.1038/s41561-019-0366-y

185. Goczał J, Beutel RG. Beetle elytra: evolution, modifications and biological functions. Biology Letters. 2023; 19:20220559. doi:10.1098/rsbl.2022.0559

186. Linz DM, Hu AW, Sitvarin MI, Tomoyasu Y. Functional value of elytra under various stresses in the red flour beetle, Tribolium castaneum. Scientific Reports. 2016; 6:34813. doi:10.1038/srep34813

187. Smith DM, Cook A, Nufio CR. How physical characteristics of beetles affect their fossil preservation. Palaios. 2006; 21: 305-310. doi:10.2110/palo.2004.p04-91

188. Martin SK. Hill River rediscovered: Early Jurassic insects of the Perth Basin, Western Australia. Alavesia. 2008; 2: 7-14.

189. Duncan IJ, Titchener F, Briggs DE. Decay and disarticulation of the cockroach: implications for preservation of the blattoids of Writhlington (Upper Carboniferous), UK. Palaios. 2003; 18: 256-265. doi:10.1669/0883-1351(2003)018<0256:DADOTC>2.0.CO;2

190. Martínez-Delclòs X, Briggs DE, Peñalver E. Taphonomy of insects in carbonates and amber. Palaeogeography, Palaeoclimatology, Palaeoecology. 2004; 203: 19-64. doi:10.1016/S0031-0182(03)00643-6

191. Coram RA, Jarzembowski EA. Immature Insect Assemblages from the Early Cretaceous (Purbeck/Wealden) of Southern England. Insects. 2021; 12:942. doi:10.3390/insects12100942

192. Chen J, Wang B, Engel MS, Wappler T, Jarzembowski EA, Zhang H, et al. Extreme adaptations for aquatic ectoparasitism in a Jurassic fly larva. Elife. 2014; 3:e02844. doi:10.7554/eLife.02844

193. Loesch H. Sporadic mass shoreward migrations of demersal fish and crustaceans in Mobile Bay, Alabama. Ecology. 1960; 41: 292-298. doi:10.2307/1930218

194. May EB. Extensive oxygen depletion in Mobile Bay, Alabama 1. Limnology and Oceanography. 1973; 18: 353-66. doi:10.4319/lo.1973.18.3.0353

195. Montiel D, Lamore A, Stewart J, Dimova N. Is submarine groundwater discharge (SGD) important for the historical fish kills and harmful algal bloom events of Mobile Bay? Estuaries and Coasts. 2019; 42: 470-493. doi:10.1007/s12237-018-0485-5

196. Meyer-Gutbrod E, Kui L, Miller R, Nishimoto M, Snook L, Love M. Moving on up: Vertical distribution shifts in rocky reef fish species during climate-driven decline in dissolved oxygen from 1995 to 2009. Global Change Biology. 2021; 27: 6280-6293. doi:10.1111/gcb.15821

197. Swaby, EJ, Coe AL, Ansorge J, Caswell BA, Hayward SAL, Mander L, Stevens LG, McArdle A. Data from: The fossil insect assemblage associated with the Toarcian (Lower Jurassic) Oceanic Anoxic Event from Alderton Hill, Gloucestershire, UK. 2023. Dryad Digital Repository. doi:10.5061/dryad.wh70rxwv0

Data availability statement

The datasets and photographs of the Alderton Hill and Strawberry Bank specimens are available on the Dryad Digital Repository [197].

[Dryad have made the dataset available for reviewers at: https://datadryad.org/stash/share/0g_D75vgw0G5e1dMfmQEEEMZYVPTKErCpD9b67Jr8rs]

Figure 1

Click here to access/download;Figure;Fig1.tif.tif

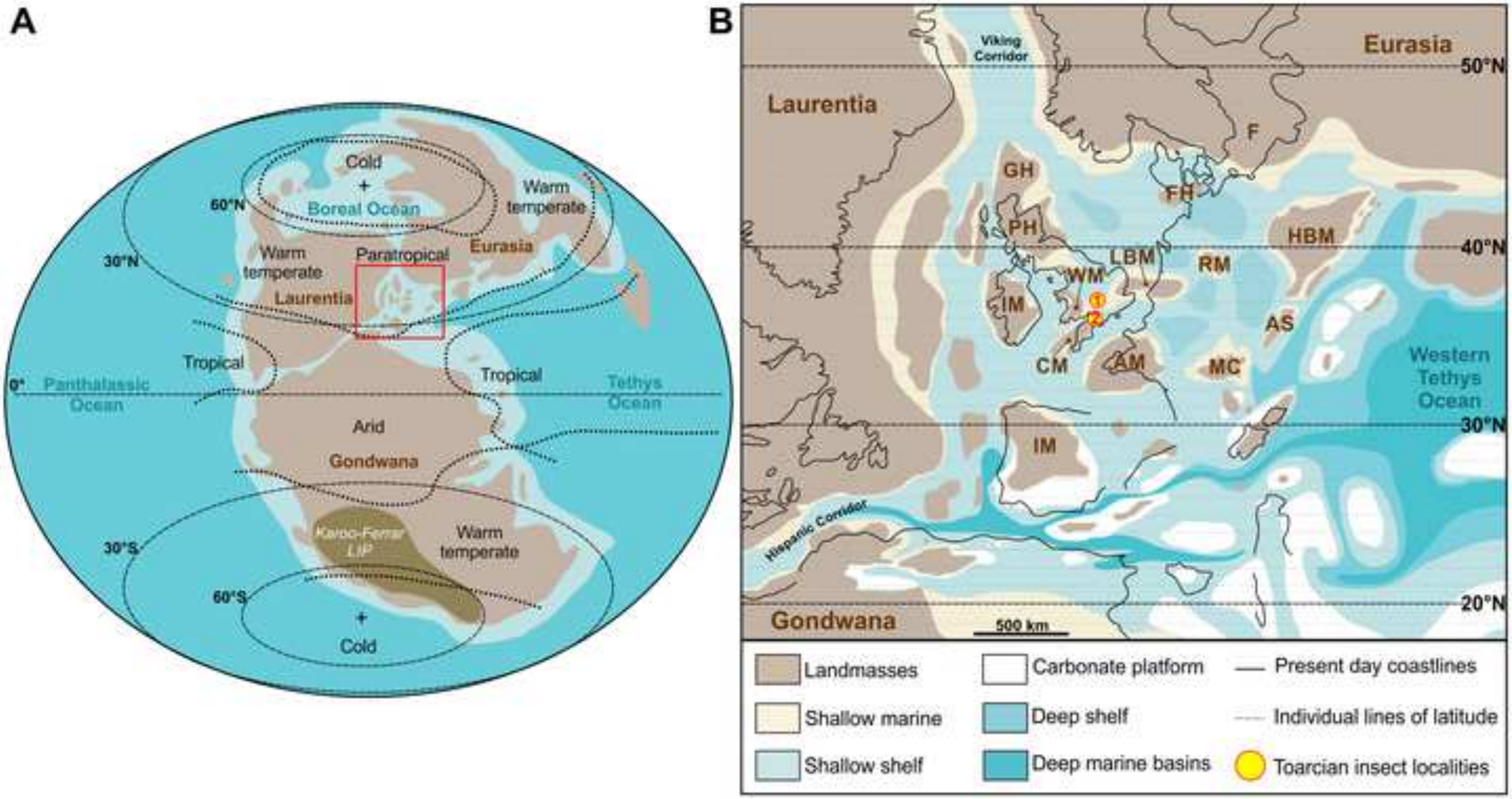

Figure 2 Click here to access/download;Figure;Fig2.tif.tif

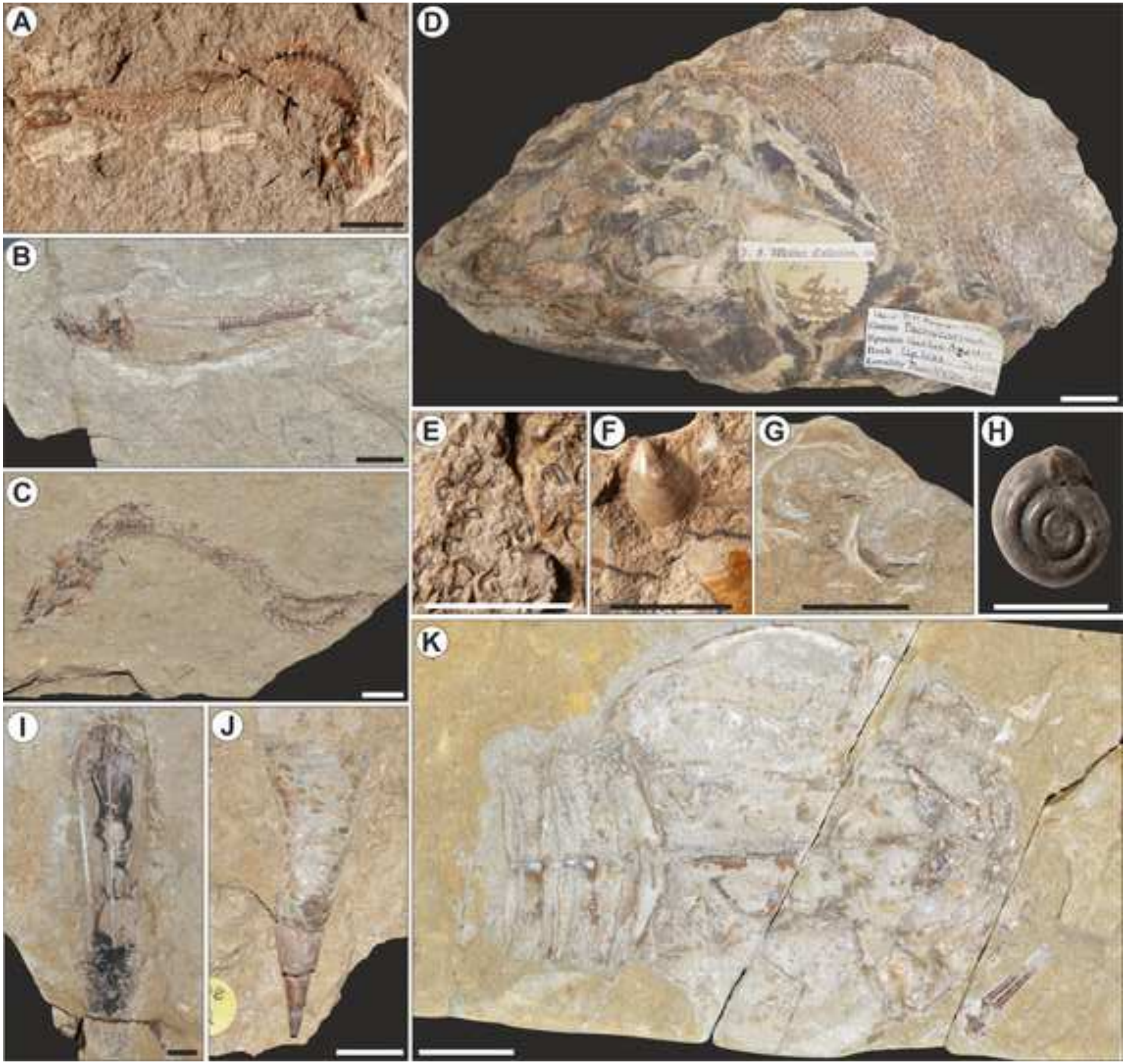

Figure 3

Click here to access/download;Figure;Fig3.tif.tif

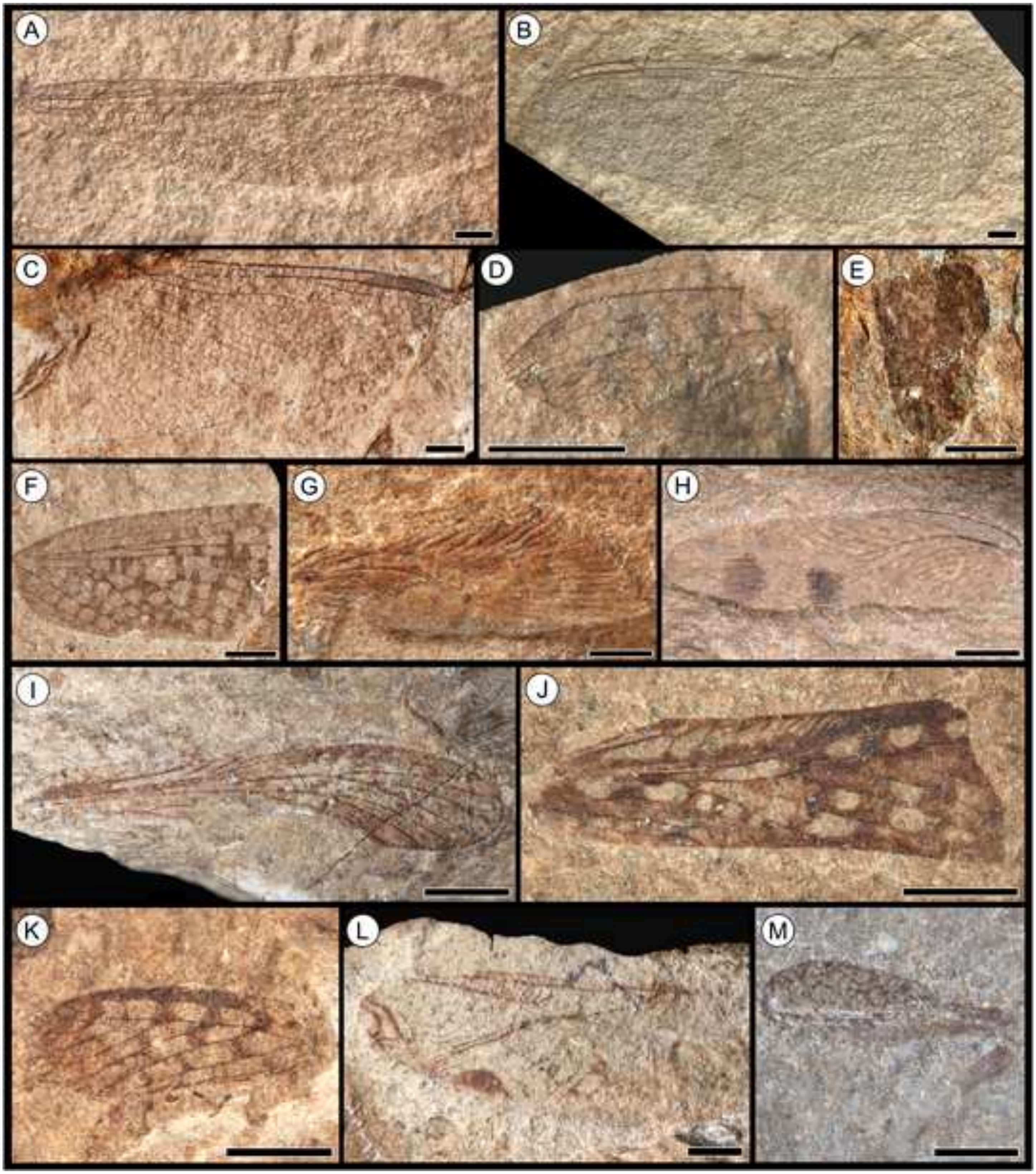

Figure 4 Click here to access/download;Figure;Fig4.tif.tif

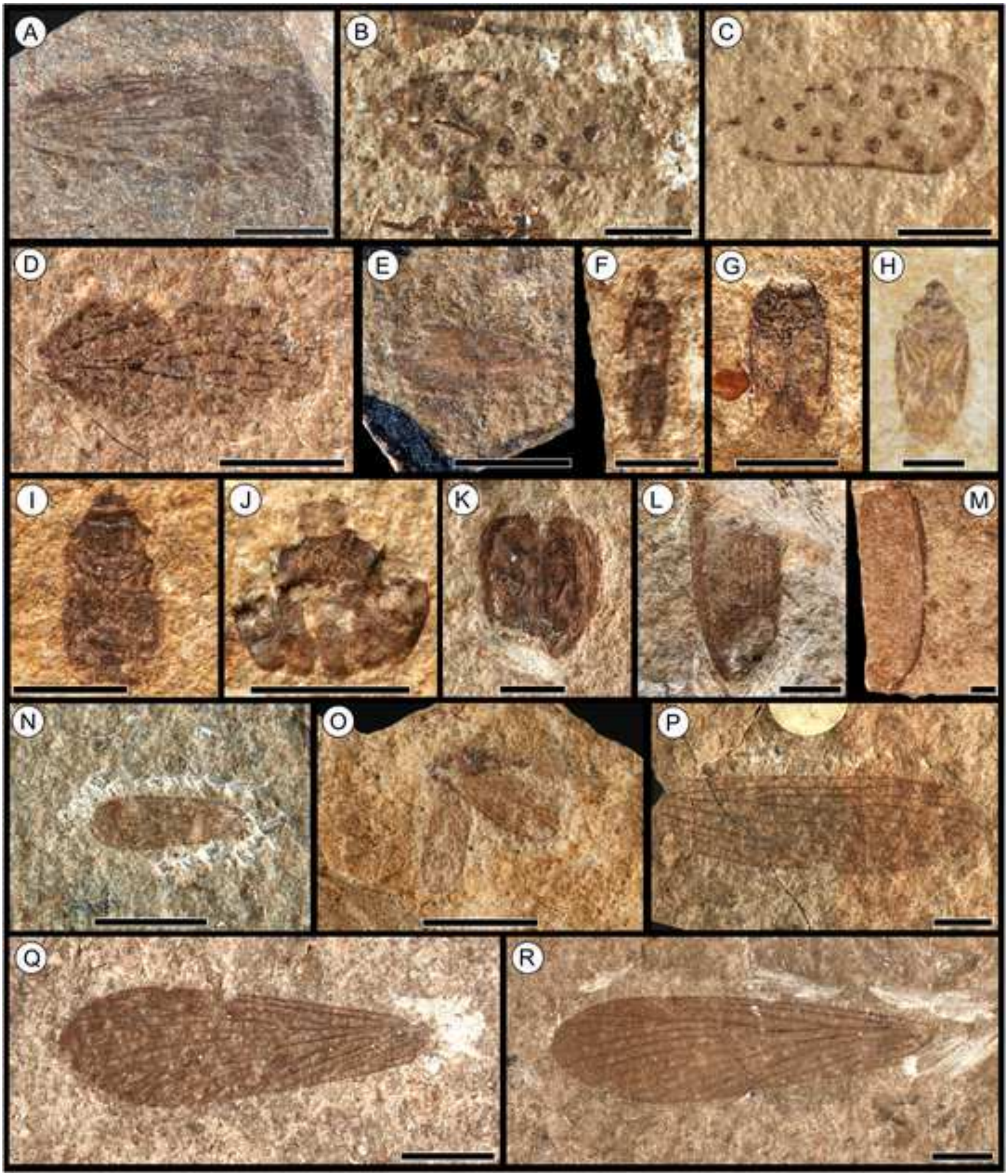

Figure 5

Click here to access/download;Figure;Fig5.tif.tif

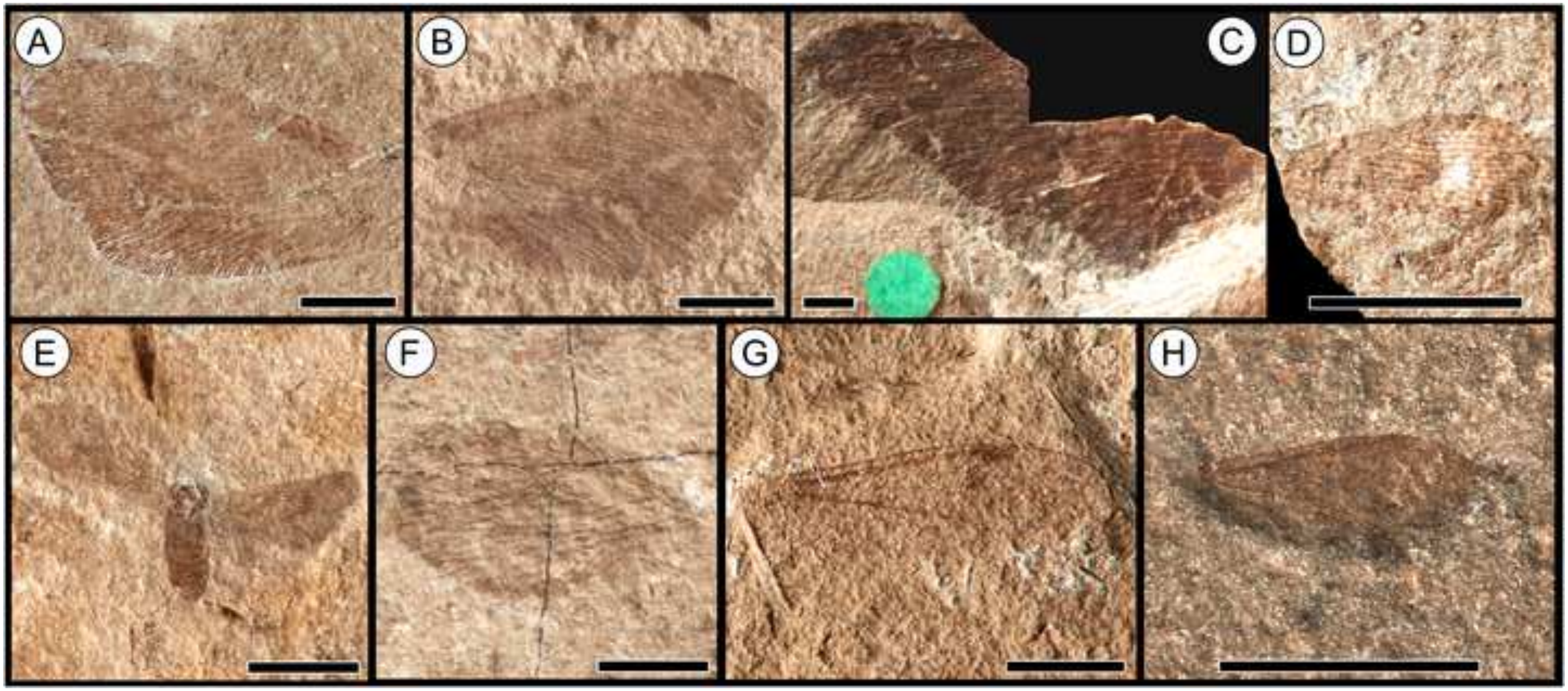

Figure 6

Click here to access/download;Figure;Fig6.tif.tif

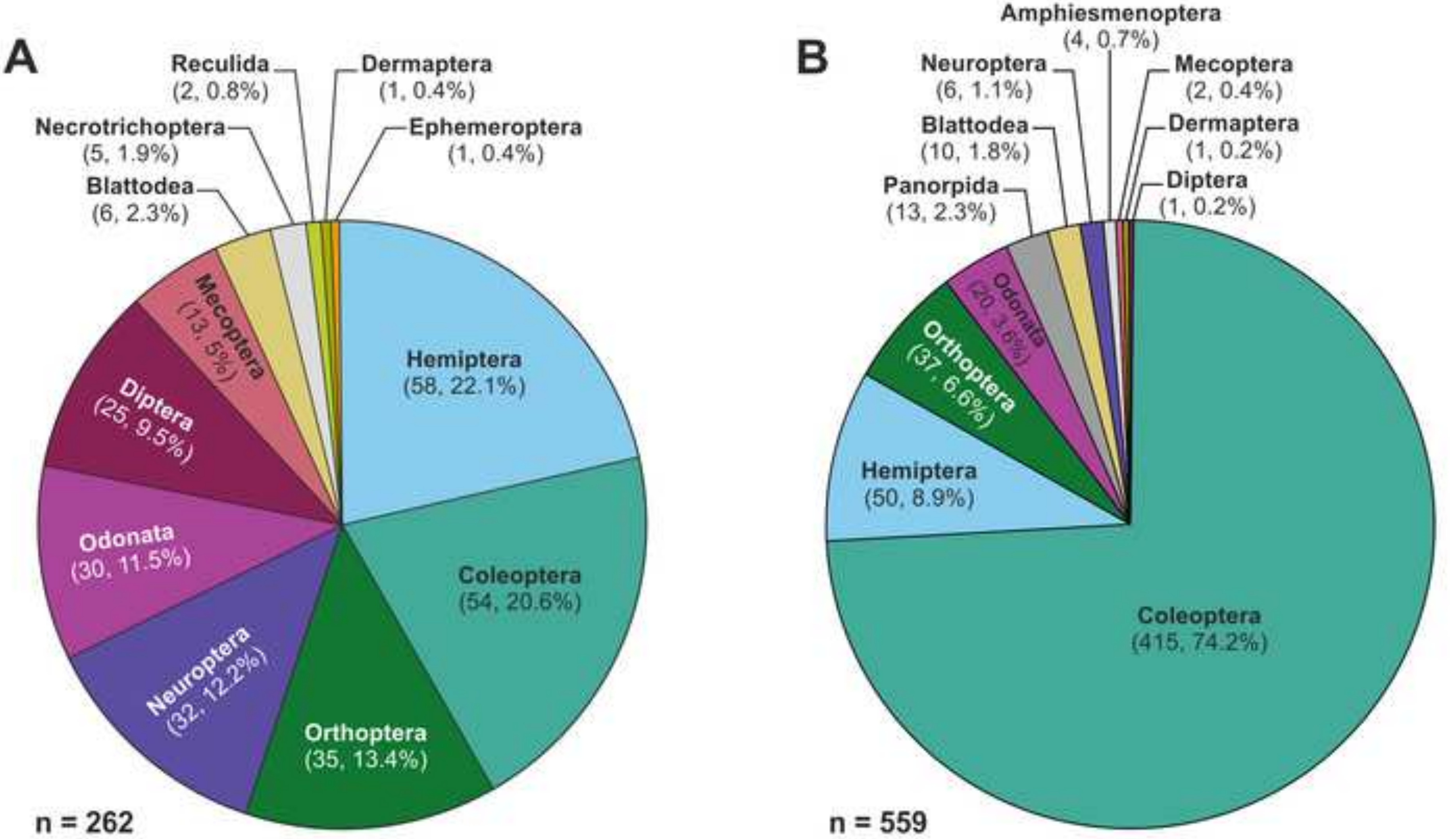

Figure 7

Click here to access/download;Figure;Fig7.tif.tif

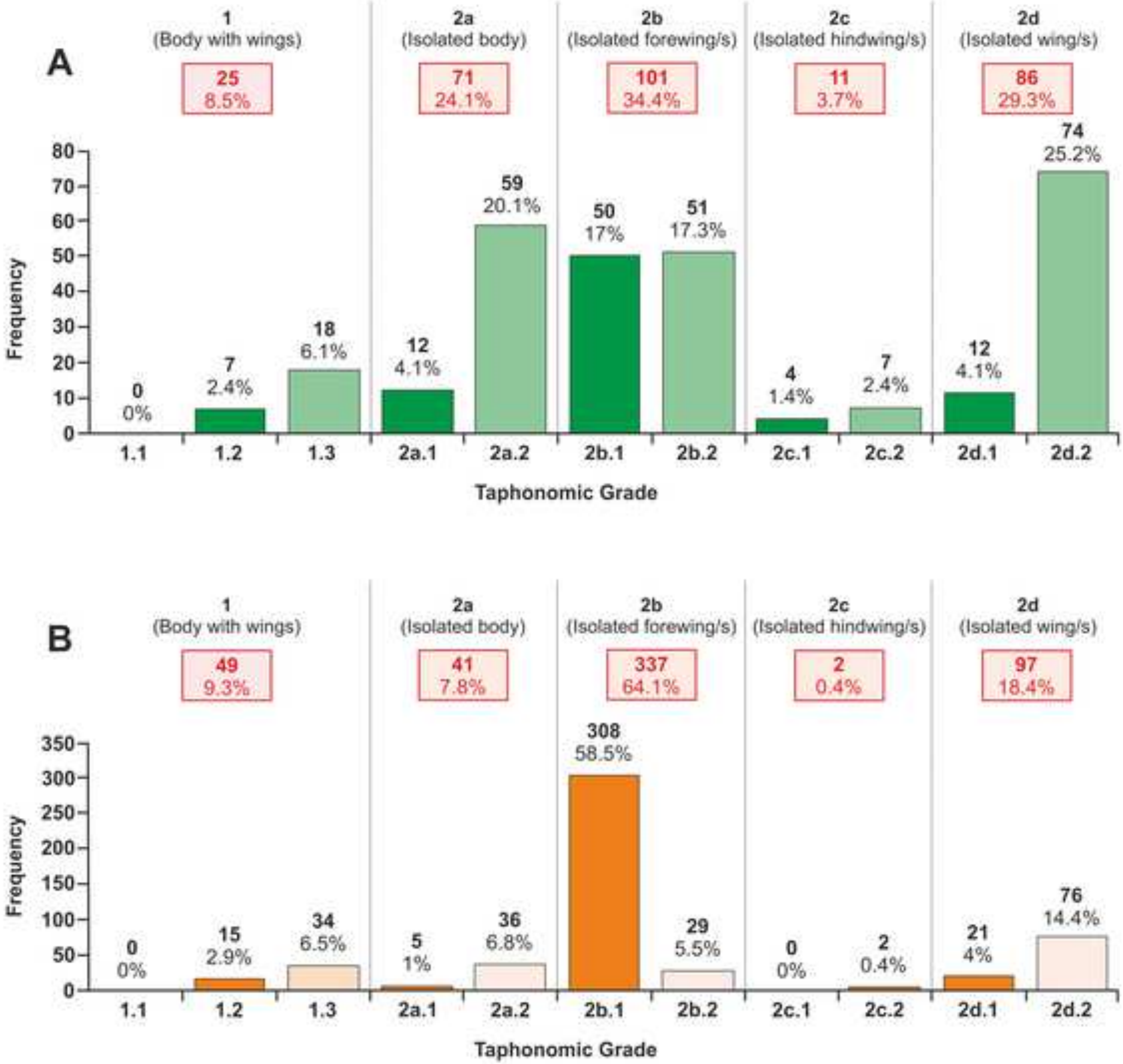

Figure 8

Click here to access/download;Figure;Fig8.tif.tif

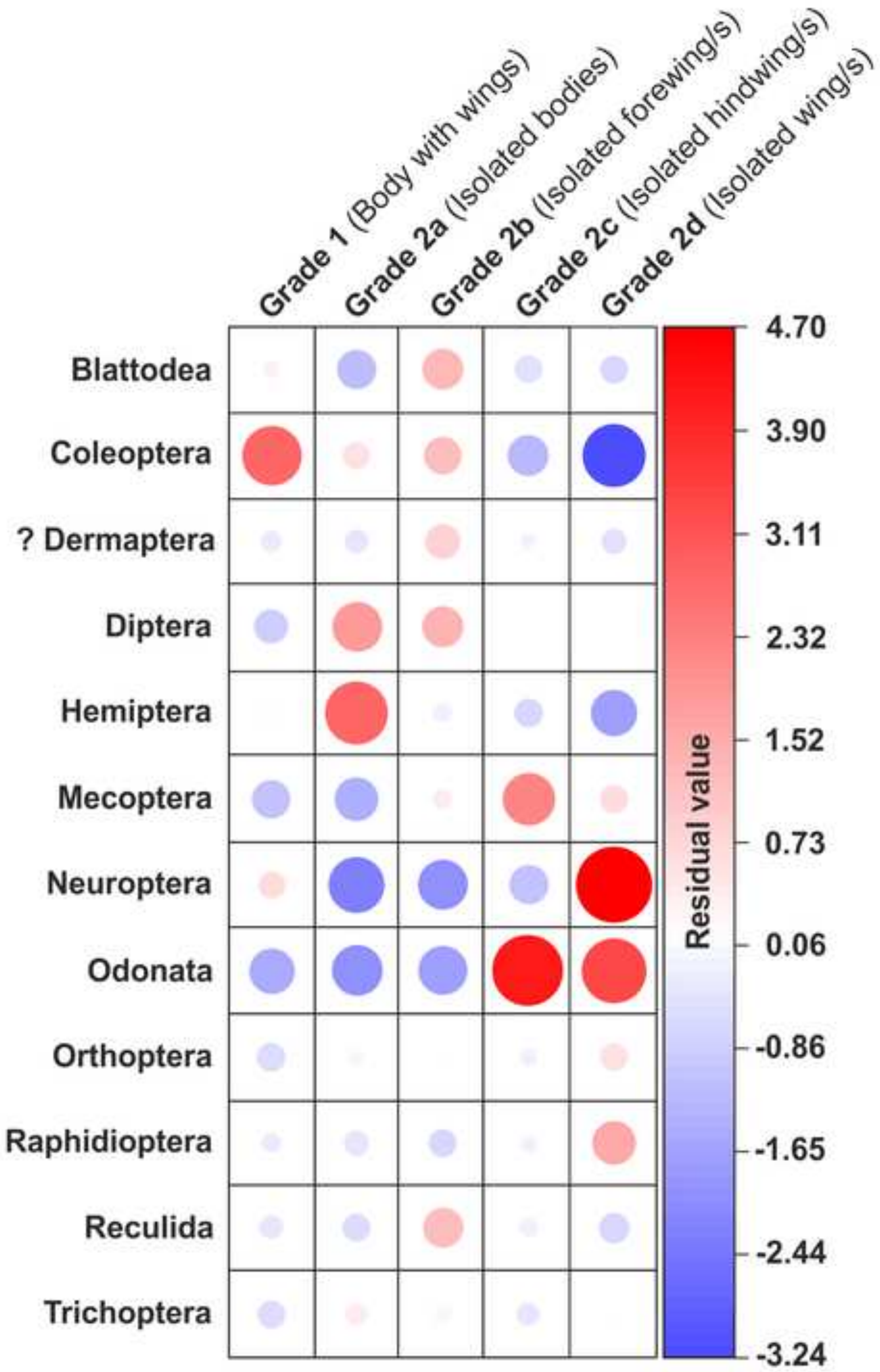

Figure 9

Click here to access/download;Figure;Fig9.tif.tif

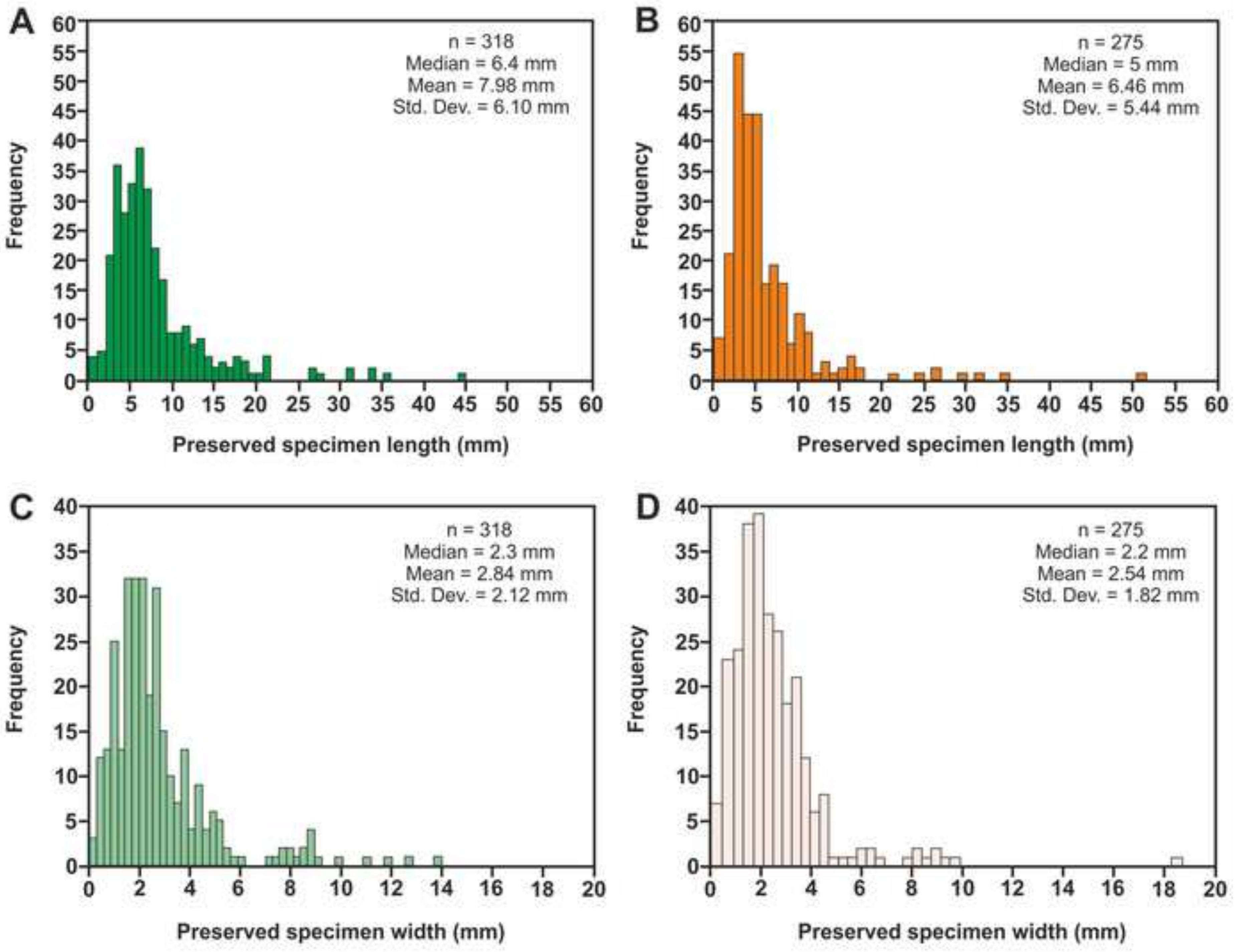

Data Review URL

Click here to download Data Review URL
http://datadryad.org/stash/share/0g_D75vgw0G5e1dMfmQEEEMZYVPT
