# Supplementary material for: The fossil insect assemblage associated with the Toarcian (Lower Jurassic) oceanic anoxic event from Alderton Hill, Gloucestershire, UK
Source: PLoS One. 2024 Apr 17;19(4):e0299551. doi: 10.1371/journal.pone.0299551 (PMC11023202; doi:10.1371/journal.pone.0299551)
Supplement: Attachment — Submitted filename: Swaby et al. Response to Reviewers.docx [file pone.0299551.s002.docx]

**Responses to reviewers and academic editor comments on:**

**Swaby et al. The fossil insect assemblage associated with the Toarcian (Lower Jurassic) Oceanic Anoxic Event from Alderton Hill, Gloucestershire, UK.**

We are grateful for the two helpful and very positive reviews of our manuscript and also the editors points and handling of the manuscript. We are delighted that only minor revisions have been suggested. As requested, we are resubmitting:

- This rebuttal letter that responds to each point raised by the academic editor and two reviewers;
- a marked-up copy of your manuscript that highlights changes made to the original version, entitled 'Swaby *et al.* Revised Manuscript with Track Changes'
- an unmarked version of your revised paper without tracked changes, entitled 'Swaby *et al.* Manuscript’.

Line numbers in black refer to the original PDF that was submitted and reviewed. Line numbers in blue and brackets refer to those in the Revised Manuscript with track changes.

**Responses to journal requirements / academic editor comments**

**1. PLOS ONE's style requirements:** We have checked through PLOS ONE's style requirements, including those for file naming, and have ensure our manuscript adheres to these.

**2. Additional specimen information and permits:** We have checked and ensured that all specimens have complete repository information, including museum name and geographic location. No permits were required for this work, and the statement ‘No permits were required for the described study, which complied with all relevant regulations’ is included after the acknowledgements (L1616) (L1677).

**3. Repository information**: We will provide the relevant accession numbers or DOIs to the Dryad database where we have put the data once the manuscript is accepted.

**4. Copyright of Fig.1:** ‘A’ was modified from figure A in Dera *et al.* (2009). We have contacted Elsevier in regards to the reuse of this figure, and subsequently filled out the RightsLink image reuse form and paid the copyright clearance fee to reuse a modified version of the figure in this manuscript (receipt attached). We have also received written confirmation from Elsevier that I can reuse the image under the CC BY 4.0 license. ‘B’ was modified from figure B in Ruebsam and Al-Husseini (2020) which is an open access paper published in Gondwana Research (Elsevier) and was published under a CC-BY license. The CC license states that we are free to copy and redistribute the material in any medium or format for any purpose, even commercially; and that we are free to adapt, remix, transform, and build upon the material for any purpose, even commercially.

**5. Reference list:** We have updated and reviewed the reference list to ensure that it is complete and correct.

**Responses to the comments from the reviewers**

**Reviewer #1**

Many thanks for the careful and positive review.

Reviewer: Only assemblages from Germany have been studied in detail. In Luxembourg too.

Authors response: We have added Luxembourg to L26 (L27) in the abstract.

Reviewer: Indeed, in extant assemblages, orthoptera are not so common, less than Diptera and Hymenoptera, thus the sentence juts before is problematic, maybe?

Authors response: We can see how our original wording might have been misinterpreted so we have reworded the sentence about the ordinal diversity and in the next sentence clarified that the commonest orders pertain to Alderton Hill (L36). We agree that Orthoptera is notably more common in the Toarcian assemblages, and we discuss this further in the manuscript (L1518 – L1521).

Reviewer: The three main collections, although rather large, are not so important compared to the potential diversity of insects, that the percentages as given, can be taken without caution. That is, if, it was possible, to make random sub-collections within these collections, to establish at least mean values and rudimentary statistical information for each order. This would increase the significance of the percentages that you use as I have seen in many other outcrops, collections of thousands of insect fossils need to be made in order to have rather stable percentages of the orders as you base all your study on these percentages, it would better if you could make such statistical analyses.

Authors response: We have undertaken further statistical analysis in the form of rarefaction at each taxonomic level (order, family, genera and species) to investigate the completeness of the sampling at the two localities. We found that the rarefaction curves show that the palaeoentomofauna is a good representation of the assemblage preserved at Alderton Hill at order level and that the assemblage is reasonably well sampled at family and genera level. We have revised the methods (L380 – L386) including adding a new reference, results (L1192 – L1212 including a new figure (6)), and interpretation (L1493-L1495 and L1551-L1554).

**Reviewer #2**

Many thanks for the positive comments, we were pleased that this was considered 'a very interesting and important contribution to the knowledge of the Toarcian strata of UK and Europe', that the interpretation was considered 'very good' and the taphonomic work was thought to be 'important' and 'well presented'.

Several points were both in the summary and on the PDF (e.g. Lagerstätte, primitive, minor typographical errors, use of commas as per Article 22 in ICZN and use of the term previous holotype) we provide the response to all of these below in the section 'Response to PDF comments'.

Reviewer: A few minor changes are necessary, improvement, e.g. use of German word Lagerstätte, should be in majuscule first letter as German noun.

Authors response: Corrected see response to L197 (L208) and L221 (L233) in the **Response to PDF comments**

Reviewer: in the Jurassic most of the lineages mentioned were specialized forms so use of 'primitive' in context of old insect lineages seems to be awkward.

Authors response: Corrected to basal lineages see also response to L367 (L390 – L391) in the **Response to PDF comments**

Reviewer: there are some minor typographic errors, etc.

Authors response: Many thanks for identifying these we have corrected them and thoroughly checked the manuscript, see **Response to PDF comments**.

Reviewer: The main concern of the manuscript is usage of scientific names. Names in full with author(s) and year of description should be given with comma separating Author(s) name and year - it is written in Article 22 of the ICZN Code and also pointed in Recommendations of ICZN. This should be checked and improved throughout the text.

Authors response: Many thanks we have now corrected all of these.

Authors used expression "previous holotype", which is very odd - a holotype of species, even if the name is synonymized remains a holotype - this expression should be changed throughout the text.

Authors response: See response to L307 (L419) to L726 (L761) in the **Response to PDF comments.**

Reviewer: If *'Elcana'* is no longer available as generic name (put in synonymy), the family name 'Elcanidae' probably should be changed or conserved - is any decision of ICZN commission made? I cannot find any note on it. If *Elcana* is synonymized under *Panorpidium*, these nomenclatorial combinations should be used. Please make these decisions clear, if you resurrecting *Elcana* from synonymy for a few species? The you have to give the concept and content of the taxon under this name.

Authors response: See response to L546 and L549 (L561 – L582).

Reviewer: If you decided to use the traditional name Auchenorrhyncha (which is a paraphyletic assemblage), please give the reasons for this treatment.

Authors response: See response to L599 (L631). We've also updated L1045-L1047.

Reviewer: Fulgoridiidae (another paraphyletic assemblage) were recently treated by Poinar et al. (2022), Bourgoin & Szwedo (2022, 2023), Zhao *et al.* (2023), so please refer to these works for discussion.

Authors response: See response to L612 (L642 – L644).

Reviewer: Taxonomic actions presented in theses are not valid according to rules of ICZN - if you agree with these decisions, it must be clearly stated in your paper and you are reviewers of these taxa.

Authors response: See response to L633 (L667).

**Response to the PDF comments in the order of the annotations**

We are grateful to the reviewer for the detailed editorial comments on the manuscript.

L197 (L208) and L221 (L233): We have capitalised ‘Lagerstätte’ and deleted the second mention as it was unnecessary.

L275 (L288): We are unsure why this is highlighted as no comment was left but we clarify that Canon 5DS R is the make and model of the camera.

L289 (L303) We've added a comma between the name of author and year of description here and throughout the manuscript following zoological nomenclature convention. We thank the reviewers for pointing this omission out.

L297 (L315): Changed *‘Incertae sedis’* to *‘incertae sedis’* throughout the manuscript.

L307 (L321), L396 (L419), L398 (L421), L402 (L426), L490 (L515), L558 (L590), L625 (L659), L626 (L659), L726 (L761): Changed ‘previously holotype’ to holotype as advised.

L367 (L390 – L391): Changed ‘most primitive’ to basal linages.

L399 (L431): For consistency with the rest of the taxonomy, in the remarks we've added a reference to Ansorge (1999) for the extensive synonymy list.

L488 (L513), L501 (L514), L512 (L516), and L517: Changed from tegmin to tegmen.

L546 (L572): We have clarified in the manuscript that we are not resurrecting the genus *Elcana*, we are just using the name “*Elcana*” as a placeholder until a revision is undertaken.

L549 (L580 – L582): We have clarified in the manuscript that we are the ones that are making *Archelcana tina and A. numbergerae* synonymous with *“Elcana” geinitzi (tina)* and *“Elcana” media (numbergerae)* respectively*.*

L599 (L631) and also L1045 and L1341: We have used the new classification system in Bourgoin & Szwedo (2023) as suggested by the reviewers, and replaced Auchenorrhyncha Duméril 1805 for Fulgoromorpha Evans, 1946 in the text and reference list.

L612 (L642 – L644): To improve the clarity, we have re-ordered the sentences and amended wording by removing 'and the related genera and subgenera' and adding 'From the Toarcian'. We only intended to refer to the genera of Fulgoridiidae from the Toarcian which are currently well-defined, and not of all the genera of Fulgoridiidae thus now we have clarified the sentence we don't think discussion on Fulgoridiidae from Poinar *et al.* (2022), Zhao *et al.* (2023), Bourgoin & Szwedo (2022, 2023) is relevent.

L620 – L622 (L668 – L677): All of the relevant papers have been referred to in this synonym – the FLOW database (Bourgoin 2023) was also checked as suggested, but doesn’t reference either *M. brodiei, M. woodwardi* or *M. bulleni.* Therefore, we've retained our original wording.

L633 (L667): We have amended the remarks to comply with the ICZN guidelines, whilst noting that the suggestion was originally made in an unpublished PhD thesis.

L633-L634 (L668 – L669): Changed from Handlirsch (1908) to Handlirsch (1906).

L960 (L997): Changed from Kelly *et al.,* 2016 to Kelly, Ross and Jarzembowski, 2016

L981 (L1018): There is no nomenclatorial issue with using the family Elcanidae as suggested by the reviewer, so this taxon has been retained.

L1143 (L1181): Missing author and year (Hinton, 1958) added.

L1180 (L1240 – L1241): Whilst the reviewer suggested the identification of this material might be refined further (i.e. at least to infraorder level) the preservation is so poor that it is not possible to do this. We have clarified this issue in the manuscript.

Table 5 (L1254): We have inserted a hyphen to indicate where to break Pseudopolycentropodidae and *Pseudopolycentropodus*.

L1496 (L1571 – L1573): We have clarified the previous sentence where we covered the potential for reworking and more turbulent conditions.

L1545 (L1626): Updated ‘Pacycormids’ to pachycormids.

L1838 (L1929), L1839 (L1930), L1844 (L1935), L1860 (L1951), L1864 (L1954) and L1870 (L1960): Italicised *Architipula*, *Grimmenia*, *Madreporia, Pelagosaurus typus, Pachycormus* and *Gabaleryon* respectively.

L1903 (L1996): Changed new species to lowercase and italicised *Eryon*.

L1905 (L1998): Changed monograph to lowercase.

L1908 (L2002), L1911 (L2005), 1912 (L2006): Italicised *Leptolepis normandica*, *Cyprinus coryphaenoides* and *Tetragonolepis semicinctus* respectively*.*

L1914 (L2008 – L2009): Reformatted reference to be lowercase.

L1918 (L2012): Changed xviii to XVIII

L1927 (L2022): Changed ngn sp., to n.g.n. sp.

L1948 (L2043 – L2044), L1962 (L2059 – L2061): Reformatted references to be lowercase.

L1979 (L2082): Italicised *Leptolepis*

L1983 (L2085): Changed palaeogeographische to paläogeographische.

L2002 (L2106 –L2108): Reformatted reference to be lowercase

L2009 (L2114): Italicised *Locustopsis kruegeri.*

L2017 (L2126 – 2127): Reformatted reference to be lowercase and changed orthoptera to orthopterans.

L2023 (L2133): Italicised *Archelcana*.

L2031 (L2144 – 2146): Changed Известия Российской академии наук. Серия математическая to Izvestiya Rossiiskoi Akademii Nauk. Seria matematicheskaya.

L2039 (L2153): Capitalised Liassic.

L2041 (L2155 – 2156): Removed “” from reference.

L2023 (L2163): Italicised *Necrotaulius*.

L2072 (L2188): Changed ‘Taxa’ to lowercase.

L2089 (L2210), L2115 (L2242): Italicised *Mesotipula* and *Tribolium castaneum* respectively

**Additional updates to the manuscript by the authors:**

We have updated the number of taxa to account for a new genera and species present within the Alderton Hill insect assemblage, which is being described in another manuscript. The amends relating to this are:

L33, Table 4, L1642 and L1661: Increased the number of families to from 20 to 21 and the number of genera to from 22 to 23.

L33 and Table 4: Increased the number of genera identified to from 5 to 6.

Table 4 and L1642, L1661: Increased the number of individuals assigned to family from 138 (37.3%) to 139 (37.6%).

We have also:

Checked and amended where necessary the singular/ plural of taxon, genus, tegmen and that/which throughout.

L33-L35, L36, L41, L42, L45, L55: Minor editing to improve the wording.

L84: Deleted a reference that was incorrect and not needed.

L96-L97: Clarified the sentence to avoid repetition.

L110-L112, L114, L121-L122, L124, L135: Minor editing to improve the wording.

L164-165 and L186-L187: Clarified the references for the ammonites and updated Fig 2G to provide an image of a more diagnostic specimen previously unfigured in the literature.

L193-L198: Minor editing to improve the wording.

L209-L211: We've provided an extra sentence on the lithology at Strawberry Bank as this appears to have affected the preservation of this palaeoentomofauna.

L213: Minor editing to improve the wording.

L217-L220: We've added the full names of the fossil collectors for clarity.

L255: Minor editing to improve the wording.

L255-L256: Added the full name for clarity and consistency with L217-L220.

L265-L266: Clarified that it is bias during identification rather than collection.

L306: Date of Geinitz corrected from 1888 to 1887.

L312-L313: Brackets added after the taxa citation to indicate transfer to another genus.

L326-L328: Moved the text relating to permission to photograph a specimen as it was in the wrong place.

L522: Corrected Caloblattinidae to *incertae sedis* as recent research shows that the taxonomy of this specimen, including at family level, is not yet established.

L429-L430, L450-451: Improved the description of the feature in the wing venation that can be confirmed as identical.

L628-L629: Clarified the wording about taxonomic level.

Table 4: For Strawberry Bank, we have decreased the number of insect genera identified from 7 to 6 because we reassigned all specimens previously attributed to *Fulgoridulum* sp. by Williams *et al.* 2015 to *Margaroptilon* sp. See also L1055 for discussion.

L782, and figure 4 caption: Measurement of the wing updated to take into account the new composite image of the part and counterpart in Figure 4.

L829: The description of the venation in *Actinophlebia* has been clarified, from ‘it was thought that the Sc is running free into the costal margin’ to 'the Sc appears to run free into the costal margin'.

L1477-L1481: Clarified the wording for the broad similarity between the palaeogeography of Alderton Hill and the geography of the Azores.

L1495, L1525, L1533, L1557, L1567, L1570, L1580, L1584, L1589, L1594-L1596, L1601, L1601-L1602: Minor editing to improve the wording and clarity.

L1604 to L1605 and L1616-L1619: Slight rewording to clarify our discussion about juvenile insects.

L1621-1622, L1641, L1647 and L1676-1677: Minor editing to improve the wording and clarity.

L1690-L1699: Added TTNCM (Amal Khreisheh) and Mike Simms to acknowledgments. Acknowledged the reviewers.

L1700-L1701: Added wording to explain the contribution of one author at their request.

L1786-L1789: Replaced [26] with a more up to date reference that also provides and refers to a broader set of evidence.

L1818-L1817: Replaced one of the references to give a broader perspective.

We have updated the reference list and citation numbers throughout the manuscript to include additional references suggested by the reviewers, in addition to removing those that are no longer needed. We have itemised the reference changes in the detailed comments.

We have updated the figure numbers to account for the new figure 6 that presents the results of the rarefaction analysis that was added in response to reviewer 1's comments.

Made slight changes to Fig. 4 to improve the clarity of the images.

Corrected hypens to en dashes throughout the manuscript.
