# Supplementary material for: The fossil insect assemblage associated with the Toarcian (Lower Jurassic) oceanic anoxic event from Alderton Hill, Gloucestershire, UK
Source: PLoS One. 2024 Apr 17;19(4):e0299551. doi: 10.1371/journal.pone.0299551 (PMC11023202; doi:10.1371/journal.pone.0299551)
Supplement: Attachment — Submitted filename: Swaby PONE review.docx [file pone.0299551.s003.docx]

**Review of Swaby et al. ‘Fossil insects associated with the Toarcian Oceanic Anoxic Event from Alderton Hill, UK’**

This review is of a revised manuscript that was reviewed by others previously.

The paper is a thorough assessment of the Toarcian insect fauna of the UK and deserves to be published. However, I have spotted some things that could be improved, additional references that would be worth citing and some corrections, thus I am recommending minor revision.

Line 97, ref. 68 is 20^th^ Century.

Line 107, the thylacocephalans from Strawberry Bank were mentioned by-

Laville, T., Forel, M.-B. & Charbonnier, S. 2023. Re-appraisal of thylacocephalans (Euarthropoda, Thylacocephala) from the Jurassic La Voulte-sur-Rhône Lagerstätte. *European Journal of Taxonomy*, **898**, 1-61.

Lines 161, 176, ‘*Eryon*’ *richardsoni* is now in the genus *Proeryon*, see-

Audo, D., Schweigert, G. & Charbonnier, S. 2020. *Proeryon*, a geographically and stratigraphically widespread genus of polychelidan lobsters. *Annales de Paléontologie*, **106**, 102376, 1-21.

Line 219, in between ‘collated’ and ‘by’ please insert ‘studied by Andrew J. Ross (National Museums Scotland), a list of preliminary identifications was supplied to the senior author and the fauna was summarised‘ [I spent a significant amount of time identifying these, so deserve more credit]

Line 246, in between ‘by’ and ‘Williams’ insert ‘Ross in’

Lines 293-294, replace ‘*incertae sedis*’ with ‘undescribed’

Line 298, it would be worth mentioning that M is a leg.

Line 308, H looks like it is good enough to be identified or described so I suggest you replace ‘*incertae sedis*’ with ‘undetermined’

Line 370 onwards. This large section entitled ‘Systematic taxonomy’ is not satisfactory because it does not include any detailed descriptions, nor does it mention the diagnostic characters that justify the identification of the specimens. This is little more than a catalogue of identified taxa with comments, so should be re-titled accordingly.

Lines 502-510, this is being described elsewhere (paper in prep.) so replace ‘*incertae sedis*’ with ‘uncertain’ and ‘gen. et sp. nov.’ with ‘undescribed’ as it is not being named here. Replace the last sentence with ‘The family placement of this specimen is uncertain at present.’

Lines 529, 972, additional references are-

Zessin W. 1983. Revision der mesozoischen Familie Locustopsidae unter Berücksichtigung neuer Funde (Orthopteroida, Caelifera). *Deutsche Entomologische Zeitschrift*, **30**, 173–237.

Gorochov, A.V. & Coram, R.A. 2023. New and little known taxa of the order Orthoptera (Insecta) from the Upper Triassic and Lower Jurassic of England. 2023. *Palaeoentomology*, **6**(2), 198-204.

Lines 550-553, this is not sufficient for formally synonymising these species. You need to explain in detail the characters that justify the synonymy and provide a synonymy list with the term ‘syn. nov.’ Given that this is not a detailed systematic review of ‘*Elcana*’ I suggest you replace the last sentence with ‘However, these species may prove to be junior synonyms.’

Line 592, I.11405 (Figure 3M) is a leg.

Lines 597-599, only wings are mentioned, what about isolated legs?

Line 732-3, what character and what about it justifies rejecting *Austalius* from Necrotauliidae?

Line 735, A recent paper on necrotauliids that should be added is-

Sukatsheva, I.D. & Sinitshenkova, N.D. 2023. New caddisflies (Insecta: Trichoptera, Necrotauliidae, Philopotamidae) from the Jurassic of Asia and their Triassic ancestors. *Paleontological Journal*, **57**(5), 529-540.

Line 787, replace ‘as’ with ‘a’

Line 881, move ‘Assigned…’ to the next line.

Line 919, ‘Strawberry Bank entomofauna’ should not be in italics.

There is a lot of repetition of the taxonomic names and references as used for Alderton Hill above. I consider it would be better to combine both faunas and then under ‘Assigned material’ make it clear which specimens are from Alderton Hill and which are from Strawberry Bank.

Line 990, add ref. 141.

Line 996, ref. 41 does not mention Protogryllidae.

Line 1025, replace ‘these specimens’ with ‘this specimen’

Line 1135, add ref. 41.

Line 1342, this specimen was examined relatively recently by Thomson (Kelly) for his PhD, so I really hope it is not lost. It is much more likely that it has been misplaced so I suggest you delete ‘and is presumed lost.’

Line 1501, add insectivorous mammals

Line 1529, it seems more likely that the insects came from a tabular limestone; as far I recall none of the specimens have curved surfaces that would indicate they came from nodules.

Lines 1556, 1566, I identified several possible insect larvae from Strawberry Bank (see my preliminary list).

Andrew Ross
