# Supplementary material for: The fossil insect assemblage associated with the Toarcian (Lower Jurassic) oceanic anoxic event from Alderton Hill, Gloucestershire, UK
Source: PLoS One. 2024 Apr 17;19(4):e0299551. doi: 10.1371/journal.pone.0299551 (PMC11023202; doi:10.1371/journal.pone.0299551)
Supplement: Attachment — Submitted filename: Swaby et al. Response to Reviewers.docx [file pone.0299551.s004.docx]

**Responses to reviewers and academic editor comments on:**

**Swaby et al. The fossil insect assemblage associated with the Toarcian (Lower Jurassic) Oceanic Anoxic Event from Alderton Hill, Gloucestershire, UK.**

We are grateful for the three helpful and very positive reviews of our manuscript thus far, and also the editors points and handling of the manuscript. We are delighted that after the 2^nd^ round of review, only minor revisions have been suggested from reviewer 3. As requested, we are resubmitting:

- This rebuttal letter that responds to each point raised by the academic editor and reviewer 3 (as reviewer 2 made no additional comments).
- A marked-up copy of the manuscript that highlights changes made to the original version, entitled 'Swaby *et al.* Revised Manuscript with Track Changes'
- An unmarked version of the revised paper without tracked changes, entitled 'Swaby *et al.* Manuscript’.

Line numbers in refer to the original PDF that was submitted and reviewed. Comments in red are our replies to the individual comments.

**Responses to journal requirements / academic editor comments**

**1. Reference list:** We have updated and reviewed the reference list to ensure that it is complete and correct.

**Responses to the comments from the reviewer 3:**

Many thanks to Andrew Ross for his positive review and the minor corrections.

**L97.** Ref. 68 is 20th Century.

Swapped reference 68 to the 20th century bracket.

**L107.** The thylacocephalans from Strawberry Bank were mentioned by-

Laville, T., Forel, M.-B. & Charbonnier, S. 2023. Re-appraisal of thylacocephalans (Euarthropoda, Thylacocephala) from the Jurassic La Voulte-sur-Rhône Lagerstätte. European Journal of Taxonomy, 898, 1-61.

We have included this reference.

**L. 161, L.176.** ‘Eryon’ richardsoni is now in the genus Proeryon, see-

Audo, D., Schweigert, G. & Charbonnier, S. 2020. Proeryon, a geographically and stratigraphically widespread genus of polychelidan lobsters. Annales de Paléontologie, 106, 102376, 1-21.

We thank Andrew for pointing this out, and have updated reference 99 to Audo *et al.* 2020 as suggested.

**L.219.** In between ‘collated’ and ‘by’ please insert ‘studied by Andrew J. Ross (National Museums Scotland), a list of preliminary identifications was supplied to the senior author and the fauna was summarised‘ [I spent a significant amount of time identifying these, so deserve more credit]

This has been updated as suggested to give Andrew credit. For clarity on which senior author, it now reads - and studied by Andrew J. Ross (National Museums Scotland), a list of preliminary identifications was supplied to the senior author of Williams *et al.* [41] in which the fauna was summarised.

**L.246.** In between ‘by’ and ‘Williams’ insert ‘Ross in’

Updated as suggested by reviewer.

**L.293-294.** Replace ‘incertae sedis’ with ‘undescribed’

Updated as suggested by reviewer.

**L.298.** It would be worth mentioning that M is a leg.

Updated as suggested by reviewer.

**L.308.** H looks like it is good enough to be identified or described so I suggest you replace ‘incertae sedis’ with ‘undetermined’

Updated as suggested by reviewer.

**L.370** onwards. This large section entitled ‘Systematic taxonomy’ is not satisfactory because it does not include any detailed descriptions, nor does it mention the diagnostic characters that justify the identification of the specimens. This is little more than a catalogue of identified taxa with comments, so should be re-titled accordingly.

We agree with this, and have updated this large section to ‘catalogue of identified taxa’ as suggested, and updated the cross references to this chapter throughout the manuscript (i.e. L.1225-1226; L.1279; L.1657).

**L.502-510.** This is being described elsewhere (paper in prep.) so replace ‘incertae sedis’ with ‘uncertain’ and ‘gen. et sp. nov.’ with ‘undescribed’ as it is not being named here. Replace the last sentence with ‘The family placement of this specimen is uncertain at present.’

Updated as suggested by reviewer.

**L.529, L.972.** Additional references are-

Zessin W. 1983. Revision der mesozoischen Familie Locustopsidae unter Berücksichtigung neuer Funde (Orthopteroida, Caelifera). Deutsche Entomologische Zeitschrift, 30, 173–237. Done.

Gorochov, A.V. & Coram, R.A. 2023. New and little known taxa of the order Orthoptera (Insecta) from the Upper Triassic and Lower Jurassic of England. 2023. Palaeoentomology, 6(2), 198-204.

We have added Zessin (1983) as suggested, but Gorochov and Coram (2023) is not relevant to the respective specimen as we cannot identify the specimen beyond genus level.

**L.550-553.** This is not sufficient for formally synonymising these species. You need to explain in detail the characters that justify the synonymy and provide a synonymy list with the term ‘syn. nov.’ Given that this is not a detailed systematic review of ‘Elcana’ I suggest you replace the last sentence with ‘However, these species may prove to be junior synonyms.’

Updated as suggested by reviewer.

**L.592.** I.11405 (Figure 3M) is a leg.

Legs are classed under ‘isolated exoskeletal elements’ within this study – this is explained in table 3 and in the caption to Figure 3 we’ve noted that it’s a leg. See comment for L.298.

**L.597-599.** Only wings are mentioned, what about isolated legs?

Legs are classed under ‘isolated exoskeletal elements’ within this study – this is explained in table 3.

**L.732-3.** What character and what about it justifies rejecting *Austalius* from Necrotauliidae?

The character is ‘no distally bent CuP’ as said in the sentence before, but we have clarified this within the text.

**L.735.** A recent paper on necrotauliids that should be added is-

Sukatsheva, I.D. & Sinitshenkova, N.D. 2023. New caddisflies (Insecta: Trichoptera, Necrotauliidae, Philopotamidae) from the Jurassic of Asia and their Triassic ancestors. Paleontological Journal, 57(5), 529-540.

We have added this reference.

**L.787.** Replace ‘as’ with ‘a’

Updated as suggested by reviewer.

**L.881.** Move ‘Assigned…’ to the next line.

Updated as suggested by reviewer.

**L.919.** ‘Strawberry Bank entomofauna’ should not be in italics.

Updated as suggested by reviewer.

There is a lot of repetition of the taxonomic names and references as used for Alderton Hill above. I consider it would be better to combine both faunas and then under ‘Assigned material’ make it clear which specimens are from Alderton Hill and which are from Strawberry Bank.

Thanks for this suggestion which we have carefully considered, however we prefer not to combine the two faunas to ensure it is clear which taxa belongs to which locality, given that specimens from the different localities share the same specimen prefix.

**L.990.** Add ref. 141.

Updated as suggested by reviewer.

**L.996.** Ref. 41 does not mention Protogryllidae.

We have removed reference 41 from this line as suggested, and clarified where the information was from.

**L1025.** Replace ‘these specimens’ with ‘this specimen’.

Updated as suggested by reviewer.

**L.1135.** Add ref. 41.

Updated as suggested by reviewer.

**L.1342.** This specimen was examined relatively recently by Thomson (Kelly) for his PhD, so I really hope it is not lost. It is much more likely that it has been misplaced so I suggest you delete ‘and is presumed lost.’

At present, we can confirm that this specimen is presumed lost, as the curators and I have searched for it on multiple occasions and could not find it. All that we’ve been able to locate is the box and original associated labels. Therefore we have kept ‘and is presumed lost.’

**L.1501.** Add insectivorous mammals.

Updated as suggested by reviewer.

**L.1529.** It seems more likely that the insects came from a tabular limestone; as far I recall none of the specimens have curved surfaces that would indicate they came from nodules.

The horizon at Strawberry Bank has been reported as a layer of nodules in several references [41, 89]. Furthermore, it’s very rare that fossils have any curvature. So we have retained our original wording.

L.1556, 1566. I identified several possible insect larvae from Strawberry Bank (see my preliminary list).

Updated as suggested by reviewer.
